# Genomic context of *NTRK1/2/3* fusion-positive tumours from a large real-world population

**DOI:** 10.1038/s41698-021-00206-y

**Published:** 2021-07-20

**Authors:** C. B. Westphalen, M. G. Krebs, C. Le Tourneau, E. S. Sokol, S. L. Maund, T. R. Wilson, D. X. Jin, J. Y. Newberg, D. Fabrizio, L. Veronese, M. Thomas, F. de Braud

**Affiliations:** 1 Comprehensive Cancer Center Munich & Department of Medicine III, University Hospital, LMU Munich, Munich, Germany; 2 Division of Cancer Sciences, Faculty of Biology, Medicine and Health, The University of Manchester and The Christie NHS Foundation Trust, Manchester Academic Health Science Centre, Manchester, UK; 3 Department of Drug Development and Innovation (D3i), Institut Curie, Paris & Saint-Cloud, France, Saint-Cloud, France; 4 INSERM U900 Research Unit, Saint-Cloud, France; 5 Paris-Saclay University, Paris, France; 6 Foundation Medicine Inc., Cambridge, MA USA; 7 Genentech Inc., South San Francisco, CA USA; 8 F. Hoffmann-La Roche Ltd, Basel, Switzerland; 9 Department of Medical Oncology and Haematology, Fondazione IRCCS Istituto Nazionale dei Tumori, Milan, Italy; 10 School of Specialisation in Medical Oncology, University of Milan, Milan, Italy

**Keywords:** Cancer, Oncogenes

## Abstract

Neurotrophic tropomyosin receptor kinase (*NTRK)* gene fusions are rare oncogenic drivers in solid tumours. This study aimed to interrogate a large real-world database of comprehensive genomic profiling data to describe the genomic landscape and prevalence of *NTRK* gene fusions. *NTRK* fusion-positive tumours were identified from the FoundationCORE^®^ database of >295,000 cancer patients. We investigated the prevalence and concomitant genomic landscape of *NTRK* fusions, predicted patient ancestry and compared the FoundationCORE cohort with entrectinib clinical trial cohorts (ALKA-372-001 [EudraCT 2012-000148-88]; STARTRK-1 [NCT02097810]; STARTRK-2 [NCT02568267]). Overall *NTRK* fusion-positive tumour prevalence was 0.30% among 45 cancers with 88 unique fusion partner pairs, of which 66% were previously unreported. Across all cases, prevalence was 0.28% and 1.34% in patients aged ≥18 and <18 years, respectively; prevalence was highest in patients <5 years (2.28%). The highest prevalence of *NTRK* fusions was observed in salivary gland tumours (2.62%). Presence of *NTRK* gene fusions did not correlate with other clinically actionable biomarkers; there was no co-occurrence with known oncogenic drivers in breast, or colorectal cancer (CRC). However, in CRC, *NTRK* fusion-positivity was associated with spontaneous microsatellite instability (MSI); in this MSI CRC subset, mutual exclusivity with *BRAF* mutations was observed. *NTRK* fusion-positive tumour types had similar frequencies in FoundationCORE and entrectinib clinical trials. *NTRK* gene fusion prevalence varied greatly by age, cancer type and histology. Interrogating large datasets drives better understanding of the characteristics of very rare molecular subgroups of cancer and allows identification of genomic patterns and previously unreported fusion partners not evident in smaller datasets.

## Introduction

The neurotrophic tyrosine receptor kinase (*NTRK*) genes 1/2/3 encode tropomyosin receptor kinases (TRK) A/B/C respectively. Inter-chromosomal rearrangements causing *NTRK* gene fusions can result in constitutive activation of TRK proteins, which then act as oncogenic drivers through activation of cellular growth pathways^1–3^. *NTRK* gene fusions occur in ~0.3% of all solid tumours, though frequencies vary by cancer type^4–6^. Their prevalence is >90% in rare cancers such as secretory breast carcinoma and mammary analogue secretory carcinoma of the salivary gland (MASC)^7,8^.

Small molecule TRK inhibitors (entrectinib and larotrectinib) are clinically active in *NTRK* fusion-positive tumours^9,10^. Retrospective analysis of data from >26,000 patients from a prospective genomic screening programme at Memorial Sloan Kettering Cancer Center (MSKCC, NY, USA) investigated the incidence, distribution and genomic context of *NTRK* gene fusions across cancers^6^. They were found in 0.28% of cases and *NTRK* fusion-positive tumours were largely devoid of other oncogenic drivers.

We aimed to expand these findings by analysing data from >295,000 cancer patients from the FoundationCORE^®^ database (Foundation Medicine Inc., Cambridge, MA, USA) to investigate *NTRK* gene fusions prevalence, co-occurrence with relevant biomarkers/oncogenic drivers, associated fusion partners and cancer types/histologies. Additionally, *NTRK* fusion-positive cases in the FoundationCORE database were compared with those enrolled in three phase I/II entrectinib clinical trials^9^ (ALKA-372-001 [EudraCT 2012-000148-88], STARTRK-1 [NCT02097810], STARTRK-2 [NCT02568267]), to determine if the study cohorts were representative of the real-world population.

## Results

### Solid tumour *NTRK* gene fusion prevalence in the FoundationCORE database

**Fig. 1:**
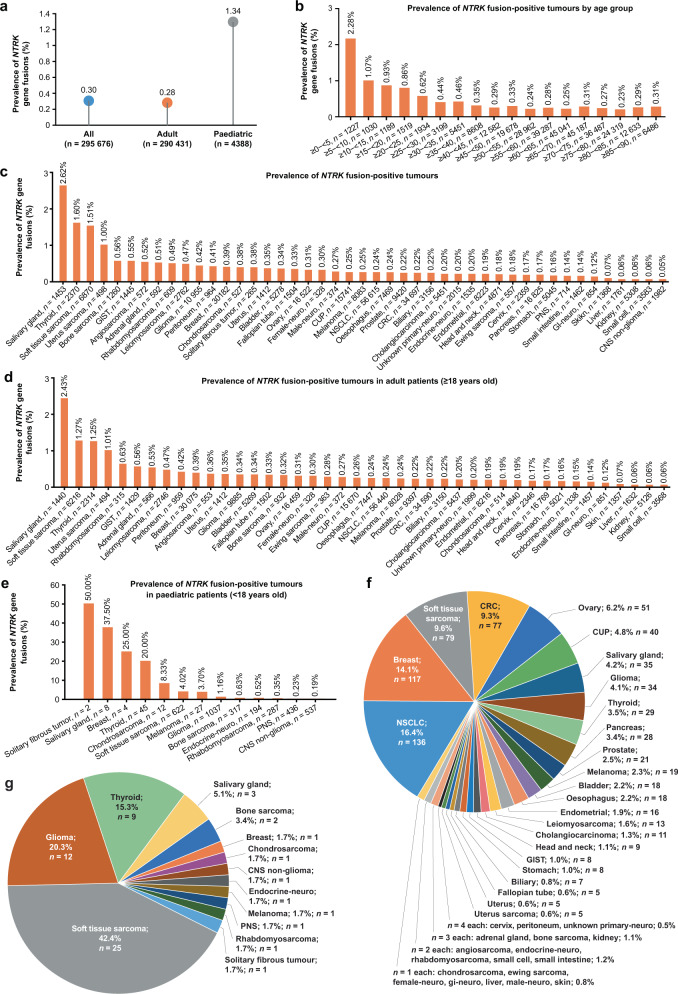
Prevalence of *NTRK* fusion-positive specimens in FoundationCORE by indication and age group. The prevalence of *NTRK* gene fusions overall and among adult (aged ≥18 years) and paediatric patients (aged <18 years; (**a**). The prevalence of *NTRK* gene fusions by age group (**b**). The prevalence of *NTRK* gene fusions by cancer type among: all patients (**c**), adult patients (**d**) and paediatric patients (**e**), with *n* numbers representing the total number of patients analysed per tumour type. Prevalence analysis of cancer types among all *NTRK* fusion-positive tumours in adults (**f**) and paediatric patients (**g**), where numbers represent the total number of patients with each cancer type. CNS central nervous system, CRC colorectal carcinoma, CUP unknown primary carcinoma, GI gastrointestinal, GIST gastrointestinal stromal tumour, NSCLC non-small cell lung cancer, *NTRK* neurotrophic tyrosine receptor kinase, PNS peripheral nervous system.

From 295,676 patients, *NTRK* gene fusions were found in 889 (prevalence = 0.30%, Fig. 1a). Demographics are presented in Table 1 and Supplementary Table 1. The 889 *NTRK* fusion-positive cases included 134 distinct histological subtypes from 45 cancer types (Supplementary Table 2). *NTRK* fusion-positive tumours prevalence varied by age and cancer type (Fig. 1a–e); it was 0.28% in adults (aged ≥18 years) and 1.34% in paediatric patients (aged <18 years; Fig. 1a). Prevalence increased with decreasing age, with children <5 years demonstrating the highest incidence of 2.28% (Fig. 1b; Supplementary Table 3); largely as a result of *NTRK* fusion-positive soft tissue fibrosarcoma (1.06%, *n* = 13/1227 of all patients <5 years), not found in other age groups (Supplementary Table 4).

**Table 1:** Demographics of patients with *NTRK* fusion-positive tumours in FoundationCORE.

	All (*N* = 889)^a^	Adults (≥18 years, *n* = 827)	Children (<18 years, *n* = 59)
Median age, years (range)	60.5 (0–89)	62 (18–89)	5 (0–17)
Female, *n* (%)	511 (57.5)	481 (58.2)	28 (47.5)
Male, *n* (%)	378 (42.5)	346 (41.8)	31 (52.5)
Specimen location, *n* (%)			
Local	338 (38.0)	315 (38.1)	23 (39.0)
Metastatic: lymph node	87 (9.8)	85 (10.3)	2 (3.4)
Metastatic: non-lymph node	198 (22.3)	196 (23.7)	2 (3.4)
Unknown	266 (29.9)	231 (27.9)	32 (54.2)

*NTRK* neurotrophic tyrosine receptor kinase.

^a^The total number does not equal the sum of adults and children because three patients were of unknown age.

In adults, prevalence of *NTRK* fusion-positive cancers was highest in salivary gland cancers (2.43%, *n* = 35/1440), soft tissue sarcomas (1.27%, *n* = 79/6216) and thyroid cancers (1.25%, *n* = 29/2314; Fig. 1d). Among the paediatric cohort, prevalence was highest in solitary fibrous tumours (50%, *n* = 1/2), salivary gland cancers (37.50%, *n* = 3/8), breast tumours (25%, *n* = 1/4) and thyroid tumours (20%, *n* = 9/45), although total numbers were low, as these paediatric cancers are rare (Fig. 1e). *NTRK* gene fusion prevalence was further investigated by tumour histology (Supplementary Table 2): prevalence was highest in MASC (71.43%, *n* = 10/14), unknown primary myoepithelial carcinoma (14.29%, *n* = 1/7) and soft tissue fibrosarcoma (11.76%, *n* = 16/136).

All *NTRK* fusion-positive tumours were analysed by cancer type and frequency for adult (Fig. 1f) and paediatric patients (Fig. 1g). The most common adult cancer types (and most common associated histologies; Supplementary Table 2) were non-small cell lung carcinoma (NSCLC; *n* = 136, of which 95 were adenocarcinoma), breast (*n* = 117, of which 71 were breast carcinoma not otherwise specified [NOS] and 42 were invasive ductal carcinoma), soft tissue sarcoma (*n* = 79, of which 37 were sarcoma NOS and 13 were liposarcoma) and CRC (*n* = 77, of which 73 were colon adenocarcinoma). Among paediatric patients the most common were soft tissue sarcoma (*n* = 25, of which 13 were fibrosarcoma and 6 were sarcoma NOS), glioma (*n* = 12, of which 3 were brain astrocytoma pilocytic, 3 were glioma NOS and 3 were glioblastoma) and thyroid (*n* = 9, all papillary carcinoma). An in silico analysis estimating differences in sensitivity between DNA- and RNA-based NGS assays suggested that, although DNA-based assays may not capture all *NTRK* fusions, the detection rates were nonetheless very high (91% vs. 100%) (Supplementary Table 5). The predicted detection rate for the DNA assay matches closely with those reported in the analytic validation of FoundationOne® CDx (Foundation Medicine Inc., Cambridge, MA, USA) for the detection of *NTRK* fusions^11^. Reduced detection rates for *QKI:NTRK2* and *ETV6:NTRK3* variants II and IV were attributed to limited intronic baiting of *NTRK2* and *ETV6* by FoundationOne CDx.

### Spectrum of *NTRK* gene fusion partners detected in solid tumours

**Fig. 2:**
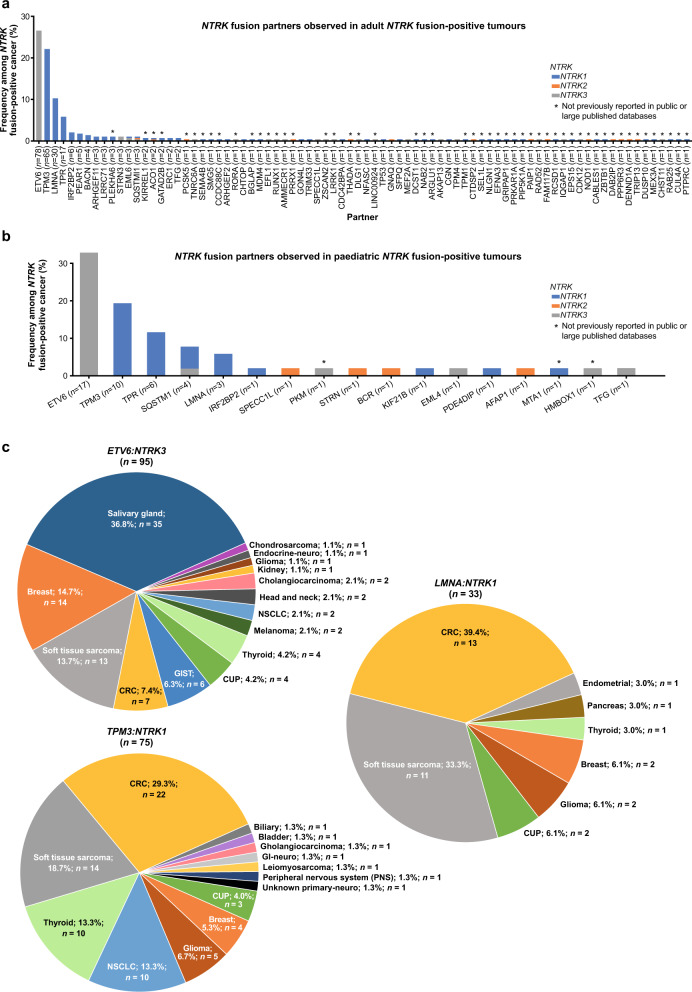
The spectrum of *NTRK* fusion partners detected among *NTRK* fusion-positive solid tumours. Breakdown of *NTRK* gene fusions detected among adult (**a**) and paediatric patients (**b**) and the disease breakdown of the three most frequently observed *NTRK* fusion partners (**c**). CRC colorectal carcinoma, CUP unknown primary carcinoma, GI gastrointestinal, GIST gastrointestinal stromal tumour, NSCLC non-small cell lung cancer, *NTRK* neurotrophic tyrosine receptor kinase.

Eighty-eight unique fusion partner pairs were identified, of which 65.9% (*n* = 58/88) were not previously reported in other large public databases/studies^4–6,8,9,12,13^ (Fig. 2a and b; Supplementary Tables 6 and 7). *ETV6:NTRK3* was most common in adults (26.4%, *n* = 78/295 [total cases with known fusion partners]) and paediatric patients (32.7%, *n* = 17/52 [total cases with known fusion partners]). From all cases with known fusion partners, *ETV6:NTRK3* (27.2%, *n* = 95/349), *TPM3:NTRK1* (21.5%, *n* = 75/349) and *LMNA:NTRK1* (9.5%, *n* = 33/349) were the most common. The most common tumour type with *ETV6* was salivary gland (36.8%), with *TPM3* was CRC (29.3%) and with *LMNA* was CRC (39.4%; Fig. 2c).

### Prevalence of *NTRK* gene fusions by predicted ancestry

Prevalence of *NTRK* fusions was marginally higher in patients with primarily Asian (East and South Asian) ancestry (0.40%) compared with Central/South American (0.37%), African (0.34%) or European (0.28%) ancestry (odds ratio = 1.36; *P* < 0.017; Supplementary Fig. 1). Supplementary Table 8 summarises tumour types by ancestry. NSCLC made up 24% of the East Asian total population but was only 13–20% in other ancestries. Central/South American ancestry was enriched for *NTRK* fusion-positive soft tissue sarcoma (24% versus 10–13% in other ancestries).

### Co-alteration patterns of *NTRK* gene fusions with cancer-related genes

**Fig. 3:**
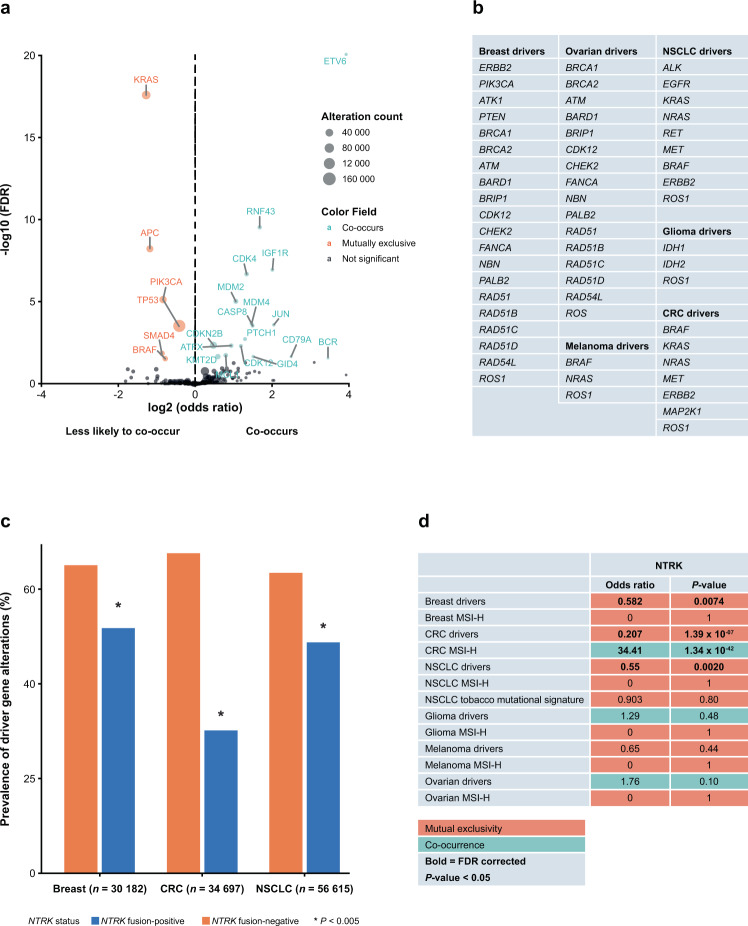
Co-occurrence of genes among *NTRK* fusion-positive cancers. Co-occurrence of genes in all *NTRK* fusion-positive cancers (**a**). The prevalence of gene mutations was compared for *NTRK* fusion-positive and fusion-negative disease. Co-occurrence refers to genes that occurred in *NTRK* fusion-positive disease with an odds ratio greater than 1 compared with *NTRK* fusion-negative disease and the false discovery rate (FDR)-adjusted *P*-value was <0.05. Lack of co-occurrence refers to genes that did not occur in *NTRK* fusion-positive disease with an odds ratio less than 1 compared with *NTRK* fusion-negative disease and the FDR-adjusted *P*-value was <0.05. List of known disease-specific driver genes for different tumour types (**b**). The frequency of mutations found within driver genes listed in **b** in *NTRK* fusion-positive and *NTRK* fusion-negative colorectal cancer (CRC), breast cancer and non-small cell lung cancer (NSCLC; **c**). Summary of co-occurrence and mutual exclusivity of driver gene mutations and microsatellite instability-high (MSI-H) status with specific *NTRK* fusion-positive cancers (**d**). *NTRK* neurotrophic tyrosine receptor kinase.

Across all solid tumours, *NTRK* gene fusions were less likely to co-occur with mutations in *KRAS*, *APC*, *TP53* and *PIK3CA* (*P* < 0.01; Fig. 3a). There was significant co-occurrence of *NTRK* gene fusions with alterations in 14 genes, including *ETV6, RNF43*, *IGF1R*, *CDKN2B* and *CDK4* (Fig. 3a). Co-occurrence with *ETV6* correlated with it being the most common fusion partner. No enrichment was seen with alterations in other clinically relevant biomarkers such as *EGFR*, *ERBB2*, *RET*, *ALK* or *MET*. Supplementary Table 9 summarises results for all genes tested for co-occurrence, providing insight into the genomic landscape of *NTRK* gene fusion-positive cancers.

### Co-alteration patterns of *NTRK* gene fusions with altered driver genes in select tumour types

Analysis of *NTRK* gene fusions and known oncogenic driver genes (Fig. 3b) for breast, ovarian, melanoma, NSCLC, glioma and CRC showed *NTRK* gene fusions were mutually exclusive with alterations in disease-specific driver genes in breast, CRC and NSCLC (*P* < 0.01; Fig. 3c, d; Supplementary Tables 10 and 11) and trended toward mutual exclusivity in melanoma (Fig. 3d). Importantly, there was no mutual exclusivity based on the presence of a tobacco trinucleotide mutational signature in NSCLC (Fig. 3d). Likewise, median tumour mutational burden (TMB) was similar in *NTRK* fusion-positive and fusion-negative tumours, including those in NSCLC, but was increased in *NTRK* fusion-positive CRC (Supplementary materials; Supplementary Fig. 2).

### Evaluation of microsatellite instability (MSI) status in *NTRK* fusion-positive versus *NTRK* fusion-negative tumours

**Fig. 4:**
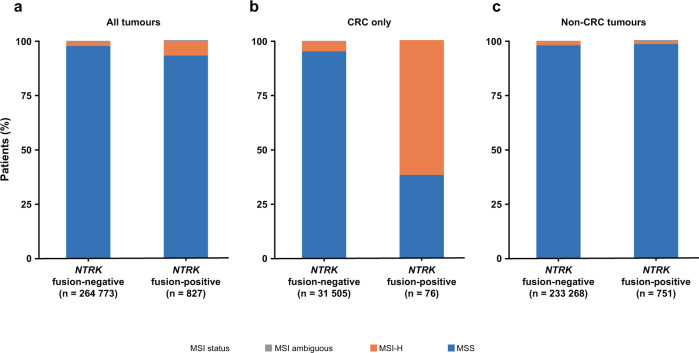
Evaluation of and microsatellite instability (MSI) status in *NTRK* fusion-positive versus *NTRK* fusion-negative solid tumours. MSI status in all tumours (**a**), CRC only (**b**) and non-CRC tumours (**c**). CRC colorectal cancer, MSI-H microsatellite instability-high, MSS microsatellite stable, *NTRK* neurotrophic tyrosine receptor kinase.

We investigated the association of MSI status and *NTRK* gene fusions with a focus on CRC, due to previous reports that spontaneous MSI in CRC enriches for complex genomic rearrangements, including *NTRK* fusions^14^. In *NTRK* fusion-positive CRC, 61.8% of cases were MSI-H (*n* = 47/76). Conversely, few non-CRC *NTRK* fusion-positive (0.93%; *n* = 7/751) or fusion-negative cancers were also MSI-H (1.3%; *n* = 3035/233,268; Fig. 4; Supplementary Table 12). Within *NTRK* fusion-positive MSI-H CRC, significant co-occurrence with *RNF*43 alterations and mutual exclusivity with *BRAF*, *KRAS*, *PIK3CA*, *CTNNB1* and *APC* alterations was observed (Supplementary Table 13a). According to our assessment, spontaneous MSI-H (see Methods) represented 70.2% of *NTRK* fusion-positive MSI-H CRC cases (*n* = 33/47; Table 2). In *NTRK* fusion-positive spontaneous MSI-H CRC, mutual exclusivity was identified with *BRAF* alterations (Supplementary Table 13b). There were no germline MSI-H cases and four ambiguous MSI-H cases among MSI-H *NTRK* fusion-positive CRC (Table 2); no significant co-occurrences or exclusivities were seen in ambiguous MSI-H CRC. There was no enrichment in specific fusion partners seen in MSI-H CRC (data not shown).

**Table 2:** Categorisation of MSI-H status among *NTRK* fusion-positive CRC, *NTRK* fusion-negative CRC and *NTRK* fusion-positive non-CRC tumours.

Group	Total MSI-H, *N*	Spontaneous MSI -H, *n* (%)	Ambiguous MSI -H, *n* (%)	Germline MSI -H, *n* (%)	Other^a^ MSI-H, *n* (%)
*NTRK* fusion-positive MSI-H CRC	47	33 (70.2)	4 (8.5)	0	10 (21.3)
*NTRK* fusion-negative MSI-H CRC	1389	618 (44.5)	228 (16.4)	165 (11.88)	378 (27.2)
*NTRK* fusion-positive MSI-H non-CRC	2972	1545 (52.0)	460 (15.5)	194 (6.53)	773 (26.0)

MSI-H status was categorised into spontaneous, ambiguous or germline depending on alterations/short variants in *PMS2*, *MLH1*, *MSH2* or *MSH6* as described in Methods.

CRC colorectal carcinoma, MSI-H microsatellite instability high, *NTRK* neurotrophic tyrosine receptor kinase.

^a^Other MSI-H refers to any MSI-H samples that did not fit the defined criteria in the Methods section.

### Comparison of *NTRK* fusion-positive tumours in FoundationCORE with clinical trials

**Fig. 5:**
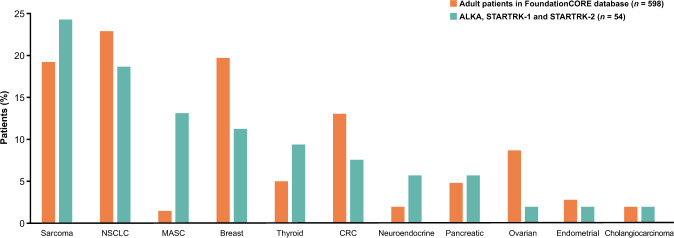
Comparisons of *NTRK* fusion-positive tumour types in entrectinib adult clinical studies versus FoundationCORE database. CRC colorectal cancer, MASC mammary analogue secretory carcinoma, NSCLC non-small cell lung cancer, *NTRK* neurotrophic tyrosine receptor kinase.

Fifty-four adults with 11 different *NTRK* fusion-positive tumour types were enrolled into the entrectinib ALKA-372-001, STARTRK-1 and STARTRK-2 trials^9^. Adult patients from the FoundationCORE database were matched to the 11 *NTRK* fusion-positive disease groups identified in trial patients. The frequency of patients with sarcoma, NSCLC, pancreatic, endometrial and cholangiocarcinoma cancers was similar in the clinical trial population and the FoundationCORE cohort (Fig. 5; Supplementary Table 14). The clinical trial population had a higher frequency of MASC, and the FoundationCORE population had a higher frequency of breast, CRC and ovarian cancers. Median age and sex distributions were similar in patients with *NTRK* fusion-positive tumours from the database and the clinical trials for the 11 matched tumour types (Supplementary Table 15).

## Discussion

We investigated the prevalence of *NTRK* fusion-positive cancers and their relation to other biomarkers in a large population of >295,000 cancer cases from the FoundationCORE database; the largest cohort analysed for *NTRK* fusion-positive cancers to date. Similar to previous studies^4–6^, overall *NTRK* gene fusion prevalence was 0.30% (*n* = 889). Notably, we found a higher prevalence within the paediatric cohort (1.34%) than in adults (0.28%), largely attributed to the different tumour types/histologies commonly identified within these two *NTRK* fusion-positive cohorts. A fusion prevalence around 0.30% with low frequency among common cancers and higher frequency within certain rare cancers is in line with previous estimations from smaller datasets^4–6^. In the FoundationCORE database, Asian ancestry was associated with slightly increased *NTRK* gene fusions prevalence, possibly because of the higher proportion of NSCLC found in this cohort. Generally, *NTRK* gene fusions did not co-occur with other oncogenic drivers, supporting findings from smaller datasets^6^.

Due to the size of the FoundationCORE database and the large cohort of *NTRK* fusion-positive cases analysed, we were able to identify 88 different *NTRK* fusion partner pairs, of which 65.9% had not previously been reported in other large public databases^4–6,8,9,12,13^. Importantly, although the predicted specificity of DNA-based assays is lower than that of RNA-based assays, and thus has greater potential for false-positive results, all of these fusions were predicted to be pathogenic based on conservative definitions for functional rearrangements and mutually exclusive with other known oncogenic drivers, further arguing for their pathogenicity. Moreover, clinical bridging analyses in a selected clinical trial population^15^, estimated a response rate of 72.2% in patients with *NTRK* fusion-positive tumours identified by DNA-based assays, providing more evidence that these assays can detect pathogenic fusions. The significant number of rearrangements identified in our study that were not previously reported highlights the value of analysing large datasets and underscores the need for high-quality diagnostic methods ensuring identification of novel fusion partners. With the rarity of *NTRK* fusions, it seems important to cover known and unknown fusion events to identify patients qualifying for TRK inhibitor treatment. Due to their capacity to detect unknown fusions and to yield lower rates of false-negative and false-positive results than immunohistochemistry^16^, NGS assays are now integral to the process of identifying patients with tumours harbouring an *NTRK* fusion: when testing an unselected population, the ESMO guidelines for *NTRK* testing recommend front-line sequencing or screening by immunohistochemistry followed by sequencing of positive cases^8^.

To assess if the clinical trial cohorts investigating entrectinib for *NTRK* fusion-positive tumours were representative of the real-world situation, we compared the frequencies of the 11 matched *NTRK* fusion-positive tumour types between the clinical trial and real-world populations and found similar frequencies for most cancers. Notably, MASC tumours were much more frequent in the clinical trial population versus the real-world population, likely representing screening biases as *NTRK* fusions are highly prevalent in MASC^7^.

The large dataset analysed here allowed us to further describe the genomic landscape of *NTRK* fusion-positive cancers. In line with the assumption that *NTRK*-fusion-driven cancers are largely devoid of other oncogenic drivers, *NTRK* gene fusions were less likely to co-occur with common drivers, such as those involved in MAPK and PI3K signalling pathways (*KRAS*, *PIK3CA*) and with known oncogenic driver genes in breast cancer, CRC and NSCLC. Consequently, co-occurrence was seen with only 14 genes, including the most common fusion partner. The recent Rosen et al. study in *NTRK* fusion-positive cancers reported no co-occurrence with *KRAS*, *NRAS*, *BRAF*, *EGFR*, *ALK*, *MET* or *ROS1*^6^, and we did not observe co-occurrence with these genes either. Apart from CRC (owing to the over-representation of MSI-H CRC), median TMB was not different between *NTRK* fusion-positive and -negative cases.

It has been described before that spontaneous MSI-H CRC enriches for complex rearrangements and targetable fusions, including *NTRK* fusions^14^. In contrast to hereditary MSI-H CRC (hereditary non-polyposis colorectal cancer [HNPCC]/Lynch syndrome) in the setting of germline mutations in mismatch repair (MMR) genes^17^, spontaneous MSI-H CRC is predominantly caused by methylation of the MLH1 promoter and consecutive inactivation of the *MLH1* gene^18,19^. In up to 75% of spontaneous MSI-H CRC, *BRAF* V600E mutations cause the CpG island methylator phenotype leading to the MLH1 promoter methylation described. Here we show that *NTRK* fusion-positive MSI-H CRC is a unique subset of CRC. First, most *NTRK* fusion-positive CRC cases are MSI-H and can be classified as spontaneous MSI-H. Secondly, and contrary to classical spontaneous MSI-H CRC, *NTRK* fusion-positive spontaneous MSI-H CRC does not carry *BRAF* mutations. This mutual exclusivity with *BRAF* V600E mutations suggests a yet unappreciated very rare subtype of spontaneous MSI-H CRC defined by the presence of *NTRK* gene fusions. Future studies will need to investigate the underlying biology of this observation. Importantly, these findings have immediate clinical implications, as testing for *NTRK* gene fusions in spontaneous MSI-H and *BRAF* wild-type CRC cases could identify patients who may benefit from *NTRK*-directed therapies.

Our study has some limitations. NGS testing with Foundation Medicine Inc assays does not cover the whole exome/genome, so while *NTRK1/2/3* are interrogated, including all exons and specific introns, the description of the genomic characteristics of *NTRK* fusion-positive cancers cannot be considered exhaustive. Furthermore, comparisons to clinical trial cohorts were limited by a lack of clinical and demographic information available in the FoundationCORE database^20^. Finally, as we did not collect clinical outcomes data, we are unable to investigate the prognostic value of *NTRK* fusions in our cohort. Despite this, our study included the largest population used to profile the characteristics and genomic landscape of *NTRK* fusion-positive cancers in a tumour-agnostic setting.

The FoundationCORE database of >295,000 patient records, with an overall prevalence of 0.30% for *NTRK* fusion-positive cancers, allowed us to describe the largest cohort of *NTRK* fusion-positive cancers to date. From these 889 cases, we were able to identify 88 unique fusion partners of which two-thirds had not been reported before, underscoring the critical need for appropriate testing to identify this very small subgroup of cancers. Importantly, we were able to describe a subtype of spontaneous MSI-H CRC defined by the presence of *NTRK* fusions and the absence of otherwise pathogenic *BRAF* V600E mutations. The results presented here deepen our general understanding of *NTRK* fusion-positive cancers and might help clinicians to identify patients potentially suitable for *NTRK*-directed therapies.

## Methods

### FoundationCORE database samples

Comprehensive genomic profiling (CGP), including TMB^21^ and genetic ancestry prediction^22^, were carried out in a Clinical Laboratory Improvement Amendments certified, College of American Pathologists accredited laboratory (Foundation Medicine Inc., Cambridge, MA, USA). Data from 295,676 de-identified, consented-for-research cases between January 2013 and December 2019 from 75 different solid tumour types were profiled. Detailed methods for this assay were previously described by Chmielecki, et al.^23^. Briefly, haematoxylin-and-eosin-stained slides or Wright-Giemsa stained blood/aspirate smears were used to confirm the pathologic diagnosis of each case. Samples containing a minimum of 20% tumour cells were selected for subsequent RNA and/or DNA extraction, from 10-μm formalin-fixed paraffin-embedded (FFPE) sections or fresh blood/bone marrow aspirates, and genomic analysis. The FoundationOne^®^ assay uses adaptor ligation and hybrid capture to analyse DNA for all coding exons of cancer-related genes (v1: *n* = 182; v2: *n* = 287; v3: *n* = 323; v5: *n* = 395) plus select introns from genes frequently rearranged in cancer (v1: *n* = 14; v2: *n* = 19; v3: *n* = 24; v5: *n* = 31)^23^. FoundationOne CDx uses hybrid capture to analyse all coding exons of 309 cancer-related genes plus select introns from 36 genes frequently rearranged in cancer^24^. FoundationOne^®^ Heme v4 (Foundation Medicine Inc., Cambridge, MA, USA) uses DNA- and RNA-based hybrid capture to evaluate all coding exons of 465 genes plus select introns from 31 genes frequently rearranged in cancer; rearrangement analysis in 333 genes was performed by targeted RNA-sequencing for samples that had RNA available^25,26^. The sequences of captured libraries (median exon coverage depth >600x using Illumina, San Diego, CA, USA) were analysed for select gene fusions, indels, base substitutions and copy number alterations, as previously described^25,26^. Variants removed from the dataset included germline variants (1000 Genomes Project [dbSNP142] or dbSNP database http://www.ncbi.nlm.nih.gov/SNP/), those with ≥2 counts in the ExAC database (http://exac.broadinstitute.org/) except for known cancer drivers (e.g. *BRCA1/2* and *TP53* mutations), and recurrent variants of unknown significance predicted to be germline by an internally developed algorithm^27^. Known confirmed somatic alterations according to the Catalogue of Somatic Mutations in Cancer (COSMIC) were highlighted as biologically significant.

Approval was obtained from the Western Institutional Review Board (Protocol No. 20152817). Written consent was obtained to use the de-identified patient samples for research.

### NGS from the FoundationCORE database

Co-occurrence of *NTRK1/2/3* gene fusions with known and likely somatic alterations in each of >300 cancer-related genes was assessed across all samples. Odds ratios for mutational co-occurrence were generated using two-sided Fisher’s exact test. False discovery rate (FDR)-adjusted *P*-values calculated using the Benjamini–Hochberg correction were used to determine significance (*P* < 0.05). Co-occurrence was also evaluated with alterations of disease-specific driver genesets in their respective indications.

Throughout this analysis, *NTRK* fusion-positive cases were defined as those harbouring any *NTRK1/2/3* rearrangement known or suspected to result in a fusion protein, consistent with definitions used in other pan-solid tumour prevalence studies^4–6^.

An assessment of *NTRK* fusion detection rate was conducted for the FoundationOne CDx platform using COSMIC v92 (cancer.sanger.ac.uk) as the reference baseline. Using 10 internal process-matched normal control specimens (each an equal mixture of 10 diploid HapMap cell lines), the mean sequence coverage at all genomic loci was calculated. Conservatively, using 100x as the minimum for which custom FoundationOne CDx algorithms would detect breakpoints, all annotated fusion breakpoints in *NTRK1/2/3* in the COSMIC v92 database (*TPM3:NTRK1*, *TPR:NTRK1* variant I, *TPR:NTRK1* variant II, *TFG:NTRK1*, *LMNA:NTRK1*, *TP53:NTRK1*, *QKI:NTRK2*, *NACC2:NTRK2*, *ETV6:NTRK3* variant I, *ETV6:NTRK3* variant II, *ETV6:NTRK3* variant III, and *ETV6:NTRK3* variant IV) were compared with the empirically measured coverage profile of FoundationOne CDx. The expected *NTRK* fusion detection rate per fusion variant was calculated using Eq. (1):

$$\begin{array}{l}{\mathrm{Fusion}}\,{\mathrm{variant}}\,{\mathrm{count}}\,{\mathrm{in}}\,{\mathrm{COSMIC}}\,\, \times \\ \left[ {\left( {{\mathrm{number}}\,{\mathrm{of}}\,{\mathrm{bps}}\,{\mathrm{in}}\,{\mathrm{breakpoint}}\,{\mathrm{region}}\,{\mathrm{with}} \ge 100{\mathrm{x}}\,{\mathrm{coverage}}\,{\mathrm{in}}\,{\mathrm{FoundationOne}}\,{\mathrm{CDx}}} \right)/\left( {{\mathrm{number}}\,{\mathrm{of}}\,{\mathrm{bps}}\,{\mathrm{in}}\,{\mathrm{breakpoint}}\,{\mathrm{region}}} \right)} \right]\end{array}$$

Fusions without annotated breakpoint regions in COSMIC (*n* = 10) were ignored. All intron and exon base pair counts were measured using the hg19 reference sequence.

### Predicted ancestry

Inferred estimated population ancestry was performed using germline single nucleotide polymorphisms (SNPs). Samples from the 1000 Genomes Project phase III dataset^28^ were used to train a classifier to recognise five ancestral populations: African, Central/South American, East and South Asian and European. In this approach, SNP allele counts variation was captured by the top five principal components^29^, and a random forest classifier was trained to recognise populations based on these four variation measures. The classifier was applied on patient samples to make ancestry calls, with confusion between Central/South American and European ancestries being observed^22,30,31^. Prevalence comparisons between predicted ancestry groups were calculated using two-sided Fisher’s exact test for the group of interest versus all other samples.

### Microsatellite instability

Microsatellite instability-high (MSI-H) status can result from germline mutations in *MMR* genes (HNPCC/Lynch syndrome) or can be spontaneous due to hypermethylation of the *MLH1* gene promoter^20^. Colorectal cancer (CRC) MSI-H status was categorised as spontaneous, germline or ambiguous, based on Sato and colleagues^14^. Spontaneous was defined as absence of known/likely pathogenic alterations (somatic or germline) in *PMS2*, *MLH1*, *MSH2* or *MSH6*^18,19^. Germline was defined as presence of ≥1 known/likely pathogenic variant in *PMS2*, *MLH1*, *MSH2* or *MSH6* with predicted germline status based on a previously described somatic germline zygosity algorithm reported to have a 95–99% accuracy^17,27^. Ambiguous was defined as presence of known/likely pathogenic variant in *PMS2*, *MLH1*, *MSH2* or *MSH6* that had an ambiguous somatic/germline status and no known/likely pathogenic variants in the aforementioned genes with a predicted germline status.

### Clinical trial data comparisons

Details of the three entrectinib phase I/II clinical trials (ALKA-372-001, STARTRK-1, STARTRK-2) have been previously published^9^. In brief, patients were ≥18 years old, with metastatic/locally advanced *NTRK* fusion-positive solid tumours, measurable disease by Response Evaluation Criteria in Solid Tumours v1.1 and Eastern Cooperative Oncology Group performance status ≤2. Patients were enrolled based on local molecular testing (including fluorescence in situ hybridisation, quantitative polymerase chain reaction or DNA/RNA-based NGS) or central RNA-based NGS (Trailblaze Pharos™). Clinical characteristics of patients enrolled into these three clinical trials were compared with those of the real-world population from the FoundationCORE database.

### Reporting summary

Further information on research design is available in the Nature Research Reporting Summary linked to this article.

## Supplementary information

Supplementary Information

Reporting Summary

## Data Availability

The data generated and analysed during this study are described in the following data record: 10.6084/m9.figshare.14604465^32^. The data were generated and analysed under the auspices of Roche, which is a member of the Vivli Center for global clinical research data. Data access conditions are described at https://vivli.org/ourmember/roche/. To request access to individual patient-level data from the clinical trials, first locate the clinical trial in Vivli (https://search.vivli.org/ requires sign up and log in) using the trial registration number (given above), then click the ‘Request Study’ button and follow the instructions. In the event that you cannot see a specific study in the Roche list, an Enquiry Form can be submitted to confirm the availability of the specific study. To request access to individual patient-level data from the clinical trials, first locate the clinical trial in Vivli (https://search.vivli.org/ requires sign up and log in) using the trial registration number (ALKA-372-001 [EudraCT 2012-000148-88], STARTRK-1 [NCT02097810], STARTRK-2 [NCT02568267]), then click the ‘Request Study’ button and follow the instructions. In the event that you cannot see a specific study in the Roche list, an Enquiry Form can be submitted to confirm the availability of the specific study. To request access to related clinical study documents (e.g.: protocols, CSR, safety reports), please use Roche’s Clinical study documents request form: https://www.roche.com/research_and_development/who_we_are_how_we_work/research_and_clinical_trials/our_commitment_to_data_sharing/clinical_study_documents_request_form.htm. Patient-level data which were derived from the Foundation Research dataset and used in the related study cannot be shared as they contain patient genomic information that, depending on the prevalence of the identified alterations, could be used to identify individuals. To maximise transparency and provide the most thorough information without compromising patients’ personal information, the authors have created a large number of supplementary files and made them openly available as part of the figshare data record^32^.
