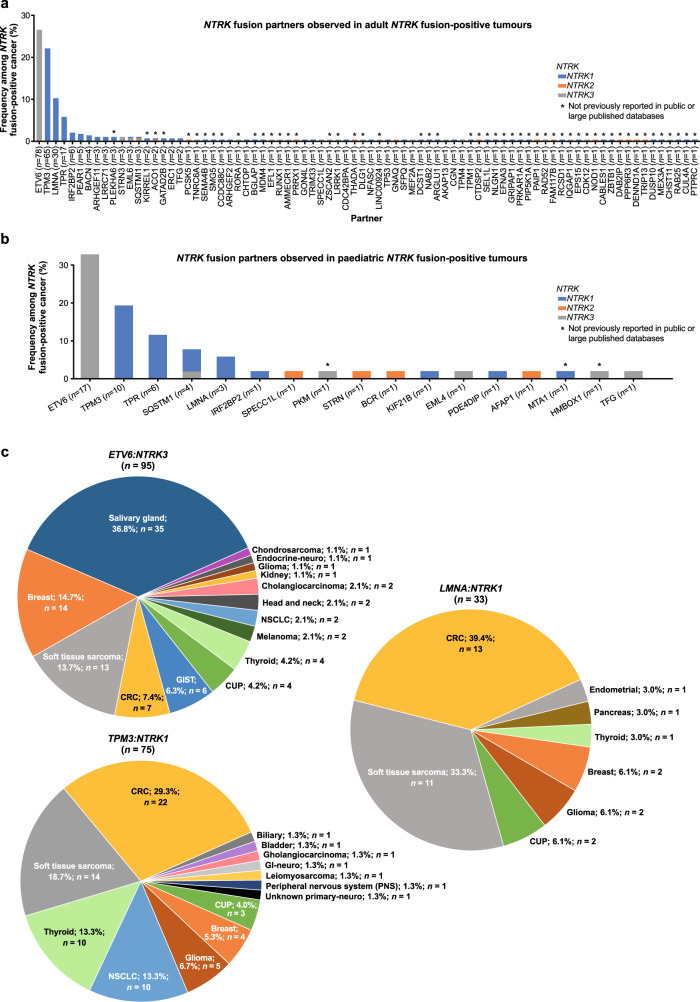# Author Correction: Genomic context of *NTRK1/2/3* fusion-positive tumours from a large real-world population

**DOI:** 10.1038/s41698-021-00222-y

**Published:** 2021-09-17

**Authors:** C. B. Westphalen, M. G. Krebs, C. Le Tourneau, E. S. Sokol, S. L. Maund, T. R. Wilson, D. X. Jin, J. Y. Newberg, D. Fabrizio, L. Veronese, M. Thomas, F. de Braud

**Affiliations:** 1 Comprehensive Cancer Center Munich & Department of Medicine III, University Hospital, LMU Munich, Munich, Germany; 2 Division of Cancer Sciences, Faculty of Biology, Medicine and Health, The University of Manchester and The Christie NHS Foundation Trust, Manchester Academic Health Science Centre, Manchester, UK; 3 Department of Drug Development and Innovation (D3i), Institut Curie, Paris & Saint-Cloud, France, Saint-Cloud, France; 4 INSERM U900 Research Unit, Saint-Cloud, France; 5 Paris-Saclay University, Paris, France; 6 Foundation Medicine Inc., Cambridge, MA USA; 7 Genentech Inc., South San Francisco, CA USA; 8 F. Hoffmann-La Roche Ltd, Basel, Switzerland; 9 Department of Medical Oncology and Haematology, Fondazione IRCCS Istituto Nazionale dei Tumori, Milan, Italy; 10 School of Specialisation in Medical Oncology, University of Milan, Milan, Italy

**Keywords:** Cancer, Oncogenes

Correction to: *npj Precision Oncology* 10.1038/s41698-021-00206-y, published online 20 July 2021

The original version of this Article contained a typographical error in the pie chart label of Figure 2c. This has now been corrected in the PDF and HTML versions of the Article.