# Supplementary material for: Genomic context of NTRK1/2/3 fusion-positive tumours from a large real-world population
Source: NPJ Precis Oncol. 2021 Jul 20;5:69. doi: 10.1038/s41698-021-00206-y (PMC8292342; doi:10.1038/s41698-021-00206-y)

Supplementary Materials

Supplementary Table 1. Individual patient demographics of patients with *NTRK* fusion-positive tumours in FoundationCORE **2**

Supplementary Table 2. Prevalence of *NTRK* fusion-positive specimens in FoundationCORE by tumour histology **17**

Supplementary Table 3. Prevalence of *NTRK* fusion-positive specimens in FoundationCORE by age group **34**

Supplementary Table 4. Prevalence of *NTRK* fusion-positive specimens by disease ontology in paediatric age groups **35**

Supplementary Table 5. Expected *NTRK* fusion detection rates on FoundationOne CDx . **36**

Supplementary Table 6. *NTRK* gene fusions detected among adult patients **37**

Supplementary Table 7. *NTRK* gene fusions detected among paediatric patients **41**

Supplementary Table 8. Disease group breakdown of all specimens and *NTRK* fusion-positive specimens by predicted ancestry **42**

Supplementary Table 9. Co-occurrence and mutual exclusivity of altered genes with *NTRK* fusions in solid tumours **45**

Supplementary Table 10. Mutations found within driver genes in *NTRK*+ and *NTRK*– colorectal cancer (CRC), breast cancer and non-small cell lung cancer (NSCLC) **49**

Supplementary Table 11. Summary of co-occurrence and mutual exclusivity of driver gene mutations and microsatellite instability high (MSI-H) status with specific *NTRK* fusion-positive cancers **50**

Supplementary Table 12. Evaluation of and microsatellite instability (MSI) status in *NTRK* fusion-positive versus *NTRK* fusion-negative solid tumours **51**

Supplementary Table 13. Genes with significantly co-occurring or mutually exclusive alterations with *NTRK* fusions in all MSI-H CRC (a) and in spontaneous MSI-H CRC (b) **52**

Supplementary Table 14. Comparisons of *NTRK* fusion-positive tumour types in entrectinib adult clinical studies versus FoundationCORE database **53**

Supplementary Table 15. Comparisons of patient demographics in entrectinib adult clinical studies[9] and the FoundationCORE database **54**

Supplementary Figure 1. The prevalence of *NTRK* fusions by predicted genetic ancestry in solid tumours **55**

Supplementary Figure 2. Evaluation of tumour mutational burden (TMB) in *NTRK* fusion-positive versus *NTRK* fusion-negative solid tumours **56**

Supplementary Table 1. Individual patient demographics of patients with *NTRK* fusion-positive tumours in FoundationCORE

de-identified ID	local_met_status	age [0-89] capped at 89	gender
XRN:LXMVBX	met	70	Male
XRN:SVTYCA	local	41	Male
XRN:YVU1G5	met	65	Female
XRN:W8BBMB	local	8	Female
XRN:AYXH86	unknown	84	Female
XRN:7MSN3W	unknown	63	Female
XRN:P7SAMG	met	17	Male
XRN:8Y2JC2	unknown	68	Female
XRN:QDK92R	unknown	48	Female
XRN:VFAF4X	unknown	67	Female
XRN:BHCD2F	local	50	Female
XRN:NMNLHF	local	61	Female
XRN:9685FW	met	69	Male
XRN:W0AXW9	met	49	Female
XRN:1YUXU5	local	80	Male
XRN:QLF0N8	unknown	63	Male
XRN:KWCX4T	local	67	Male
XRN:29YSFR	local	69	Male
XRN:T9UQQ8	ln	38	Male
XRN:E4F1QW	unknown	61	Female
XRN:BC2KFL	unknown	1	Female
XRN:1R57K0	met	82	Female
XRN:5BU58K	unknown	2	Male
XRN:KV27L1	local	68	Male
XRN:AU9QRD	unknown	75	Male
XRN:QFGM8U	local	70	Female
XRN:MPPMPA	local	52	Male
XRN:QFUESF	met	72	Female
XRN:SAQRSL	unknown	45	Male
XRN:LK7BEH	local	65	Male
XRN:UE5FDE	unknown	42	Male
XRN:13YXPD	met	80	Male
XRN:93JWYR	local	70	Male
XRN:WBY6RS	local	77	Male
XRN:5T32GR	local	70	Female
XRN:SSRA6V	met	61	Male
XRN:HH66XV	local	76	Male
XRN:68PV49	ln	45	Female
XRN:LDP6F6	local	56	Female
XRN:JFLAVS	unknown	75	Female
XRN:QP1D8C	met	43	Female
XRN:VW5XG4	ln	35	Female
XRN:HSCHFS	local	76	Female
XRN:5KBRPA	met	65	Female
XRN:2FV3SK	met	70	Female
XRN:MW1LSF	unknown	55	Male
XRN:LXS59X	unknown	75	Female
XRN:XLQXN3	unknown	40	Female
XRN:85DPPC	unknown	68	Female
XRN:FM1D1V	local	80	Female
XRN:L9ANGC	met	68	Male
XRN:WQXYW7	met	71	Male
XRN:WJ0CPC	met	76	Female
XRN:5T14RQ	met	62	Male
XRN:91LMQF	met	67	Female
XRN:C6BAUP	met	77	Female
XRN:2U792H	met	76	Male
XRN:5CMYPV	unknown	72	Female

de-identified ID	local_met_status	age [0-89] capped at 89	gender
XRN:4X3C2S	local	75	Female
XRN:J65S4C	local	40	Female
XRN:VBT1GD	unknown		Female
XRN:KVPTXJ	unknown	42	Male
XRN:6SH7PL	unknown	55	Male
XRN:16UR56	ln	56	Female
XRN:QQXUME	local	73	Female
XRN:Q2ADSW	met	80	Female
XRN:LSRM9E	local	45	Male
XRN:46BR2T	local	53	Female
XRN:GU1LJR	met	52	Female
XRN:738HCF	local	76	Male
XRN:9RXAUP	local	62	Male
XRN:9H0RWQ	local	69	Female
XRN:N4LGNU	ln	15	Female
XRN:U38LBS	met	65	Female
XRN:26P21K	unknown	60	Female
XRN:7Y0T6Y	unknown	69	Female
XRN:0NHNHD	met	69	Male
XRN:4PU5CD	met	46	Female
XRN:JWEG32	ln	55	Female
XRN:74LDVE	local	68	Female
XRN:FPACLA	unknown	32	Male
XRN:XN5167	local	66	Male
XRN:BFY8QD	unknown	59	Male
XRN:1CEBJN	met	42	Female
XRN:B724F1	unknown	52	Female
XRN:1CPBT7	local	11	Male
XRN:DR2YQ2	unknown	67	Female
XRN:SYGYCT	met	70	Female
XRN:K98T1E	met	56	Female
XRN:XGUUGK	met	65	Female
XRN:FTJ7VG	local	72	Male
XRN:GJVUPV	unknown	48	Female
XRN:VTH671	local	64	Female
XRN:M7M9DL	unknown	52	Female
XRN:UBRJSQ	unknown	45	Female
XRN:DG7LNT	ln	87	Female
XRN:MSKV4V	local	78	Male
XRN:9H0EWG	met	65	Male
XRN:UUGPPM	met	73	Female
XRN:6MC3B3	unknown	70	Female
XRN:TBQ8K6	local	57	Female
XRN:L6E0XT	unknown	72	Female
XRN:F46PJD	unknown	33	Female
XRN:LF8NA6	local	34	Female
XRN:3GHRC4	local	57	Female
XRN:68WJ26	unknown	4	Male
XRN:52EQSH	local	69	Female
XRN:8GTU5F	local	26	Female
XRN:DUQSGV	unknown	7	Male
XRN:7YGW3G	local	37	Female
XRN:JC3QBQ	local	33	Male
XRN:LYQ3YM	unknown	57	Female
XRN:GRNRJ0	local	56	Female
XRN:PMVE30	unknown	63	Male
XRN:5D85CF	local	56	Male
XRN:CB9K4D	local	27	Female
XRN:XTYJF9	local	76	Female
XRN:18WJ9M	met	61	Female

de-identified ID	local_met_status	age [0-89] capped at 89	gender
XRN:2G4SQU	unknown	48	Female
XRN:WA20SU	local	66	Female
XRN:VCTTSV	ln	59	Male
XRN:B0WBG9	unknown	68	Female
XRN:56PH29	ln	45	Male
XRN:03068E	local	79	Male
XRN:RTNV0K	local	38	Male
XRN:79H5K8	met	40	Female
XRN:9U5M28	local	73	Female
XRN:P88XPL	local	76	Female
XRN:18YEAU	unknown	58	Female
XRN:SMAWBM	ln	65	Female
XRN:BKNM23	unknown	54	Female
XRN:CKCXS5	met	56	Male
XRN:9X4AT2	unknown	78	Female
XRN:Y4KRYT	local	62	Female
XRN:9KNHKN	met	65	Female
XRN:46JV7T	local	40	Male
XRN:WPJKM6	unknown	83	Female
XRN:72PMV5	unknown	71	Male
XRN:78UVMY	local	43	Male
XRN:PAUP4D	local	59	Female
XRN:EAY8VA	local	68	Female
XRN:KLQPEC	local	71	Female
XRN:CXMDSV	met	53	Male
XRN:8WWK4W	met	67	Female
XRN:X859WX	ln	67	Female
XRN:8DMFJN	local	6	Female
XRN:AGQ1K0	local	56	Female
XRN:V81YJD	unknown	55	Female
XRN:T84Q9U	met	64	Female
XRN:HQBFJY	unknown	53	Female
XRN:55G7YM	local	74	Female
XRN:SBJ5V5	met	69	Male
XRN:2GN22H	unknown	75	Female
XRN:QRSBJC	unknown	82	Male
XRN:GJ627E	local	58	Male
XRN:K1BRNA	local	50	Male
XRN:TM7TYF	met	55	Female
XRN:N038X4	ln	70	Male
XRN:4W2LPA	unknown	50	Male
XRN:NV3QDR	unknown	22	Male
XRN:REXVUC	local	35	Female
XRN:7GGD68	local	47	Female
XRN:UN4MB7	unknown	0	Male
XRN:9P19GW	met	31	Male
XRN:UHE4AN	local	45	Male
XRN:KHAJKG	met	62	Female
XRN:8MTU70	local	50	Female
XRN:5JJPLQ	unknown	78	Female
XRN:NQRF68	unknown	64	Male
XRN:DSUACJ	ln	71	Male
XRN:JUP6G6	unknown	5	Male
XRN:JY4GB0	met	74	Female
XRN:EPAPR0	met	61	Male
XRN:TM9UPK	unknown	66	Female
XRN:N372SK	local	67	Female
XRN:VN4F0P	met	65	Male
XRN:F8UVQG	met	68	Female
XRN:DJEVES	unknown	53	Male

de-identified ID	local_met_status	age [0-89] capped at 89	gender
XRN:MC45LJ	unknown	54	Male
XRN:P6AKTG	met	75	Female
XRN:9XBFQ0	local	78	Female
XRN:HBNV18	local	22	Female
XRN:GY00AN	met	66	Female
XRN:URADLC	unknown	2	Male
XRN:9HSYN1	local	10	Male
XRN:7QC87W	unknown	66	Female
XRN:8B86TQ	unknown	61	Female
XRN:X3H91S	local	47	Female
XRN:TMDCPN	unknown	61	Male
XRN:Y8XTL1	local	81	Male
XRN:NRJ5FL	ln	74	Male
XRN:UVUJPK	met	64	Male
XRN:5LDGEE	unknown	57	Male
XRN:BFEQQ5	unknown	2	Female
XRN:N3SGXG	local	52	Male
XRN:LKD4PF	local	19	Male
XRN:G0U5NF	met	61	Female
XRN:VVFH9A	met	69	Male
XRN:AK05K6	local	43	Female
XRN:HE5V2C	local	44	Male
XRN:FSBJ3G	local	65	Female
XRN:XNDLA8	local	55	Male
XRN:3MJX5J	local	65	Male
XRN:9NCR27	local	36	Female
XRN:RY19S3	met	66	Male
XRN:THAAQP	local	39	Male
XRN:1L4DQD	local	16	Male
XRN:JAGJG6	unknown	60	Female
XRN:S49FUX	local	82	Male
XRN:QBHLNS	ln	70	Male
XRN:RPVA3X	local	57	Female
XRN:GFT12G	ln	68	Female
XRN:QRT6TW	local	62	Male
XRN:TQPFA1	unknown	84	Female
XRN:JAKFNT	ln	60	Male
XRN:GGSF4W	unknown	65	Female
XRN:6Y6E15	local	73	Female
XRN:0KDX6M	local	54	Male
XRN:6EVXXH	met	42	Female
XRN:E593Q7	met	68	Female
XRN:HG6896	local	49	Female
XRN:E1ENPW	met	66	Female
XRN:1634N8	met	58	Male
XRN:GLAB3G	met	66	Male
XRN:DA6HVX	met	63	Male
XRN:XQU1J2	ln	68	Female
XRN:9U46L0	local	13	Male
XRN:G0KM26	unknown	0	Female
XRN:T7CBLA	local	44	Female
XRN:UHT59G	ln	55	Female
XRN:F49PV0	local	68	Male
XRN:X0A3QH	met	76	Female
XRN:RRRAGC	ln	39	Female
XRN:HNPRFG	local	68	Male
XRN:T3W3T4	met	70	Male
XRN:Q1QJJM	local	71	Male
XRN:GVAAR0	met	66	Female
XRN:QK19BF	ln	64	Female

de-identified ID	local_met_status	age [0-89] capped at 89	gender
XRN:DQUJXV	unknown	37	Female
XRN:KXW3VU	unknown	48	Female
XRN:GTM7U5	met	45	Female
XRN:MC0L5P	local	69	Female
XRN:Q0NEAP	local	59	Female
XRN:3N4XAF	unknown	41	Male
XRN:GUHD4J	local	56	Male
XRN:2EX326	local	80	Male
XRN:D5XFWD	met	52	Female
XRN:Y6AEUF	local	63	Female
XRN:CGUV4N	unknown	0	Male
XRN:M4EVXG	local	74	Male
XRN:C7N776	local	59	Male
XRN:H8TWS5	met	59	Male
XRN:1SJABA	ln	74	Female
XRN:17SBXS	met	54	Female
XRN:NLVT1G	unknown	78	Male
XRN:FJ15MA	ln	24	Female
XRN:UPCMD4	met	62	Female
XRN:H8PPVL	local	71	Female
XRN:8K4CHL	unknown	65	Female
XRN:6FHSLN	unknown	48	Female
XRN:3AG6JV	unknown	52	Female
XRN:8PFVJK	ln	45	Female
XRN:8XETE1	met	57	Female
XRN:R6XMMX	local	84	Male
XRN:FDXYN6	unknown	53	Male
XRN:WSVAPY	met	66	Male
XRN:53AQSA	met	55	Female
XRN:BKKN5C	local	36	Female
XRN:77N67T	met	64	Female
XRN:F068ML	local	43	Female
XRN:PEDH9X	unknown	27	Male
XRN:YR826X	unknown	67	Female
XRN:WTQ2GL	unknown	7	Male
XRN:8FXHGK	local	69	Female
XRN:WXEF89	met	54	Male
XRN:VKLN7D	local	71	Female
XRN:WGHJ3N	ln	61	Male
XRN:5AVD8S	unknown	72	Male
XRN:TM2HPG	local	60	Female
XRN:5AG3LD	unknown	66	Male
XRN:2CR4L7	local	71	Female
XRN:REQW0G	local	62	Female
XRN:D50D8Y	ln	81	Male
XRN:A837P8	local	49	Female
XRN:0UVF10	unknown	58	Male
XRN:GC7LD7	local	65	Female
XRN:0LLSE7	met	72	Female
XRN:NPL3W2	local	1	Male
XRN:J62B6W	unknown	30	Female
XRN:CDRHY5	met	53	Female
XRN:612VL5	unknown	47	Male
XRN:7EWJJ2	local	58	Male
XRN:DS6HTV	local	74	Male
XRN:BFCEJK	ln	55	Female
XRN:59T07G	local	52	Female
XRN:5RVGQD	unknown	58	Female
XRN:YNSKAY	local	37	Female
XRN:2PVSC7	local	55	Female

de-identified ID	local_met_status	age [0-89] capped at 89	gender
XRN:QPHLPN	local	85	Male
XRN:2VN2LY	local	72	Male
XRN:QEK6SM	met	47	Male
XRN:E00UP9	local	52	Female
XRN:439WQB	local	61	Female
XRN:NU4H4C	local	59	Male
XRN:QPMMRG	local	40	Male
XRN:QJ8FJ0	local	62	Male
XRN:QADMF1	met	65	Male
XRN:EYXMLE	local	61	Male
XRN:EPXJ7P	unknown	58	Female
XRN:MWHA17	unknown	11	Female
XRN:HR63F8	local	77	Female
XRN:QF0FAK	local	27	Male
XRN:LNFW0F	local	72	Female
XRN:5BMQVF	unknown	9	Male
XRN:Y3R7C9	local	65	Female
XRN:M64KJT	ln	61	Female
XRN:UMLQH2	met	64	Female
XRN:BXNJS3	local	14	Female
XRN:FQJHLL	met	59	Male
XRN:B6BPHC	unknown	76	Male
XRN:21DYBR	local	3	Female
XRN:LDDF0L	ln	62	Female
XRN:X504BC	local	69	Female
XRN:UK37C4	local	8	Female
XRN:MG03YL	ln	32	Male
XRN:MBHADL	local	55	Female
XRN:W7JXRU	local	64	Male
XRN:93WJ72	unknown	55	Male
XRN:CQ7DS1	unknown	60	Female
XRN:AMVE4D	met	61	Female
XRN:THRB3E	ln	66	Male
XRN:NEDBYW	local	1	Male
XRN:6JL94F	met	62	Female
XRN:2D8KWE	met	74	Female
XRN:LYWSFA	unknown	59	Female
XRN:BKP9VX	unknown	41	Male
XRN:L2C9E4	local	82	Male
XRN:CSK35X	local	65	Male
XRN:D04QM8	met	71	Male
XRN:EBSMKH	unknown	83	Male
XRN:HUY8H1	met	46	Female
XRN:P5BSYQ	local	77	Male
XRN:SM992B	unknown	83	Male
XRN:GSAG4K	local	75	Male
XRN:M064W5	met	45	Female
XRN:F9Y5YL	local	32	Female
XRN:HVQYMW	local	58	Female
XRN:560X9S	ln	65	Male
XRN:N7EKCG	ln	71	Female
XRN:4KGWN4	met	80	Female
XRN:CFEJ4L	met	65	Female
XRN:4G1NVG	local	55	Female
XRN:7HTE1B	local	68	Male
XRN:ARA9QH	unknown	41	Female
XRN:D3G0LF	unknown	52	Female
XRN:70YL0T	unknown	54	Female
XRN:9QHCNQ	local	2	Female
XRN:PT57PT	unknown	78	Male

de-identified ID	local_met_status	age [0-89] capped at 89	gender
XRN:DPD1KA	local	63	Female
XRN:GVM4BM	unknown	72	Female
XRN:6AUSLN	met	61	Female
XRN:1Q4J8B	local	35	Female
XRN:SL36LD	ln	70	Male
XRN:EFBVY2	met	57	Female
XRN:EJXJNA	local	69	Male
XRN:4X087S	local	35	Female
XRN:1QYNS8	local	79	Female
XRN:T07TY9	unknown	4	Female
XRN:KVXBJ9	local	0	Male
XRN:H4N052	local	36	Female
XRN:4J6CG8	met	83	Female
XRN:KEUM7H	local	52	Male
XRN:MGW1PC	met	81	Female
XRN:RVEU5R	unknown	89	Male
XRN:8QF8S0	unknown	14	Female
XRN:4X4H0L	ln	65	Female
XRN:XE3FX2	local	54	Female
XRN:5B1Q4P	unknown	36	Female
XRN:SHN4R6	local	72	Male
XRN:30N4JG	local	58	Female
XRN:FBDHP8	local	68	Female
XRN:6PJQXB	met	25	Female
XRN:HK1AQ4	met	51	Female
XRN:04TPB7	met	57	Male
XRN:4QE3QK	local	78	Male
XRN:YMUHBW	local	55	Female
XRN:344H9E	local	0	Female
XRN:SAEARH	local	72	Male
XRN:AJBBL5	local	38	Female
XRN:VU1KB4	unknown	84	Male
XRN:719D9M	met	50	Female
XRN:GNNWSY	met	74	Male
XRN:3BMAXY	ln	70	Female
XRN:0UN8PA	met	66	Male
XRN:4BXXYV	local	74	Male
XRN:AS5TD2	unknown	54	Male
XRN:8DDH0L	local	10	Female
XRN:R2D27R	met	69	Female
XRN:XYCJ93	local	56	Female
XRN:4FES0X	local	66	Female
XRN:PRTESJ	local	65	Male
XRN:SMLE5L	unknown	32	Male
XRN:84MM2F	local	55	Female
XRN:Q9HT48	met	70	Female
XRN:5YTFBB	ln	85	Female
XRN:Q88K42	local	80	Female
XRN:N4MNEE	met	66	Male
XRN:F5JCJY	unknown	78	Male
XRN:P34TSV	met	65	Male
XRN:Q5V97H	local	84	Male
XRN:JW01JL	local	48	Male
XRN:WKPSP4	local	50	Male
XRN:DEBG8E	unknown	45	Female
XRN:K07Y4A	local	60	Female
XRN:U4X4EB	unknown	64	Female
XRN:XJG0YT	met	48	Male
XRN:9KJ2V6	local	31	Male
XRN:N0QXJ9	unknown	82	Male

de-identified ID	local_met_status	age [0-89] capped at 89	gender
XRN:61P73Y	met	64	Male
XRN:ARF0EV	local	78	Female
XRN:HLR0Y9	local	17	Female
XRN:5VP2W1	local	77	Female
XRN:9R3K45	unknown	34	Female
XRN:5H19TB	met	51	Female
XRN:CT55L7	unknown	64	Male
XRN:T7625B	local	54	Female
XRN:UTHG1W	met	72	Female
XRN:9N4G66	met	54	Female
XRN:X6J96S	met	79	Female
XRN:DS5V9T	ln	86	Female
XRN:MJHCKW	local	30	Male
XRN:WAW6RY	local	65	Male
XRN:C8ANJ3	ln	45	Female
XRN:9UBAP8	unknown	59	Male
XRN:FN5L9T	local	59	Male
XRN:K4GMP0	local	45	Male
XRN:K2SE0D	local	61	Female
XRN:WYRWAY	unknown	66	Male
XRN:PUG6ER	unknown	67	Male
XRN:GL9698	unknown	42	Male
XRN:PFX70S	local	86	Male
XRN:LHCFEB	local	44	Male
XRN:YF358T	met	3	Female
XRN:8DQFT3	met	68	Male
XRN:RJPRLH	unknown	3	Female
XRN:47PX7T	met	76	Male
XRN:KV56LY	unknown	1	Male
XRN:9QV6YQ	met	54	Female
XRN:47DUHD	ln	59	Male
XRN:BJ8PSW	local	59	Female
XRN:8C6C6K	met	67	Female
XRN:93GFX5	local	17	Female
XRN:TCN41C	local	36	Male
XRN:J238DC	local	67	Male
XRN:U0R341	local	63	Female
XRN:PT6CAD	met	64	Female
XRN:X9JR9Y	unknown	84	Male
XRN:MN3HH5	met	60	Male
XRN:VFT9XQ	unknown	68	Female
XRN:D1BXXS	ln	72	Female
XRN:AGFGV5	local	73	Female
XRN:SB28UY	local	59	Male
XRN:HK7AJD	unknown	70	Male
XRN:97S14V	unknown	50	Female
XRN:DSWHNM	local	43	Male
XRN:P67N3L	unknown	63	Male
XRN:K5MH6H	unknown	28	Female
XRN:WJWTLL	local	88	Female
XRN:AVXAGN	unknown	18	Male
XRN:CT1VME	met	53	Female
XRN:51B1AE	local	62	Male
XRN:279HYS	local	67	Female
XRN:H2RLXC	local	60	Male
XRN:U2MVNN	local	69	Female
XRN:FSRDXQ	met	61	Male
XRN:VWUQF0	met	29	Female
XRN:NVF1KU	unknown	20	Male
XRN:W7RL06	unknown		Male

de-identified ID	local_met_status	age [0-89] capped at 89	gender
XRN:B3PQVN	local	58	Male
XRN:P68BUA	met	49	Female
XRN:CLF17N	local	71	Male
XRN:QRV9MS	unknown	65	Male
XRN:54TNWJ	met	71	Female
XRN:XSYMNQ	unknown	70	Male
XRN:NPCWVT	local	62	Female
XRN:LD8E0V	local	64	Male
XRN:1KHQ4G	unknown	24	Male
XRN:KKPL11	local	72	Male
XRN:MC2VM3	unknown	32	Male
XRN:6JTAG1	unknown	49	Male
XRN:H78M9H	local	76	Female
XRN:NMY75D	local	58	Male
XRN:AB5FEU	unknown	65	Male
XRN:H9UT06	local	40	Female
XRN:8AHNWE	unknown	71	Male
XRN:NR9J8U	local	32	Female
XRN:94SPA9	ln	63	Female
XRN:GA3VV7	met	69	Male
XRN:8WMDR5	local	65	Male
XRN:UEP4VY	unknown	18	Female
XRN:9VM7TP	local	72	Female
XRN:YQNGKR	met	58	Female
XRN:QVAKVU	local	71	Male
XRN:QPYWD3	unknown	32	Female
XRN:AQR7RQ	local	55	Female
XRN:3P7LDE	local	26	Male
XRN:2F464A	local	72	Male
XRN:GFKALV	met	62	Female
XRN:T2V7AL	met	59	Female
XRN:RQ9N8G	unknown	66	Male
XRN:BYKJCG	unknown	8	Female
XRN:1Q8EY4	local	44	Female
XRN:TCA5AW	met	53	Female
XRN:VJUM0L	local	49	Female
XRN:SV732Y	local	78	Male
XRN:1NH29M	met	47	Female
XRN:UWTNX2	unknown	66	Male
XRN:2CTHBW	local	63	Male
XRN:CGPLWG	unknown	85	Male
XRN:C8PSD2	local	22	Female
XRN:X3AS1B	unknown	60	Female
XRN:7RC7RS	unknown	83	Female
XRN:GJNQD3	local	46	Female
XRN:Y0HRKF	unknown	0	Female
XRN:A91HU3	unknown	81	Female
XRN:45MGQL	unknown	46	Female
XRN:4FC462	unknown	60	Female
XRN:DV3FAP	met	89	Male
XRN:RNSHUE	unknown	65	Female
XRN:NW0X7S	unknown	53	Male
XRN:GJB4F4	ln	66	Male
XRN:7V7AMD	met	60	Female
XRN:G2X5W1	unknown	17	Female
XRN:2AUNEA	met	48	Female
XRN:G9NJ2S	ln	82	Female
XRN:D2QWUS	local	62	Female
XRN:W01KL5	local	86	Male
XRN:D8ECMA	local	69	Female

de-identified ID	local_met_status	age [0-89] capped at 89	gender
XRN:B1946R	ln	89	Female
XRN:0T77MK	local	79	Male
XRN:NS9VLJ	met	67	Female
XRN:QHNLUG	local	58	Female
XRN:WAJ497	unknown	61	Female
XRN:X1HD7V	ln	54	Female
XRN:MV1VQL	ln	47	Female
XRN:F9ARJF	ln	72	Female
XRN:XDJR6Q	unknown	61	Male
XRN:K6VU8W	unknown	70	Male
XRN:FSXN7M	local	77	Male
XRN:9KTB03	unknown	66	Female
XRN:KDRBAJ	unknown	40	Male
XRN:ANHTSS	local	69	Male
XRN:2WEB3P	unknown	74	Female
XRN:K19WRF	local	60	Female
XRN:H845TE	local	56	Female
XRN:EGL2LB	met	48	Male
XRN:B9UTSH	local	1	Male
XRN:MJB98F	ln	63	Female
XRN:JS6SDF	unknown	24	Male
XRN:B1MULM	unknown	78	Male
XRN:AFAEVN	local	71	Female
XRN:N0VLH0	local	58	Male
XRN:90PSCM	unknown	63	Female
XRN:LKQ6FL	local	59	Male
XRN:T6EKXH	local	13	Male
XRN:M5226P	local	74	Female
XRN:TRNUL0	unknown	36	Male
XRN:FQWGN0	ln	55	Female
XRN:8NPDGM	unknown	72	Male
XRN:LQVACQ	local	72	Female
XRN:6LN6HJ	met	39	Female
XRN:7XC31J	unknown	74	Male
XRN:27W9VG	unknown	56	Male
XRN:W4D7YY	met	47	Female
XRN:R1V1DT	ln	69	Female
XRN:SNED2C	ln	81	Female
XRN:VVA95M	ln	49	Female
XRN:42BUW8	local	34	Female
XRN:H0RT2S	unknown	42	Female
XRN:8BU0YN	unknown	67	Male
XRN:SNA3UB	ln	73	Male
XRN:9SJQCP	local	63	Male
XRN:C8QDJG	local	59	Female
XRN:AW746M	local	68	Female
XRN:1RGSQY	unknown	60	Female
XRN:P0N4PR	met	63	Male
XRN:S13XLE	met	52	Female
XRN:36N3DC	unknown	48	Female
XRN:D068YR	local	58	Female
XRN:AJD6E9	met	89	Male
XRN:5R2KTT	local	72	Male
XRN:9818H7	unknown	80	Female
XRN:BU361R	local	59	Female
XRN:S56PCK	local	75	Female
XRN:7SJFU2	met	70	Female
XRN:62EDG3	local	66	Male
XRN:6V9UFN	unknown	1	Female
XRN:KY0TLK	unknown	37	Female

de-identified ID	local_met_status	age [0-89] capped at 89	gender
XRN:REFKGB	unknown	18	Male
XRN:TTYC93	unknown	17	Female
XRN:Q1YX69	met	62	Male
XRN:SJH40Q	local	62	Female
XRN:5VYDS9	met	37	Female
XRN:L0KVAK	met	58	Male
XRN:9F7FQH	met	66	Male
XRN:72TWA6	ln	49	Female
XRN:KE6226	unknown	74	Female
XRN:K88TF8	local	69	Female
XRN:B3U3XQ	local	63	Female
XRN:AN47AM	local	52	Male
XRN:DF0UBM	local	62	Female
XRN:W302LC	local	69	Female
XRN:DMELE2	local	66	Male
XRN:B0GHD3	unknown	36	Male
XRN:JC1720	met	59	Male
XRN:A5XFV8	local	62	Female
XRN:KLBYPJ	local	6	Male
XRN:84QRV9	local	77	Male
XRN:CTM1XF	local	43	Male
XRN:245RH8	local	70	Male
XRN:NBCCNT	local	69	Male
XRN:2A9DCD	ln	60	Female
XRN:DC0EAD	ln	60	Female
XRN:XQRHSE	local	52	Male
XRN:GN40EN	unknown	43	Female
XRN:B7E8A3	unknown	89	Male
XRN:85DEMN	local	66	Male
XRN:13KKF8	unknown	48	Female
XRN:DNMSG9	local	70	Male
XRN:N39959	unknown	70	Female
XRN:90V2L6	met	65	Female
XRN:Y1PPTD	met	60	Female
XRN:DTHMJ6	unknown	23	Male
XRN:JTN1MC	unknown	89	Female
XRN:67DD1N	local	54	Male
XRN:MBNJKH	local	73	Female
XRN:S1584E	ln	15	Female
XRN:R31DLC	ln	72	Male
XRN:4N7LRC	unknown	51	Female
XRN:75MB0H	ln	64	Male
XRN:HMC1PC	unknown	28	Male
XRN:65GVB4	local	52	Female
XRN:MVSKE9	unknown	70	Female
XRN:UY5RRT	local	67	Female
XRN:LP0TYX	local	57	Male
XRN:MXDYRK	local	50	Male
XRN:0C4T4M	ln	57	Female
XRN:4SP0DS	local	68	Male
XRN:9SKXYU	unknown	77	Male
XRN:XMK26L	local	59	Female
XRN:QASEHV	local	64	Male
XRN:4M2KBS	met	70	Female
XRN:NK844L	local	76	Female
XRN:HG7421	unknown		Female
XRN:N2PP2Q	met	62	Female
XRN:GG1DX3	unknown	59	Female
XRN:3ATV2E	met	49	Female
XRN:HLX7R4	unknown	85	Male

de-identified ID	local_met_status	age [0-89] capped at 89	gender
XRN:HJ80MU	local	59	Male
XRN:A1DY0B	unknown	64	Female
XRN:R2U74L	local	51	Female
XRN:A9HGAD	unknown	64	Female
XRN:JW50V5	met	42	Female
XRN:QD3FE3	unknown	24	Female
XRN:B2CQWF	unknown	32	Female
XRN:30NP36	unknown	68	Male
XRN:SC3WHT	met	58	Female
XRN:1EVXY3	met	34	Female
XRN:39M0CM	met	69	Male
XRN:N9MBM9	unknown	71	Female
XRN:9CP4N7	local	78	Male
XRN:GDXQBX	unknown	27	Male
XRN:2FWKA9	unknown	68	Male
XRN:9G3WBF	ln	34	Female
XRN:LD4M6G	unknown	35	Female
XRN:XHEP0D	unknown	59	Male
XRN:LP5XLV	local	84	Female
XRN:KNHFTY	met	63	Male
XRN:KDA4NB	local	59	Female
XRN:43XU03	unknown	79	Male
XRN:L52FWH	local	59	Female
XRN:W3LNK5	met	32	Female
XRN:788G3H	unknown	61	Female
XRN:NTSC6P	unknown	21	Male
XRN:TUX7KD	local	33	Male
XRN:M94809	local	65	Male
XRN:XEDR0H	unknown	22	Male
XRN:VGMVCN	local	35	Male
XRN:FCF7W9	local	64	Male
XRN:JMCMA6	local	67	Female
XRN:QLMK9Y	local	50	Male
XRN:QU9S96	unknown	4	Female
XRN:27XQYX	met	33	Male
XRN:QPWA6A	met	45	Male
XRN:RLT898	local	37	Male
XRN:3C4DVP	met	69	Female
XRN:JY3V1G	met	63	Female
XRN:118X67	unknown	35	Female
XRN:418GB6	met	72	Female
XRN:CE7DU3	met	64	Male
XRN:A5C45H	local	69	Male
XRN:J4V011	met	59	Female
XRN:N7LNHC	unknown	1	Male
XRN:28ETRP	unknown	52	Female
XRN:JPE87T	local	89	Female
XRN:XY616S	ln	40	Female
XRN:9W1B3P	local	57	Female
XRN:VE5GU5	met	75	Female
XRN:9JPLTH	local	55	Female
XRN:83S2YD	ln	70	Female
XRN:R4R0V6	unknown	56	Male
XRN:LMQLH1	local	46	Female
XRN:NH1D7R	ln	58	Male
XRN:RNN3JP	unknown	0	Male
XRN:LNBFTX	unknown	1	Male
XRN:Y4TJSX	unknown	57	Female
XRN:H69JU7	unknown	57	Male
XRN:MQC79T	unknown	56	Female

de-identified ID	local_met_status	age [0-89] capped at 89	gender
XRN:9HUJC7	ln	63	Female
XRN:3C5SF7	unknown	2	Female
XRN:1TKQ9P	local	78	Female
XRN:6T11JS	ln	57	Female
XRN:PUSF3Q	met	69	Female
XRN:JMK9A9	local	49	Male
XRN:CJSFA7	ln	85	Female
XRN:FV4GFC	met	56	Male
XRN:ECBAS0	local	51	Female
XRN:NTBVMP	met	67	Female
XRN:JMRP5V	unknown	30	Female
XRN:TUHAWG	unknown	67	Female
XRN:2BY94V	ln	47	Female
XRN:R3QUNK	local	46	Female
XRN:7ENX9U	unknown	41	Female
XRN:5PYA6K	unknown	44	Male
XRN:337KN0	unknown	54	Male
XRN:VM6JUB	local	63	Male
XRN:F9QJQ4	unknown	60	Female
XRN:6XV1TC	local	48	Male
XRN:2EPU6V	local	58	Female
XRN:4V6C7D	met	76	Male
XRN:J967F9	local	69	Female
XRN:BFMQE2	met	66	Male
XRN:SYC44N	ln	53	Male
XRN:HLHABH	local	63	Male
XRN:2JUT87	local	66	Male
XRN:M30UQQ	local	6	Male
XRN:GXKRU9	met	82	Female
XRN:KKQP8K	local	45	Female
XRN:2MQNY7	unknown	52	Female
XRN:YBKE3A	local	14	Male
XRN:GBET4W	met	84	Male
XRN:A9YT8W	met	54	Male
XRN:A0L84C	ln	55	Female
XRN:U47G6W	local	49	Male
XRN:HGMD5B	unknown	0	Male
XRN:425Y9Y	met	57	Female
XRN:RQ15WB	met	66	Male
XRN:EAQNU2	local	65	Female
XRN:PEE2FX	local	65	Male
XRN:UQL2VU	met	69	Female
XRN:DNV68X	ln	47	Female
XRN:JPEWCE	unknown	71	Female
XRN:26RKR7	local	81	Female
XRN:ERQA98	unknown	54	Male
XRN:1KPTFL	met	70	Male
XRN:JEQHVJ	local	70	Male
XRN:D7YABS	unknown	56	Female
XRN:8NWA7N	local	46	Female
XRN:M6TB94	met	68	Male
XRN:X0NCE6	local	78	Male
XRN:3K7013	met	58	Female
XRN:WM9QUX	unknown	68	Male
XRN:1WBFEH	unknown	50	Female
XRN:EMHF6H	unknown	71	Female
XRN:M11VW2	local	47	Male
XRN:1UDN19	unknown	49	Female
XRN:9788AA	unknown	67	Female
XRN:77TSS7	unknown	49	Male

de-identified ID	local_met_status	age [0-89] capped at 89	gender
XRN:28CPKR	local	78	Male
XRN:YGBM2T	ln	83	Female
XRN:KPP0GJ	local	58	Female
XRN:9RATU5	met	46	Male
XRN:R928XV	unknown	75	Male
XRN:4C10PV	met	68	Male
XRN:KJ8ANN	met	67	Female
XRN:V6TQTM	met	60	Male
XRN:WHCAGS	met	54	Female
XRN:LLE1LJ	local	62	Male
XRN:TRT02U	ln	47	Female
XRN:SLXMB3	unknown	53	Female
XRN:TPD2K0	met	62	Female
XRN:260VLN	local	13	Male
XRN:V6XK12	unknown	5	Female
XRN:1FD60F	met	72	Female
XRN:X09XPA	met	71	Female
XRN:28KEFA	unknown	72	Male
XRN:QAW0A8	local	47	Female
XRN:M2RJ0A	unknown	37	Female
XRN:BPE7TN	local	62	Female
XRN:HB6RP0	local	65	Male
XRN:P8X1TX	local	74	Female
XRN:645RL5	unknown	45	Male
XRN:YUDWF3	met	66	Female
XRN:MCJYVR	unknown	34	Female
XRN:K9CXPR	met	65	Male
XRN:7KDS4J	unknown	26	Male
XRN:AKCAJN	unknown	0	Male
XRN:X0JTU3	local	73	Female
XRN:42CBCJ	met	89	Female
XRN:HYVXAM	met	57	Female
XRN:BS41G4	ln	50	Female
XRN:P76HJ7	ln	68	Female
XRN:7KKUX4	met	59	Male
XRN:2X1X9B	unknown	45	Female
XRN:2P397Y	met	56	Female
XRN:HVWBV8	unknown	87	Male
XRN:LEB8PE	unknown	17	Male
XRN:79DRDU	local	64	Female
XRN:1QEWMF	local	66	Female
XRN:P65K1J	unknown	46	Male
XRN:W6BMCA	local	43	Female
XRN:9176ED	unknown	77	Male
XRN:8SL29D	unknown	70	Male
XRN:WQR98C	met	63	Female
XRN:DWG2HB	ln	56	Female
XRN:J8M6Y4	local	59	Female
XRN:HGVV5R	local	69	Female
XRN:BEK9JM	unknown	77	Male
XRN:ARHQCV	local	29	Male
XRN:CA8JMU	local	77	Male
XRN:NM70AW	met	61	Female
XRN:W7W6AA	ln	54	Female
XRN:NYQ3R4	unknown	56	Female
XRN:7FKT7J	unknown	42	Male
XRN:HGG3PQ	local	69	Male
XRN:GN39EH	met	73	Female
XRN:19334K	local	55	Male
XRN:0JDM59	unknown	66	Female

de-identified ID	local_met_status	age [0-89] capped at 89	gender
XRN:MJGQ34	unknown	55	Female
XRN:8UF065	local	20	Male
XRN:1FDH7P	ln	59	Female
XRN:4T6T19	unknown	27	Female
XRN:920TTL	unknown	33	Female
XRN:NS8VXF	ln	45	Female
XRN:9H2FK1	unknown	39	Male
XRN:K5T5LL	ln	64	Female
XRN:X0BSV8	met	52	Female
XRN:YGXF7Y	local	62	Female
XRN:MWQ1RA	local	67	Female
XRN:B6Q7DK	unknown	40	Male
XRN:JXNECQ	unknown	61	Female
XRN:FVTVDK	ln	78	Female
XRN:3WDTFT	ln	53	Female
XRN:R3BXP2	local	84	Female
XRN:6MKST2	met	68	Male
XRN:LP4LM4	unknown	61	Male
XRN:MM17NJ	local	64	Female
XRN:QKV81G	met	53	Female
XRN:34KAAH	met	58	Female
XRN:JTCFL6	ln	70	Female
XRN:19DP4T	local	53	Female
XRN:S3SYT5	unknown	55	Female
XRN:2K42C7	unknown	56	Female
XRN:GQPXV8	local	25	Male
XRN:R1N6VR	ln	55	Male
XRN:GY5L2V	unknown	56	Male
XRN:NS4MYL	met	62	Female
XRN:8CQ5AU	met	62	Male
XRN:TS42KN	unknown	72	Female
XRN:CEK4PW	local	64	Male
XRN:2HFK3R	met	71	Female
XRN:6E1K5P	unknown	85	Male
XRN:2XSYVT	local	53	Female
XRN:4NG9M1	met	52	Female
XRN:5UJ7GB	unknown	76	Female
XRN:N6XA9F	met	62	Female
XRN:DVM2CN	local	52	Female
XRN:ET9D2Q	unknown	39	Female
XRN:885U6F	ln	39	Female
XRN:FG1DT2	local	61	Female
XRN:HYG0D6	met	71	Male
XRN:TUPSWP	unknown	49	Female
XRN:F17P4Q	local	83	Male
XRN:78B55P	local	41	Female
XRN:6APH0B	unknown	46	Female
XRN:WPPM51	local	70	Female
XRN:R3AKX8	ln	68	Male
XRN:2PQWCE	unknown	11	Male
XRN:GH4WQ3	unknown	54	Male

Supplementary Table 2. Prevalence of *NTRK* fusion-positive specimens in FoundationCORE by tumour histology

Disease ontology	Disease group	Disease ontology counts (all)	Count altered in disease ontology (all)	Percent altered in disease ontology (all)	Disease ontology counts (adult)	Count altered in disease ontology (adult)	Percent altered in disease ontology (adult)	Disease ontology counts (paediatric)	Count altered in disease ontology (paediatric)	Percent altered in disease ontology (paediatric)
Salivary gland mammary analogue secretory carcinoma (MASC)	Salivary gland	14	10	71.43%	12	8	66.67%	2	2	100.00%
Unknown primary myoepithelial carcinoma	Unknown primary carcinoma (CUP)	7	1	14.29%	5	1	20.00%	2	0	0.00%
Soft tissue fibrosarcoma	Soft tissue sarcoma	136	16	11.76%	109	3	2.75%	25	13	52.00%
Salivary gland undifferentiated carcinoma	Salivary gland	13	1	7.69%	13	1	7.69%	0	.	.
Ovary sarcoma	Soft tissue sarcoma	17	1	5.88%	17	1	5.88%	0	.	.
Small intestine leiomyosarcoma	Leiomyosarcoma	21	1	4.76%	21	1	4.76%	0	.	.
Soft tissue haemangioma	Soft tissue sarcoma	21	1	4.76%	10	0	0.00%	11	1	9.09%
Spine glioma (NOS)	Glioma	24	1	4.17%	14	0	0.00%	10	1	10.00%
Salivary gland myoepithelial carcinoma	Salivary gland	59	2	3.39%	58	2	3.45%	0	.	.
Soft tissue malignant peripheral nerve sheath tumour (MPNST)	Soft tissue sarcoma	382	12	3.14%	337	10	2.97%	43	2	4.65%
Soft tissue rhabdomyosarcoma pleomorphic	Rhabdomyosarcoma	34	1	2.94%	33	1	3.03%	1	0	0.00%
Bone sarcoma (NOS)	Bone sarcoma	36	1	2.78%	29	0	0.00%	5	0	0.00%
Salivary gland acinic cell tumour	Salivary gland	186	5	2.69%	183	5	2.73%	2	0	0.00%
Thyroid papillary carcinoma	Thyroid	1083	28	2.59%	1038	19	1.83%	40	9	22.50%
Salivary gland adenocarcinoma	Salivary gland	356	9	2.53%	352	8	2.27%	2	1	50.00%
Soft tissue inflammatory myofibroblastic tumour	Soft tissue sarcoma	81	2	2.47%	44	1	2.27%	36	1	2.78%
Uterus adenosarcoma	Uterus	84	2	2.38%	84	2	2.38%	0	.	.
Breast sarcoma	Soft tissue sarcoma	43	1	2.33%	43	1	2.33%	0	.	.
Fallopian tube carcinosarcoma	Fallopian tube	44	1	2.27%	44	1	2.27%	0	.	.
Unknown primary sarcoma	Soft tissue sarcoma	93	2	2.15%	86	2	2.33%	7	0	0.00%
Brain pleomorphic xanthoastrocytoma	Glioma	49	1	2.04%	32	0	0.00%	17	1	5.88%
Uterus neuroendocrine carcinoma	Female-neuro	51	1	1.96%	51	1	1.96%	0	.	.

Disease ontology	Disease group	Disease ontology counts (all)	Count altered in disease ontology (all)	Percent altered in disease ontology (all)	Disease ontology counts (adult)	Count altered in disease ontology (adult)	Percent altered in disease ontology (adult)	Disease ontology counts (paediatric)	Count altered in disease ontology (paediatric)	Percent altered in disease ontology (paediatric)
Salivary gland carcinoma ex pleomorphic adenoma	Salivary gland	54	1	1.85%	54	1	1.85%	0	.	.
Soft tissue sarcoma (NOS)	Soft tissue sarcoma	2328	43	1.85%	2173	37	1.70%	146	6	4.11%
Soft tissue primitive neuroectoderm tumour (PNET)	Soft tissue sarcoma	56	1	1.79%	47	0	0.00%	9	1	11.11%
Breast angiosarcoma	Angiosarcoma	115	2	1.74%	113	1	0.88%	0	.	.
Salivary gland mucoepidermoid carcinoma	Salivary gland	130	2	1.54%	129	2	1.55%	1	0	0.00%
Salivary gland carcinoma (NOS)	Salivary gland	464	7	1.51%	462	7	1.52%	1	0	0.00%
Soft tissue sarcoma undifferentiated	Soft tissue sarcoma	422	6	1.42%	396	5	1.26%	20	1	5.00%
Uterus endometrial stromal sarcoma	Uterus sarcoma	286	4	1.40%	284	4	1.41%	0	.	.
Soft tissue osteosarcoma (extraskeletal)	Bone sarcoma	75	1	1.33%	70	1	1.43%	5	0	0.00%
Nasopharynx and paranasal sinuses adenocarcinoma	Head and neck	77	1	1.30%	77	1	1.30%	0	.	.
Soft tissue myxofibrosarcoma	Soft tissue sarcoma	232	3	1.29%	229	3	1.31%	1	0	0.00%
Soft tissue perivascular epithelioid cell tumour (PEComa)	Soft tissue sarcoma	87	1	1.15%	85	1	1.18%	2	0	0.00%
Vulva melanoma	Melanoma	87	1	1.15%	87	1	1.15%	0	.	.
Brain astrocytoma pilocytic	Glioma	371	4	1.08%	137	1	0.73%	232	3	1.29%
Soft tissue liposarcoma	Soft tissue sarcoma	1215	13	1.07%	1202	13	1.08%	9	0	0.00%
Schwannoma	Peripheral nervous system (PNS)	94	1	1.06%	83	0	0.00%	11	1	9.09%
Thyroid anaplastic carcinoma	Thyroid	511	5	0.98%	505	5	0.99%	0	.	.
Brain anaplastic oligodendroglioma	Glioma	104	1	0.96%	103	1	0.97%	1	0	0.00%
Bone chondrosarcoma	Chondrosarcoma	238	2	0.84%	233	1	0.43%	4	1	25.00%
Unknown primary GIST	GIST	611	5	0.82%	604	5	0.83%	5	0	0.00%
Thyroid carcinoma (NOS)	Thyroid	507	4	0.79%	506	4	0.79%	1	0	0.00%
Unknown primary leiomyosarcoma	Leiomyosarcoma	264	2	0.76%	262	2	0.76%	2	0	0.00%
Breast metaplastic carcinoma	Breast	399	3	0.75%	397	3	0.76%	0	.	.

Disease ontology	Disease group	Disease ontology counts (all)	Count altered in disease ontology (all)	Percent altered in disease ontology (all)	Disease ontology counts (adult)	Count altered in disease ontology (adult)	Percent altered in disease ontology (adult)	Disease ontology counts (paediatric)	Count altered in disease ontology (paediatric)	Percent altered in disease ontology (paediatric)
Fallopian tube adenocarcinoma	Fallopian tube	140	1	0.71%	138	1	0.72%	0	.	.
Prostate ductal adenocarcinoma	Prostate	142	1	0.70%	141	1	0.71%	0	.	.
Uterus endometrial adenocarcinoma mixed histology	Endometrial	149	1	0.67%	149	1	0.67%	0	.	.
Small intestine neuroendocrine carcinoma	GI-neuro	151	1	0.66%	150	1	0.67%	1	0	0.00%
Rhabdomyosarcoma (NOS)	Rhabdomyosarcoma	312	2	0.64%	194	1	0.52%	113	1	0.88%
Bone osteosarcoma	Bone sarcoma	789	5	0.63%	503	2	0.40%	279	2	0.72%
Bladder squamous cell carcinoma (SCC)	Bladder	160	1	0.63%	160	1	0.63%	0	.	.
Soft tissue desmoplastic small round cell tumour	Soft tissue sarcoma	167	1	0.60%	121	1	0.83%	46	0	0.00%
Uterus leiomyosarcoma	Leiomyosarcoma	1014	6	0.59%	1011	6	0.59%	0	.	.
Head and neck carcinoma (NOS)	Head and neck	173	1	0.58%	172	1	0.58%	1	0	0.00%
Pancreas acinar cell carcinoma	Pancreas	173	1	0.58%	169	1	0.59%	2	0	0.00%
Salivary gland duct carcinoma	Salivary gland	177	1	0.56%	177	1	0.56%	0	.	.
Brain anaplastic astrocytoma	Glioma	957	5	0.52%	866	5	0.58%	89	0	0.00%
Unknown primary neuroendocrine tumour	Unknown primary-neuro	385	2	0.52%	382	2	0.52%	2	0	0.00%
Adrenal gland neuroblastoma	Endocrine-neuro	193	1	0.52%	16	0	0.00%	176	1	0.57%
Unknown primary sarcomatoid carcinoma	Unknown primary carcinoma (CUP)	197	1	0.51%	197	1	0.51%	0	.	.
Adrenal gland cortical carcinoma	Adrenal gland	592	3	0.51%	566	3	0.53%	22	0	0.00%
Peritoneum serous carcinoma	Peritoneum	795	4	0.50%	790	4	0.51%	0	.	.
Uterus sarcoma (NOS)	Uterus sarcoma	203	1	0.49%	201	1	0.50%	2	0	0.00%
Lung large cell carcinoma	Non-small cell lung carcinoma (NSCLC)	211	1	0.47%	210	1	0.48%	0	.	.
Soft tissue solitary fibrous tumour	Solitary fibrous tumour	212	1	0.47%	209	0	0.00%	2	1	50.00%
Thyroid follicular carcinoma	Thyroid	218	1	0.46%	214	1	0.47%	4	0	0.00%
Bladder adenocarcinoma	Bladder	222	1	0.45%	220	1	0.45%	0	.	.
Stomach GIST	GIST	456	2	0.44%	449	2	0.45%	6	0	0.00%
Lung large cell neuroendocrine carcinoma	Non-small cell lung carcinoma (NSCLC)	923	4	0.43%	919	4	0.44%	0	.	.

Disease ontology	Disease group	Disease ontology counts (all)	Count altered in disease ontology (all)	Percent altered in disease ontology (all)	Disease ontology counts (adult)	Count altered in disease ontology (adult)	Percent altered in disease ontology (adult)	Disease ontology counts (paediatric)	Count altered in disease ontology (paediatric)	Percent altered in disease ontology (paediatric)
Brain glioma (NOS)	Glioma	934	4	0.43%	688	1	0.15%	240	3	1.25%
Breast carcinoma (NOS)	Breast	16830	72	0.43%	16778	71	0.42%	2	1	50.00%
Ovary high grade serous carcinoma	Ovary	2159	9	0.42%	2155	9	0.42%	0	.	.
Brain glioblastoma (GBM)	Glioma	6673	27	0.40%	6434	24	0.37%	219	3	1.37%
Nasopharynx and paranasal sinuses undifferentiated carcinoma	Head and neck	256	1	0.39%	243	1	0.41%	11	0	0.00%
Breast invasive ductal carcinoma (IDC)	Breast	11150	42	0.38%	11104	42	0.38%	1	0	0.00%
Ovary carcinosarcoma	Ovary	548	2	0.36%	548	2	0.36%	0	.	.
Brain astrocytoma	Glioma	839	3	0.36%	721	2	0.28%	115	1	0.87%
Bladder urothelial (transitional cell) carcinoma	Bladder	4611	16	0.35%	4605	16	0.35%	1	0	0.00%
Ovary serous carcinoma	Ovary	8327	28	0.34%	8301	28	0.34%	6	0	0.00%
Small intestine GIST	GIST	301	1	0.33%	299	1	0.33%	1	0	0.00%
Unknown primary melanoma	Melanoma	3842	12	0.31%	3813	11	0.29%	12	1	8.33%
Oesophagus adenocarcinoma	Oesophagus	4290	13	0.30%	4279	13	0.30%	1	0	0.00%
Ovary epithelial carcinoma	Ovary	2973	9	0.30%	2964	9	0.30%	3	0	0.00%
Eye intraocular melanoma	Melanoma	333	1	0.30%	333	1	0.30%	0	.	.
Unknown primary adenocarcinoma	Unknown primary carcinoma (CUP)	8058	24	0.30%	8023	24	0.30%	5	0	0.00%
Uterus endometrial adenocarcinoma papillary serous	Endometrial	2015	6	0.30%	2013	6	0.30%	0	.	.
Gallbladder adenocarcinoma	Biliary	1716	5	0.29%	1711	5	0.29%	0	.	.
Brain medulloblastoma	CNS non-glioma	347	1	0.29%	148	0	0.00%	197	1	0.51%
Soft tissue leiomyosarcoma	Leiomyosarcoma	1432	4	0.28%	1421	4	0.28%	6	0	0.00%
Unknown primary urothelial carcinoma	Unknown primary carcinoma (CUP)	722	2	0.28%	721	2	0.28%	0	.	.
Prostate neuroendocrine carcinoma	Male-neuro	374	1	0.27%	372	1	0.27%	1	0	0.00%
Lung adenocarcinoma	Non-small cell lung carcinoma (NSCLC)	37015	95	0.26%	36897	95	0.26%	7	0	0.00%
Bile duct adenocarcinoma	Biliary	784	2	0.26%	783	2	0.26%	0	.	.

Disease ontology	Disease group	Disease ontology counts (all)	Count altered in disease ontology (all)	Percent altered in disease ontology (all)	Disease ontology counts (adult)	Count altered in disease ontology (adult)	Percent altered in disease ontology (adult)	Disease ontology counts (paediatric)	Count altered in disease ontology (paediatric)	Percent altered in disease ontology (paediatric)
Colon adenocarcinoma (CRC)	Colorectal (CRC)	28939	73	0.25%	28842	73	0.25%	25	0	0.00%
Soft tissue angiosarcoma	Angiosarcoma	412	1	0.24%	400	1	0.25%	9	0	0.00%
Lung sarcomatoid carcinoma	Non-small cell lung carcinoma (NSCLC)	424	1	0.24%	422	1	0.24%	0	.	.
Pancreas islet cell tumour	Endocrine-neuro	859	2	0.23%	852	2	0.23%	6	0	0.00%
Fallopian tube serous carcinoma	Fallopian tube	1305	3	0.23%	1305	3	0.23%	0	.	.
Non-small cell lung carcinoma (NSCLC) (NOS)	Non-small cell lung carcinoma (NSCLC)	8294	19	0.23%	8258	19	0.23%	2	0	0.00%
Uterus endometrial adenocarcinoma (NOS)	Endometrial	3494	8	0.23%	3490	8	0.23%	0	.	.
Uterus carcinosarcoma	Uterus	1312	3	0.23%	1312	3	0.23%	0	.	.
Ovary clear cell carcinoma	Ovary	891	2	0.22%	890	2	0.22%	0	.	.
Prostate acinar adenocarcinoma	Prostate	9056	20	0.22%	9035	20	0.22%	0	.	.
Cervix squamous cell carcinoma (SCC)	Cervix	1368	3	0.22%	1363	3	0.22%	1	0	0.00%
Soft tissue Ewing sarcoma	Ewing sarcoma	474	1	0.21%	292	1	0.34%	177	0	0.00%
Unknown primary carcinoma (CUP) (NOS)	Unknown primary carcinoma (CUP)	4296	9	0.21%	4269	9	0.21%	7	0	0.00%
Liver cholangiocarcinoma	Cholangiocarcinoma	5377	11	0.20%	5363	11	0.21%	6	0	0.00%
Soft tissue synovial sarcoma	Soft tissue sarcoma	499	1	0.20%	467	1	0.21%	31	0	0.00%
Unknown primary serous carcinoma	Unknown primary carcinoma (CUP)	506	1	0.20%	506	1	0.20%	0	.	.
Ovary endometrioid adenocarcinoma	Ovary	549	1	0.18%	549	1	0.18%	0	.	.
Stomach adenocarcinoma (NOS)	Stomach	4458	8	0.18%	4436	8	0.18%	9	0	0.00%
Gastroesophageal junction adenocarcinoma	Oesophagus	1695	3	0.18%	1686	3	0.18%	2	0	0.00%
Skin melanoma	Melanoma	3449	6	0.17%	3424	6	0.18%	14	0	0.00%
Lung squamous cell carcinoma (SCC)	Non-small cell lung carcinoma (NSCLC)	9248	16	0.17%	9235	16	0.17%	2	0	0.00%
Oesophagus squamous cell carcinoma (SCC)	Oesophagus	1163	2	0.17%	1161	2	0.17%	0	.	.
Pancreas ductal adenocarcinoma	Pancreas	12225	21	0.17%	12195	21	0.17%	1	0	0.00%
Pancreatobiliary carcinoma	Pancreas	2388	4	0.17%	2385	4	0.17%	0	.	.

Disease ontology	Disease group	Disease ontology counts (all)	Count altered in disease ontology (all)	Percent altered in disease ontology (all)	Disease ontology counts (adult)	Count altered in disease ontology (adult)	Percent altered in disease ontology (adult)	Disease ontology counts (paediatric)	Count altered in disease ontology (paediatric)	Percent altered in disease ontology (paediatric)
Head and neck squamous cell carcinoma (HNSCC)	Head and neck	3998	6	0.15%	3987	6	0.15%	3	0	0.00%
Duodenum adenocarcinoma	Small intestine	680	1	0.15%	677	1	0.15%	0	.	.
Kidney urothelial carcinoma	Kidney	715	1	0.14%	711	1	0.14%	0	.	.
Cervix adenocarcinoma	Cervix	781	1	0.13%	777	1	0.13%	2	0	0.00%
Small intestine adenocarcinoma	Small intestine	782	1	0.13%	780	1	0.13%	1	0	0.00%
Unknown primary undifferentiated neuroendocrine carcinoma	Unknown primary-neuro	1630	2	0.12%	1617	2	0.12%	7	0	0.00%
Skin squamous cell carcinoma (SCC)	Skin	865	1	0.12%	858	1	0.12%	4	0	0.00%
Unknown primary squamous cell carcinoma (SCC)	Unknown primary carcinoma (CUP)	1765	2	0.11%	1760	2	0.11%	1	0	0.00%
Pancreas carcinoma (NOS)	Pancreas	1868	2	0.11%	1863	2	0.11%	0	.	.
Rectum adenocarcinoma (CRC)	Colorectal (CRC)	5606	4	0.07%	5596	4	0.07%	1	0	0.00%
Lung small cell undifferentiated carcinoma	Small cell	2977	2	0.07%	2971	2	0.07%	2	0	0.00%
Breast invasive lobular carcinoma (ILC)	Breast	1549	1	0.06%	1545	1	0.06%	0	.	.
Kidney clear cell carcinoma	Kidney	1591	1	0.06%	1585	1	0.06%	2	0	0.00%
Liver hepatocellular carcinoma (HCC)	Liver	1622	1	0.06%	1587	1	0.06%	24	0	0.00%
Kidney renal cell carcinoma	Kidney	1905	1	0.05%	1887	1	0.05%	12	0	0.00%
Uterus endometrial adenocarcinoma endometrioid	Endometrial	2187	1	0.05%	2187	1	0.05%	0	.	.
Ovary granulosa cell tumour	Ovary	450	0	0.00%	436	0	0.00%	12	0	0.00%
Bladder carcinoma (NOS)	Bladder	285	0	0.00%	284	0	0.00%	1	0	0.00%
Liver hepatoblastoma	Liver	71	0	0.00%	2	0	0.00%	69	0	0.00%
Stomach adenocarcinoma diffuse type	Stomach	433	0	0.00%	431	0	0.00%	1	0	0.00%
Liver embryonal sarcoma	Liver sarcoma	5	0	0.00%	1	0	0.00%	4	0	0.00%
Kidney collecting duct carcinoma	Kidney	63	0	0.00%	63	0	0.00%	0	.	.
Soft tissue chondrosarcoma	Chondrosarcoma	289	0	0.00%	281	0	0.00%	8	0	0.00%
Kidney sarcoma (NOS)	Kidney sarcoma	26	0	0.00%	24	0	0.00%	2	0	0.00%

Disease ontology	Disease group	Disease ontology counts (all)	Count altered in disease ontology (all)	Percent altered in disease ontology (all)	Disease ontology counts (adult)	Count altered in disease ontology (adult)	Percent altered in disease ontology (adult)	Disease ontology counts (paediatric)	Count altered in disease ontology (paediatric)	Percent altered in disease ontology (paediatric)
Soft tissue NUT midline carcinoma	Soft tissue sarcoma	31	0	0.00%	27	0	0.00%	4	0	0.00%
Colon neuroendocrine carcinoma	GI-neuro	387	0	0.00%	387	0	0.00%	0	.	.
Liver haemangioendothelioma	Liver sarcoma	18	0	0.00%	17	0	0.00%	0	.	.
Breast neuroendocrine carcinoma	Female-neuro	83	0	0.00%	83	0	0.00%	0	.	.
Soft tissue pigmented villonodular synovitis	Soft tissue sarcoma	5	0	0.00%	4	0	0.00%	1	0	0.00%
Ureter small cell carcinoma	Small cell	10	0	0.00%	10	0	0.00%	0	.	.
Head and neck Schneiderian papilloma	Head and neck	5	0	0.00%	5	0	0.00%	0	.	.
Lung adenoid cystic carcinoma	Adenoid cystic carcinoma	150	0	0.00%	149	0	0.00%	0	.	.
Liver angiosarcoma	Angiosarcoma	32	0	0.00%	27	0	0.00%	5	0	0.00%
Gallbladder squamous cell carcinoma (SCC)	Biliary	28	0	0.00%	28	0	0.00%	0	.	.
Anus basaloid carcinoma	Anus	37	0	0.00%	37	0	0.00%	0	.	.
Ovary female adnexal tumour of Wolffian origin	Ovary	6	0	0.00%	6	0	0.00%	0	.	.
Skin sarcoma (NOS)	Skin sarcoma	23	0	0.00%	21	0	0.00%	1	0	0.00%
Brain astrocytoma pilomyxoid	Glioma	49	0	0.00%	10	0	0.00%	39	0	0.00%
Appendix goblet cell carcinoid (GCC)	Carcinoid	180	0	0.00%	179	0	0.00%	1	0	0.00%
Brain ependymoma	CNS non-glioma	277	0	0.00%	159	0	0.00%	117	0	0.00%
Vagina neuroendocrine carcinoma	Female-neuro	13	0	0.00%	13	0	0.00%	0	.	.
Brain meningeal sarcoma	CNS sarcoma	7	0	0.00%	5	0	0.00%	1	0	0.00%
Urethra adenocarcinoma	Urinary	39	0	0.00%	39	0	0.00%	0	.	.
Testis Sertoli cell tumour	Testis	11	0	0.00%	10	0	0.00%	1	0	0.00%
Pituitary carcinoma	Endocrine-neuro	16	0	0.00%	16	0	0.00%	0	.	.
Oesophagus carcinoma (NOS)	Oesophagus	305	0	0.00%	305	0	0.00%	0	.	.
Skin adnexal carcinoma	Skin	219	0	0.00%	217	0	0.00%	1	0	0.00%
Cervix undifferentiated carcinoma	Cervix	76	0	0.00%	76	0	0.00%	0	.	.
Prostate sarcoma	Soft tissue sarcoma	7	0	0.00%	7	0	0.00%	0	.	.
Prostate carcinosarcoma	Prostate	5	0	0.00%	5	0	0.00%	0	.	.

Disease ontology	Disease group	Disease ontology counts (all)	Count altered in disease ontology (all)	Percent altered in disease ontology (all)	Disease ontology counts (adult)	Count altered in disease ontology (adult)	Percent altered in disease ontology (adult)	Disease ontology counts (paediatric)	Count altered in disease ontology (paediatric)	Percent altered in disease ontology (paediatric)
Cervix neuroendocrine carcinoma	Female-neuro	125	0	0.00%	125	0	0.00%	0	.	.
Fallopian tube carcinoma mixed histology	Fallopian tube	9	0	0.00%	9	0	0.00%	0	.	.
Nasopharynx and paranasal sinuses squamous cell carcinoma (SCC)	Head and neck	162	0	0.00%	161	0	0.00%	1	0	0.00%
Placenta choriocarcinoma	Placenta	18	0	0.00%	17	0	0.00%	1	0	0.00%
Small intestine gastrointestinal neuroectodermal tumour	GI-neuro	15	0	0.00%	14	0	0.00%	1	0	0.00%
Uterus endometrial squamous cell carcinoma (SCC)	Endometrial	12	0	0.00%	12	0	0.00%	0	.	.
Soft tissue myopericytoma	Soft tissue sarcoma	5	0	0.00%	3	0	0.00%	2	0	0.00%
Head and neck spindle cell carcinoma	Head and neck	18	0	0.00%	18	0	0.00%	0	.	.
Lung carcinosarcoma	Non-small cell lung carcinoma (NSCLC)	52	0	0.00%	52	0	0.00%	0	.	.
Pineal parenchymal tumour	Pineal gland	30	0	0.00%	25	0	0.00%	5	0	0.00%
Ovary sex cord tumour with annular tubules (SCTAT)	Ovary	4	0	0.00%	4	0	0.00%	0	.	.
Ovary Sertoli-Leydig cell tumour	Germ cell	12	0	0.00%	10	0	0.00%	2	0	0.00%
Pancreas mucinous cystadenocarcinoma	Pancreas	27	0	0.00%	27	0	0.00%	0	.	.
Cervix clear cell adenocarcinoma	Cervix	57	0	0.00%	53	0	0.00%	4	0	0.00%
Lung atypical carcinoid	Carcinoid	212	0	0.00%	211	0	0.00%	1	0	0.00%
Brain meningioma	CNS non-glioma	889	0	0.00%	859	0	0.00%	25	0	0.00%
Penis squamous cell carcinoma (SCC)	Male genital	218	0	0.00%	218	0	0.00%	0	.	.
Retroperitoneum germ cell tumour	Germ cell	13	0	0.00%	10	0	0.00%	3	0	0.00%
Testis germ cell tumour (mixed)	Germ cell	42	0	0.00%	39	0	0.00%	3	0	0.00%
Testis germ cell tumour (seminoma)	Germ cell	44	0	0.00%	44	0	0.00%	0	.	.
Lung adenosquamous carcinoma	Non-small cell lung carcinoma (NSCLC)	448	0	0.00%	447	0	0.00%	0	.	.

Disease ontology	Disease group	Disease ontology counts (all)	Count altered in disease ontology (all)	Percent altered in disease ontology (all)	Disease ontology counts (adult)	Count altered in disease ontology (adult)	Percent altered in disease ontology (adult)	Disease ontology counts (paediatric)	Count altered in disease ontology (paediatric)	Percent altered in disease ontology (paediatric)
Brain embryonal tumour	CNS non-glioma	47	0	0.00%	10	0	0.00%	37	0	0.00%
Gallbladder carcinoma	Biliary	74	0	0.00%	74	0	0.00%	0	.	.
Unknown primary adenosarcoma	Unknown primary carcinoma (CUP)	11	0	0.00%	11	0	0.00%	0	.	.
Small intestine carcinoid	Carcinoid	129	0	0.00%	127	0	0.00%	1	0	0.00%
Anus melanoma	Melanoma	140	0	0.00%	139	0	0.00%	1	0	0.00%
Thyroid medullary carcinoma	Endocrine-neuro	335	0	0.00%	329	0	0.00%	5	0	0.00%
Brain haemangiopericytoma	CNS non-glioma	59	0	0.00%	57	0	0.00%	1	0	0.00%
Soft tissue fibromatosis	Soft tissue sarcoma	294	0	0.00%	204	0	0.00%	89	0	0.00%
Spleen angiosarcoma	Angiosarcoma	13	0	0.00%	13	0	0.00%	0	.	.
Lung solitary fibrous tumour	Solitary fibrous tumour	53	0	0.00%	53	0	0.00%	0	.	.
Parathyroid carcinoma	Endocrine-neuro	42	0	0.00%	41	0	0.00%	1	0	0.00%
Stomach neuroendocrine carcinoma	GI-neuro	113	0	0.00%	112	0	0.00%	1	0	0.00%
Ovary low grade serous carcinoma	Ovary	146	0	0.00%	146	0	0.00%	0	.	.
Ovary germ cell tumour	Germ cell	92	0	0.00%	59	0	0.00%	33	0	0.00%
Salivary gland adenoid cystic carcinoma	Adenoid cystic carcinoma	482	0	0.00%	474	0	0.00%	2	0	0.00%
Pancreas adenosquamous carcinoma	Pancreas	51	0	0.00%	51	0	0.00%	0	.	.
Brain choroid plexus tumour	CNS non-glioma	50	0	0.00%	22	0	0.00%	28	0	0.00%
Adrenal gland pheochromocytoma	Endocrine-neuro	90	0	0.00%	84	0	0.00%	6	0	0.00%
Ovary neuroendocrine carcinoma	Female-neuro	56	0	0.00%	56	0	0.00%	0	.	.
Kidney Wilms' tumour	Kidney	154	0	0.00%	24	0	0.00%	130	0	0.00%
Brain ganglioglioma	Glioma	105	0	0.00%	57	0	0.00%	48	0	0.00%
Head and neck adenoid cystic carcinoma	Adenoid cystic carcinoma	525	0	0.00%	518	0	0.00%	0	.	.
Duodenum neuroendocrine carcinoma	GI-neuro	13	0	0.00%	13	0	0.00%	0	.	.
Brain oligodendroglioma	Glioma	572	0	0.00%	551	0	0.00%	21	0	0.00%
Brain anaplastic meningioma	Glioma	64	0	0.00%	63	0	0.00%	1	0	0.00%
Heart sarcoma (NOS)	Heart sarcoma	30	0	0.00%	27	0	0.00%	3	0	0.00%

Disease ontology	Disease group	Disease ontology counts (all)	Count altered in disease ontology (all)	Percent altered in disease ontology (all)	Disease ontology counts (adult)	Count altered in disease ontology (adult)	Percent altered in disease ontology (adult)	Disease ontology counts (paediatric)	Count altered in disease ontology (paediatric)	Percent altered in disease ontology (paediatric)
Brain haemangioblastoma	CNS sarcoma	33	0	0.00%	31	0	0.00%	2	0	0.00%
Vagina squamous cell carcinoma (SCC)	Female genital	157	0	0.00%	157	0	0.00%	0	.	.
Fallopian tube endometrioid carcinoma	Fallopian tube	6	0	0.00%	6	0	0.00%	0	.	.
Head and neck aesthesioneuroblastoma	Head and neck-neuro	61	0	0.00%	60	0	0.00%	1	0	0.00%
Soft tissue extrarenal rhabdoid tumour	Soft tissue sarcoma	16	0	0.00%	5	0	0.00%	11	0	0.00%
Breast carcinosarcoma	Breast	11	0	0.00%	11	0	0.00%	0	.	.
Brain atypical teratoid rhabdoid tumour (ATRT)	CNS non-glioma	55	0	0.00%	9	0	0.00%	45	0	0.00%
Soft tissue alveolar soft part sarcoma	Soft tissue sarcoma	84	0	0.00%	68	0	0.00%	16	0	0.00%
Stomach adenocarcinoma intestinal type	Stomach	154	0	0.00%	154	0	0.00%	0	.	.
Unknown primary undifferentiated small cell carcinoma	Small cell	363	0	0.00%	361	0	0.00%	0	.	.
Soft tissue myoepithelial carcinoma	Soft tissue sarcoma	44	0	0.00%	39	0	0.00%	5	0	0.00%
Urethra squamous cell carcinoma (SCC)	Urinary	34	0	0.00%	34	0	0.00%	0	.	.
Lung mucoepidermoid carcinoma	Lung salivary gland-type	28	0	0.00%	25	0	0.00%	3	0	0.00%
Ovary sex cord stromal tumour (NOS)	Ovary	69	0	0.00%	61	0	0.00%	7	0	0.00%
Ovary serous tumor (LMP)	Ovary	43	0	0.00%	43	0	0.00%	0	.	.
Uterus malignant mixed mesodermal tumour (MMMT)	Malignant mixed mesodermal tumour (MMMT)	11	0	0.00%	11	0	0.00%	0	.	.
Paraganglioma	Peripheral nervous system (PNS)	137	0	0.00%	123	0	0.00%	13	0	0.00%

Disease ontology	Disease group	Disease ontology counts (all)	Count altered in disease ontology (all)	Percent altered in disease ontology (all)	Disease ontology counts (adult)	Count altered in disease ontology (adult)	Percent altered in disease ontology (adult)	Disease ontology counts (paediatric)	Count altered in disease ontology (paediatric)	Percent altered in disease ontology (paediatric)
Uterus endometrial small cell carcinoma	Small cell	18	0	0.00%	18	0	0.00%	0	.	.
Kidney medullary carcinoma	Kidney	49	0	0.00%	43	0	0.00%	6	0	0.00%
Anus carcinoma (NOS)	Anus	32	0	0.00%	32	0	0.00%	0	.	.
Uterus gestational trophoblastic tumour epithelioid	Placenta	4	0	0.00%	4	0	0.00%	0	.	.
Soft tissue ossifying fibromyxoid tumour	Soft tissue sarcoma	11	0	0.00%	10	0	0.00%	1	0	0.00%
Soft tissue epithelioid sarcoma	Soft tissue sarcoma	85	0	0.00%	72	0	0.00%	12	0	0.00%
Unknown primary carcinoid	Carcinoid	166	0	0.00%	165	0	0.00%	1	0	0.00%
Soft tissue granular cell tumour	Soft tissue sarcoma	31	0	0.00%	30	0	0.00%	1	0	0.00%
Nasopharynx and paranasal sinuses lymphoepithelioma	Head and neck	6	0	0.00%	6	0	0.00%	0	.	.
Rectum squamous cell carcinoma (SCC)	Colorectal (CRC)	152	0	0.00%	152	0	0.00%	0	.	.
Teratoma	Germ cell	36	0	0.00%	31	0	0.00%	5	0	0.00%
Mediastinum germ cell tumour	Germ cell	56	0	0.00%	48	0	0.00%	8	0	0.00%
Skin Kaposi sarcoma	Kaposi sarcoma	22	0	0.00%	22	0	0.00%	0	.	.
Brain melanocytoma	CNS non-glioma	6	0	0.00%	5	0	0.00%	1	0	0.00%
Head and neck ameloblastoma	Head and neck-neuro	18	0	0.00%	18	0	0.00%	0	.	.
Bladder small cell carcinoma	Small cell	89	0	0.00%	89	0	0.00%	0	.	.
Liver hepatocellular carcinoma (FLO)	Liver	68	0	0.00%	43	0	0.00%	25	0	0.00%
Pancreas pancreatoblastoma	Pancreas	7	0	0.00%	4	0	0.00%	3	0	0.00%
Rectum carcinoid tumour	Carcinoid	23	0	0.00%	23	0	0.00%	0	.	.
Thymus atypical carcinoid	Carcinoid	6	0	0.00%	6	0	0.00%	0	.	.
Soft tissue angiomyolipoma	Soft tissue sarcoma	25	0	0.00%	24	0	0.00%	0	.	.
Head and neck adenocarcinoma	Head and neck	55	0	0.00%	53	0	0.00%	1	0	0.00%
Liver mixed hepatocellular cholangiocarcinoma	Cholangiocarcinoma	74	0	0.00%	74	0	0.00%	0	.	.
Kidney renal papillary carcinoma	Kidney	384	0	0.00%	382	0	0.00%	0	.	.
Head and neck odontogenic carcinoma	Bone sarcoma	8	0	0.00%	8	0	0.00%	0	.	.

Disease ontology	Disease group	Disease ontology counts (all)	Count altered in disease ontology (all)	Percent altered in disease ontology (all)	Disease ontology counts (adult)	Count altered in disease ontology (adult)	Percent altered in disease ontology (adult)	Disease ontology counts (paediatric)	Count altered in disease ontology (paediatric)	Percent altered in disease ontology (paediatric)
Unknown primary carcinosarcoma	Unknown primary carcinoma (CUP)	83	0	0.00%	83	0	0.00%	0	.	.
Unknown primary mucoepidermoid carcinoma	Unknown primary carcinoma (CUP)	15	0	0.00%	15	0	0.00%	0	.	.
Breast mucinous carcinoma	Breast	31	0	0.00%	31	0	0.00%	0	.	.
Nasopharynx and paranasal sinuses aesthesioneuroblastoma	Head and neck-neuro	56	0	0.00%	54	0	0.00%	2	0	0.00%
Soft tissue arteriovenous malformation	Soft tissue sarcoma	8	0	0.00%	0	.	.	8	0	0.00%
Lung pleomorphic adenoma	Lung salivary gland-type	6	0	0.00%	6	0	0.00%	0	.	.
Brain gliosis	Benign	8	0	0.00%	7	0	0.00%	1	0	0.00%
Brain germ cell tumour	Germ cell	20	0	0.00%	10	0	0.00%	10	0	0.00%
Head and neck neuroendocrine carcinoma	Head and neck-neuro	91	0	0.00%	89	0	0.00%	1	0	0.00%
Vagina adenocarcinoma	Female genital	63	0	0.00%	61	0	0.00%	2	0	0.00%
Soft tissue lymphangioma	Soft tissue sarcoma	13	0	0.00%	4	0	0.00%	9	0	0.00%
Pericardium mesothelioma	Mesothelioma	10	0	0.00%	10	0	0.00%	0	.	.
Soft tissue ependymoma	Soft tissue sarcoma	9	0	0.00%	7	0	0.00%	2	0	0.00%
Soft tissue leiomyomatosis	Soft tissue sarcoma	14	0	0.00%	14	0	0.00%	0	.	.
Anus squamous cell carcinoma (SCC)	Anus	829	0	0.00%	829	0	0.00%	0	.	.
Soft tissue ganglioneuroma	Soft tissue sarcoma	16	0	0.00%	6	0	0.00%	10	0	0.00%
Bone Ewing sarcoma	Ewing sarcoma	83	0	0.00%	71	0	0.00%	12	0	0.00%
Spleen sarcoma	Soft tissue sarcoma	6	0	0.00%	6	0	0.00%	0	.	.
Spine meningioma	CNS non-glioma	7	0	0.00%	7	0	0.00%	0	.	.
Head and neck lymphoepithelioma	Head and neck	6	0	0.00%	6	0	0.00%	0	.	.
Head and neck squamous papilloma	Head and neck	4	0	0.00%	4	0	0.00%	0	.	.
Brain neurocytoma	CNS non-glioma	24	0	0.00%	17	0	0.00%	7	0	0.00%
Breast inflammatory carcinoma	Breast	33	0	0.00%	32	0	0.00%	0	.	.
Pancreas solid pseudopapillary tumour	Pancreas	36	0	0.00%	26	0	0.00%	10	0	0.00%

Disease ontology	Disease group	Disease ontology counts (all)	Count altered in disease ontology (all)	Percent altered in disease ontology (all)	Disease ontology counts (adult)	Count altered in disease ontology (adult)	Percent altered in disease ontology (adult)	Disease ontology counts (paediatric)	Count altered in disease ontology (paediatric)	Percent altered in disease ontology (paediatric)
Vulva adenoid cystic carcinoma	Adenoid cystic carcinoma	15	0	0.00%	15	0	0.00%	0	.	.
Breast phyllodes tumour	Breast	100	0	0.00%	98	0	0.00%	1	0	0.00%
Stomach leiomyosarcoma	Leiomyosarcoma	21	0	0.00%	21	0	0.00%	0	.	.
Eye lacrimal duct carcinoma	Eye	26	0	0.00%	26	0	0.00%	0	.	.
Uterus perivascular epithelioid cell tumour (PEComa)	Uterus	16	0	0.00%	16	0	0.00%	0	.	.
Head and neck myoepithelial carcinoma	Head and neck	21	0	0.00%	19	0	0.00%	2	0	0.00%
Brain dysembryonic neuroepithelial tumour (DNET)	CNS non-glioma	12	0	0.00%	2	0	0.00%	10	0	0.00%
Appendix adenocarcinoma	Appendix	1228	0	0.00%	1217	0	0.00%	0	.	.
Skin atypical fibroxanthoma	Skin sarcoma	20	0	0.00%	20	0	0.00%	0	.	.
Ovary malignant mixed mesodermal tumour (MMMT)	Malignant mixed mesodermal tumour (MMMT)	7	0	0.00%	7	0	0.00%	0	.	.
Anus adenosquamous carcinoma	Anus	9	0	0.00%	9	0	0.00%	0	.	.
Ovary mucinous carcinoma	Ovary	200	0	0.00%	200	0	0.00%	0	.	.
Skin glomus tumour	Glomus	11	0	0.00%	11	0	0.00%	0	.	.
Unknown primary undifferentiated large cell carcinoma	Unknown primary carcinoma (CUP)	66	0	0.00%	65	0	0.00%	1	0	0.00%
Oesophagus neuroendocrine carcinoma	GI-neuro	100	0	0.00%	100	0	0.00%	0	.	.
Cervix small cell carcinoma	Small cell	60	0	0.00%	60	0	0.00%	0	.	.
Bone odontogenic carcinoma	Bone sarcoma	7	0	0.00%	7	0	0.00%	0	.	.
Bone adamantinoma	Bone sarcoma	11	0	0.00%	9	0	0.00%	2	0	0.00%
Skin appendage tumour	Skin	10	0	0.00%	9	0	0.00%	1	0	0.00%
Skin basal cell carcinoma	Skin	232	0	0.00%	231	0	0.00%	0	.	.
Kidney chromophobe carcinoma	Kidney	100	0	0.00%	99	0	0.00%	1	0	0.00%
Pancreas solid and papillary tumour	Pancreas	9	0	0.00%	8	0	0.00%	1	0	0.00%
Urachus carcinoma	Urinary	84	0	0.00%	84	0	0.00%	0	.	.

Disease ontology	Disease group	Disease ontology counts (all)	Count altered in disease ontology (all)	Percent altered in disease ontology (all)	Disease ontology counts (adult)	Count altered in disease ontology (adult)	Percent altered in disease ontology (adult)	Disease ontology counts (paediatric)	Count altered in disease ontology (paediatric)	Percent altered in disease ontology (paediatric)
Pancreas intraductal papillary mucinous tumour (IPMT)	Pancreas	34	0	0.00%	34	0	0.00%	0	.	.
Prostate undifferentiated carcinoma	Prostate	217	0	0.00%	216	0	0.00%	0	.	.
Peritoneum adenocarcinoma	Peritoneum	130	0	0.00%	130	0	0.00%	0	.	.
Head and neck melanoma	Melanoma	156	0	0.00%	156	0	0.00%	0	.	.
Pleura mesothelioma	Mesothelioma	1045	0	0.00%	1037	0	0.00%	2	0	0.00%
Brain gliosarcoma	Glioma	117	0	0.00%	114	0	0.00%	3	0	0.00%
Vagina melanoma	Melanoma	62	0	0.00%	62	0	0.00%	0	.	.
Pancreas ductal carcinoma	Pancreas	7	0	0.00%	7	0	0.00%	0	.	.
Mesothelioma (NOS)	Mesothelioma	15	0	0.00%	15	0	0.00%	0	.	.
Eye intraocular squamous cell carcinoma (SCC)	Eye	7	0	0.00%	7	0	0.00%	0	.	.
Brain oligoastrocytoma	Glioma	97	0	0.00%	95	0	0.00%	2	0	0.00%
CNS sarcoma (NOS)	CNS sarcoma	10	0	0.00%	7	0	0.00%	3	0	0.00%
Soft tissue rhabdomyosarcoma embryonal	Rhabdomyosarcoma	128	0	0.00%	37	0	0.00%	90	0	0.00%
Unknown primary adenoid cystic carcinoma	Adenoid cystic carcinoma	223	0	0.00%	220	0	0.00%	1	0	0.00%
Soft tissue neurofibroma	Soft tissue sarcoma	47	0	0.00%	35	0	0.00%	12	0	0.00%
Bladder neuroendocrine carcinoma	Urinary-neuro	85	0	0.00%	85	0	0.00%	0	.	.
Thyroid follicular oncocytic carcinoma	Thyroid	51	0	0.00%	51	0	0.00%	0	.	.
Soft tissue rhabdomyosarcoma alveolar	Rhabdomyosarcoma	135	0	0.00%	51	0	0.00%	83	0	0.00%
Soft tissue clear cell sarcoma	Soft tissue sarcoma	94	0	0.00%	79	0	0.00%	14	0	0.00%
Spine ependymoma	CNS non-glioma	62	0	0.00%	47	0	0.00%	15	0	0.00%
Bone osteoblastoma	Benign	5	0	0.00%	4	0	0.00%	1	0	0.00%
Soft tissue haemangioendothelioma	Soft tissue sarcoma	99	0	0.00%	80	0	0.00%	18	0	0.00%
Peritoneum mucinous carcinoma	Peritoneum	16	0	0.00%	16	0	0.00%	0	.	.
Brain craniopharyngioma	CNS non-glioma	50	0	0.00%	42	0	0.00%	8	0	0.00%
Lung pulmonary blastoma	Lung sarcoma	17	0	0.00%	8	0	0.00%	8	0	0.00%

Disease ontology	Disease group	Disease ontology counts (all)	Count altered in disease ontology (all)	Percent altered in disease ontology (all)	Disease ontology counts (adult)	Count altered in disease ontology (adult)	Percent altered in disease ontology (adult)	Disease ontology counts (paediatric)	Count altered in disease ontology (paediatric)	Percent altered in disease ontology (paediatric)
Thymus carcinoma	Thymus	423	0	0.00%	419	0	0.00%	4	0	0.00%
Skin dermatofibrosarcoma	Skin sarcoma	62	0	0.00%	57	0	0.00%	5	0	0.00%
Ovary small cell carcinoma	Small cell	66	0	0.00%	59	0	0.00%	7	0	0.00%
Soft tissue round cell tumour	Soft tissue sarcoma	25	0	0.00%	22	0	0.00%	3	0	0.00%
Skin desmoplastic melanoma	Melanoma	14	0	0.00%	14	0	0.00%	0	.	.
Ureter urothelial carcinoma	Urinary	368	0	0.00%	368	0	0.00%	0	.	.
Ureter adenocarcinoma	Urinary	5	0	0.00%	5	0	0.00%	0	.	.
Thymus neuroendocrine carcinoma	Thymus-neuro	20	0	0.00%	20	0	0.00%	0	.	.
Breast ductal carcinoma in situ (DCIS)	Breast	45	0	0.00%	45	0	0.00%	0	.	.
Soft tissue myoepithelioma	Soft tissue sarcoma	17	0	0.00%	16	0	0.00%	1	0	0.00%
Peritoneum carcinosarcoma	Peritoneum	8	0	0.00%	8	0	0.00%	0	.	.
Peritoneum mesothelioma	Mesothelioma	333	0	0.00%	328	0	0.00%	4	0	0.00%
Testis germ cell tumour (non-seminoma)	Germ cell	190	0	0.00%	181	0	0.00%	7	0	0.00%
Skin melanocytic lesion	Benign	11	0	0.00%	3	0	0.00%	8	0	0.00%
Cervix adenosquamous carcinoma	Cervix	77	0	0.00%	77	0	0.00%	0	.	.
Pituitary adenoma	Pituitary gland	58	0	0.00%	53	0	0.00%	5	0	0.00%
Bladder leiomyosarcoma	Leiomyosarcoma	10	0	0.00%	10	0	0.00%	0	.	.
Brain primitive neuroectoderm tumour (PNET)	CNS non-glioma	49	0	0.00%	22	0	0.00%	27	0	0.00%
Vulva squamous cell carcinoma (SCC)	Female genital	400	0	0.00%	398	0	0.00%	0	.	.
Unknown primary endometrioid carcinoma	Unknown primary carcinoma (CUP)	15	0	0.00%	15	0	0.00%	0	.	.
Soft tissue myofibromatosis	Soft tissue sarcoma	7	0	0.00%	0	.	.	7	0	0.00%
Urethra clear cell carcinoma	Urinary	17	0	0.00%	17	0	0.00%	0	.	.
Soft tissue meningioma	Soft tissue sarcoma	6	0	0.00%	5	0	0.00%	1	0	0.00%
Peritoneum clear cell carcinoma	Peritoneum	15	0	0.00%	15	0	0.00%	0	.	.
Kidney carcinoma (NOS)	Kidney	179	0	0.00%	177	0	0.00%	2	0	0.00%
Kidney sarcomatoid carcinoma	Kidney	157	0	0.00%	154	0	0.00%	1	0	0.00%
Skin Merkel cell carcinoma	Skin-neuro	443	0	0.00%	443	0	0.00%	0	.	.

Disease ontology	Disease group	Disease ontology counts (all)	Count altered in disease ontology (all)	Percent altered in disease ontology (all)	Disease ontology counts (adult)	Count altered in disease ontology (adult)	Percent altered in disease ontology (adult)	Disease ontology counts (paediatric)	Count altered in disease ontology (paediatric)	Percent altered in disease ontology (paediatric)
Testis Leydig cell tumour	Testis	14	0	0.00%	14	0	0.00%	0	.	.
Trachea squamous cell carcinoma (SCC)	Head and neck	15	0	0.00%	15	0	0.00%	0	.	.
Skin extramammary Paget's disease	Skin	42	0	0.00%	42	0	0.00%	0	.	.
Lung typical carcinoid	Carcinoid	79	0	0.00%	79	0	0.00%	0	.	.
Brain subependymoma	CNS non-glioma	7	0	0.00%	7	0	0.00%	0	.	.
Head and neck mucoepidermoid carcinoma	Head and neck	75	0	0.00%	74	0	0.00%	1	0	0.00%
Vulva adenocarcinoma	Female genital	32	0	0.00%	32	0	0.00%	0	.	.
Soft tissue hamartoma	Soft tissue sarcoma	8	0	0.00%	3	0	0.00%	5	0	0.00%
Bone chordoma	Bone sarcoma	246	0	0.00%	232	0	0.00%	12	0	0.00%
Bone giant cell tumour	Bone sarcoma	67	0	0.00%	55	0	0.00%	12	0	0.00%
Bone epithelioid haemangioendothelioma	Bone sarcoma	15	0	0.00%	13	0	0.00%	2	0	0.00%
Unknown primary germ cell tumour	Germ cell	37	0	0.00%	29	0	0.00%	8	0	0.00%
Colon dysplasia	Benign	10	0	0.00%	10	0	0.00%	0	.	.
Kidney malignant rhabdoid tumor (MRT)	Kidney	11	0	0.00%	1	0	0.00%	10	0	0.00%
Brain pineal parenchymal tumour of intermediate differentiation (PPTID)	CNS non-glioma	8	0	0.00%	7	0	0.00%	1	0	0.00%
Breast adenoid cystic carcinoma	Adenoid cystic carcinoma	43	0	0.00%	43	0	0.00%	0	.	.
Appendix mucinous neoplasm	Appendix	210	0	0.00%	210	0	0.00%	0	.	.
Rectum neuroendocrine carcinoma	GI-neuro	75	0	0.00%	75	0	0.00%	0	.	.
Ovary carcinoma mixed histology	Ovary	157	0	0.00%	156	0	0.00%	0	.	.
Colon carcinoid tumour	Carcinoid	32	0	0.00%	32	0	0.00%	0	.	.
Brain pineoblastoma	CNS non-glioma	33	0	0.00%	15	0	0.00%	18	0	0.00%
Breast papillary carcinoma	Breast	11	0	0.00%	11	0	0.00%	0	.	.
Uterus endometrial adenocarcinoma clear cell	Endometrial	366	0	0.00%	365	0	0.00%	0	.	.

Disease ontology	Disease group	Disease ontology counts (all)	Count altered in disease ontology (all)	Percent altered in disease ontology (all)	Disease ontology counts (adult)	Count altered in disease ontology (adult)	Percent altered in disease ontology (adult)	Disease ontology counts (paediatric)	Count altered in disease ontology (paediatric)	Percent altered in disease ontology (paediatric)
Bone chondroblastoma	Bone sarcoma	6	0	0.00%	6	0	0.00%	0	.	.
Soft tissue haemangiopericytoma	Soft tissue sarcoma	71	0	0.00%	68	0	0.00%	3	0	0.00%
Breast myoepithelial carcinoma	Breast	7	0	0.00%	7	0	0.00%	0	.	.
Soft tissue angiomyxoma	Soft tissue sarcoma	8	0	0.00%	7	0	0.00%	1	0	0.00%
Soft tissue low grade fibromyxoid sarcoma	Soft tissue sarcoma	5	0	0.00%	5	0	0.00%	0	.	.
Placenta trophoblastic tumour	Placenta	16	0	0.00%	16	0	0.00%	0	.	.
Urethra urothelial carcinoma	Urinary	58	0	0.00%	58	0	0.00%	0	.	.
Colon GIST	GIST	77	0	0.00%	77	0	0.00%	0	.	.
Ampullary adenocarcinoma	Biliary	518	0	0.00%	518	0	0.00%	0	.	.
Breast lobular carcinoma in situ	Breast	16	0	0.00%	16	0	0.00%	0	.	.
Neuroblastoma	Peripheral nervous system (PNS)	483	0	0.00%	66	0	0.00%	412	0	0.00%
Lung sarcoma (NOS)	Lung sarcoma	42	0	0.00%	42	0	0.00%	0	.	.
Trachea adenoid cystic carcinoma	Adenoid cystic carcinoma	17	0	0.00%	17	0	0.00%	0	.	.
Gallbladder adenosquamous carcinoma	Biliary	36	0	0.00%	36	0	0.00%	0	.	.
Oesophagus adenosquamous carcinoma	Oesophagus	16	0	0.00%	16	0	0.00%	0	.	.
Uterus smooth muscle tumour of uncertain malignant potential (STUMP)	Uterus sarcoma	9	0	0.00%	9	0	0.00%	0	.	.

CNS, central nervous system; FLO, fibrolamellar oncocytic variant; GI, gastrointestinal; GIST, gastrointestinal stromal tumour; LMP, low malignant potential; NOS, not otherwise specified; NTRK, neurotrophic tyrosine receptor kinase; NUT, nuclear protein in testis.

Supplementary Table 3. Prevalence of *NTRK* fusion-positive specimens in FoundationCORE by age group

Age bucket	Total in age bucket	Count *NTRK* + in age bucket	Percent *NTRK* + in age bucket
>=0 and <5	1227	28	2.28%
>=5 and <10	1030	11	1.07%
>=10 and <15	1189	11	0.93%
>=15 and <20	1519	13	0.86%
>=20 and <25	1934	12	0.62%
>=25 and <30	3199	14	0.44%
>=30 and <35	5451	25	0.46%
>=35 and <40	8608	30	0.35%
>=40 and <45	12582	36	0.29%
>=45 and <50	19678	65	0.33%
>=50 and <55	28962	70	0.24%
>=55 and <60	39287	110	0.28%
>=60 and <65	45041	111	0.25%
>=65 and <70	45187	140	0.31%
>=70 and <75	36487	97	0.27%
>=75 and <80	24319	56	0.23%
>=80 and <85	12633	37	0.29%
>=85	6486	20	0.31%

Supplementary Table 4. Prevalence of *NTRK* fusion-positive specimens by disease ontology in paediatric age groups

Disease ontology	≥0 and <5 (*n* = 1227 total samples)	≥5 and <10 (*n* = 1030 total samples)	≥10 and <15 (*N* = 1189 total samples)	≥15 and <18 (*n* = 942 total samples)
Soft tissue fibrosarcoma	1.06%	0%	0%	0%
Brain glioma (NOS)	0.24%	0%	0%	0%
Soft tissue malignant peripheral nerve sheath tumour (MPNST)	0.16%	0%	0%	0%
Soft tissue sarcoma (NOS)	0.16%	0.29%	0.08%	0%
Bone chondrosarcoma	0.08%	0%	0%	0%
Brain astrocytoma	0.08%	0%	0%	0%
Brain astrocytoma pilocytic	0.08%	0.10%	0%	0.11%
Brain glioblastoma (GBM)	0.08%	0.10%	0%	0.11%
Schwannoma	0.08%	0%	0%	0%
Soft tissue haemangioma	0.08%	0%	0%	0%
Spine glioma (NOS)	0.08%	0%	0%	0%
Unknown primary melanoma	0.08%	0%	0%	0%
Bone osteosarcoma	0%	0.10%	0.08%	0%
Brain pleomorphic xanthoastrocytoma	0%	0.10%	0%	0%
Soft tissue sarcoma undifferentiated	0%	0.10%	0%	0%
Thyroid papillary carcinoma	0%	0.29%	0.34%	0.21%
Adrenal gland neuroblastoma	0%	0%	0.08%	0%
Brain medulloblastoma	0%	0%	0.08%	0%
Salivary gland adenocarcinoma	0%	0%	0.08%	0%
Salivary gland mammary analogue secretory carcinoma (MASC)	0%	0%	0.08%	0.11%
Soft tissue primitive neuroectoderm tumour (PNET)	0%	0%	0.08%	0%
Breast carcinoma (NOS)	0%	0%	0%	0.11%
Rhabdomyosarcoma (NOS)	0%	0%	0%	0.11%
Soft tissue inflammatory myofibroblastic tumour	0%	0%	0%	0.11%
Soft tissue solitary fibrous tumour	0%	0%	0%	0.11%

NOS, not otherwise specified; *NTRK*, neurotrophic tyrosine receptor kinase.

Supplementary Table 5. Expected *NTRK* fusion detection rates on FoundationOne CDx

An assessment of *NTRK* fusion detection rate was projected for the FoundationOne CDx platform using COSMIC (cancer.sanger.ac.uk) as the reference baseline and equation (1).

Fusion pair	Total # in COSMIC	Estimated detection
TPM3:NTRK1	38	100%
TPR:NTRK1 variant I	2	100%
TPR:NTRK1 variant II	2	96.6%
TFG:NTRK1	1	100%
TFG:NTRK1	1	100%
LMNA:NTRK1	2	100%
TP53:NTRK1	1	100%
QKI:NTRK2	2	48.9%
NACC2:NTRK2	1	100%
ETV6:NTRK3 variant I	119	100%
ETV6:NTRK3 variant II	15	0.4%
ETV6:NTRK3 variant III	1	100%
ETV6:NTRK3 variant IV	1	100%
**Overall F1CDx detection rate of *NTRK* fusions**		**90.9%**

Supplementary Table 6. *NTRK* gene fusions detected among adult patients

Partner	*NTRK*	Count *NTRK* of interest partnered	Percent *NTRK* of interest partnered	Count any *NTRK* partnered
PCSK5	*NTRK1*	0	0.00%	1
TNRC6A	*NTRK1*	0	0.00%	1
SEMA4B	*NTRK1*	1	0.34%	1
SMG5	*NTRK1*	1	0.34%	1
CCDC88C	*NTRK1*	1	0.34%	1
ARHGEF2	*NTRK1*	1	0.34%	1
RORA	*NTRK1*	0	0.00%	1
IRF2BP2	*NTRK1*	6	2.03%	6
ARHGEF11	*NTRK1*	3	1.02%	3
CHTOP	*NTRK1*	1	0.34%	1
BGLAP	*NTRK1*	1	0.34%	1
TPR	*NTRK1*	17	5.76%	17
KIRREL1	*NTRK1*	2	0.68%	2
MDM4	*NTRK1*	1	0.34%	1
LRRC71	*NTRK1*	3	1.02%	3
EFL1	*NTRK1*	0	0.00%	1
PLEKHA6	*NTRK1*	3	1.02%	3
RUNX1	*NTRK1*	0	0.00%	1
AMMECR1	*NTRK1*	0	0.00%	1
PRRX1	*NTRK1*	0	0.00%	1
GON4L	*NTRK1*	1	0.34%	1
TRIM33	*NTRK1*	1	0.34%	1
SPECC1L	*NTRK1*	0	0.00%	1
ZSCAN2	*NTRK1*	0	0.00%	1
LRRK1	*NTRK1*	0	0.00%	1
CDC42BPA	*NTRK1*	1	0.34%	1
THADA	*NTRK1*	0	0.00%	1
DLG1	*NTRK1*	0	0.00%	1
NFASC	*NTRK1*	1	0.34%	1
ACO1	*NTRK1*	1	0.34%	2
LINC00924	*NTRK1*	0	0.00%	1
BCAN	*NTRK1*	4	1.36%	4
TP53	*NTRK1*	1	0.34%	1
GNAQ	*NTRK1*	0	0.00%	1
ETV6	*NTRK1*	0	0.00%	78
SFPQ	*NTRK1*	1	0.34%	1
MEF2A	*NTRK1*	0	0.00%	1
DCST1	*NTRK1*	1	0.34%	1
NAB2	*NTRK1*	1	0.34%	1
ARGLU1	*NTRK1*	1	0.34%	1
AKAP13	*NTRK1*	0	0.00%	1
LMNA	*NTRK1*	30	10.17%	30
STRN3	*NTRK1*	0	0.00%	3
CGN	*NTRK1*	1	0.34%	1
TPM4	*NTRK1*	0	0.00%	1
TPM3	*NTRK1*	65	22.03%	65
TPM1	*NTRK1*	1	0.34%	1
CTDSP2	*NTRK1*	0	0.00%	1
SEL1L	*NTRK1*	1	0.34%	1
NLGN1	*NTRK1*	1	0.34%	1
EML4	*NTRK1*	1	0.34%	3
EFNA3	*NTRK1*	1	0.34%	1
GRIPAP1	*NTRK1*	1	0.34%	1
PRKAR1A	*NTRK1*	1	0.34%	1
PIP5K1A	*NTRK1*	1	0.34%	1
PAIP1	*NTRK1*	0	0.00%	1
RAD52	*NTRK1*	0	0.00%	1
GATAD2B	*NTRK1*	2	0.68%	2
FAM117B	*NTRK1*	0	0.00%	1

ERC1	*NTRK1*	2	0.68%	2
RCSD1	*NTRK1*	1	0.34%	1
IQGAP1	*NTRK1*	0	0.00%	1
EPS15	*NTRK1*	1	0.34%	1
CDK12	*NTRK1*	0	0.00%	1
PEAR1	*NTRK1*	5	1.69%	5
NOD1	*NTRK1*	0	0.00%	1
CABLES1	*NTRK1*	1	0.34%	1
ZBTB1	*NTRK1*	1	0.34%	1
DAB2IP	*NTRK1*	0	0.00%	1
PPP6R3	*NTRK1*	0	0.00%	1
DENND1A	*NTRK1*	0	0.00%	1
TRIP13	*NTRK1*	0	0.00%	1
DUSP10	*NTRK1*	1	0.34%	1
MEX3A	*NTRK1*	1	0.34%	1
CHST11	*NTRK1*	0	0.00%	1
SQSTM1	*NTRK1*	1	0.34%	3
RAB25	*NTRK1*	1	0.34%	1
TFG	*NTRK1*	2	0.68%	2
CUL4A	*NTRK1*	1	0.34%	1
PTPRC	*NTRK1*	1	0.34%	1
PCSK5	*NTRK2*	1	0.34%	1
TNRC6A	*NTRK2*	0	0.00%	1
SEMA4B	*NTRK2*	0	0.00%	1
SMG5	*NTRK2*	0	0.00%	1
CCDC88C	*NTRK2*	0	0.00%	1
ARHGEF2	*NTRK2*	0	0.00%	1
RORA	*NTRK2*	0	0.00%	1
IRF2BP2	*NTRK2*	0	0.00%	6
ARHGEF11	*NTRK2*	0	0.00%	3
CHTOP	*NTRK2*	0	0.00%	1
BGLAP	*NTRK2*	0	0.00%	1
TPR	*NTRK2*	0	0.00%	17
KIRREL1	*NTRK2*	0	0.00%	2
MDM4	*NTRK2*	0	0.00%	1
LRRC71	*NTRK2*	0	0.00%	3
EFL1	*NTRK2*	0	0.00%	1
PLEKHA6	*NTRK2*	0	0.00%	3
RUNX1	*NTRK2*	0	0.00%	1
AMMECR1	*NTRK2*	0	0.00%	1
PRRX1	*NTRK2*	1	0.34%	1
GON4L	*NTRK2*	0	0.00%	1
TRIM33	*NTRK2*	0	0.00%	1
SPECC1L	*NTRK2*	0	0.00%	1
ZSCAN2	*NTRK2*	0	0.00%	1
LRRK1	*NTRK2*	0	0.00%	1
CDC42BPA	*NTRK2*	0	0.00%	1
THADA	*NTRK2*	1	0.34%	1
DLG1	*NTRK2*	0	0.00%	1
NFASC	*NTRK2*	0	0.00%	1
ACO1	*NTRK2*	1	0.34%	2
LINC00924	*NTRK2*	0	0.00%	1
BCAN	*NTRK2*	0	0.00%	4
TP53	*NTRK2*	0	0.00%	1
GNAQ	*NTRK2*	1	0.34%	1
ETV6	*NTRK2*	0	0.00%	78
SFPQ	*NTRK2*	0	0.00%	1
MEF2A	*NTRK2*	0	0.00%	1
DCST1	*NTRK2*	0	0.00%	1
NAB2	*NTRK2*	0	0.00%	1
ARGLU1	*NTRK2*	0	0.00%	1
AKAP13	*NTRK2*	0	0.00%	1

LMNA	*NTRK2*	0	0.00%	30
STRN3	*NTRK2*	0	0.00%	3
CGN	*NTRK2*	0	0.00%	1
TPM4	*NTRK2*	0	0.00%	1
TPM3	*NTRK2*	0	0.00%	65
TPM1	*NTRK2*	0	0.00%	1
CTDSP2	*NTRK2*	1	0.34%	1
SEL1L	*NTRK2*	0	0.00%	1
NLGN1	*NTRK2*	0	0.00%	1
EML4	*NTRK2*	0	0.00%	3
EFNA3	*NTRK2*	0	0.00%	1
GRIPAP1	*NTRK2*	0	0.00%	1
PRKAR1A	*NTRK2*	0	0.00%	1
PIP5K1A	*NTRK2*	0	0.00%	1
PAIP1	*NTRK2*	1	0.34%	1
RAD52	*NTRK2*	0	0.00%	1
GATAD2B	*NTRK2*	0	0.00%	2
FAM117B	*NTRK2*	1	0.34%	1
ERC1	*NTRK2*	0	0.00%	2
RCSD1	*NTRK2*	0	0.00%	1
IQGAP1	*NTRK2*	0	0.00%	1
EPS15	*NTRK2*	0	0.00%	1
CDK12	*NTRK2*	0	0.00%	1
PEAR1	*NTRK2*	0	0.00%	5
NOD1	*NTRK2*	1	0.34%	1
CABLES1	*NTRK2*	0	0.00%	1
ZBTB1	*NTRK2*	0	0.00%	1
DAB2IP	*NTRK2*	1	0.34%	1
PPP6R3	*NTRK2*	1	0.34%	1
DENND1A	*NTRK2*	1	0.34%	1
TRIP13	*NTRK2*	1	0.34%	1
DUSP10	*NTRK2*	0	0.00%	1
MEX3A	*NTRK2*	0	0.00%	1
CHST11	*NTRK2*	0	0.00%	1
SQSTM1	*NTRK2*	1	0.34%	3
RAB25	*NTRK2*	0	0.00%	1
TFG	*NTRK2*	0	0.00%	2
CUL4A	*NTRK2*	0	0.00%	1
PTPRC	*NTRK2*	0	0.00%	1
PCSK5	*NTRK3*	0	0.00%	1
TNRC6A	*NTRK3*	1	0.34%	1
SEMA4B	*NTRK3*	0	0.00%	1
SMG5	*NTRK3*	0	0.00%	1
CCDC88C	*NTRK3*	0	0.00%	1
ARHGEF2	*NTRK3*	0	0.00%	1
RORA	*NTRK3*	1	0.34%	1
IRF2BP2	*NTRK3*	0	0.00%	6
ARHGEF11	*NTRK3*	0	0.00%	3
CHTOP	*NTRK3*	0	0.00%	1
BGLAP	*NTRK3*	0	0.00%	1
TPR	*NTRK3*	0	0.00%	17
KIRREL1	*NTRK3*	0	0.00%	2
MDM4	*NTRK3*	0	0.00%	1
LRRC71	*NTRK3*	0	0.00%	3
EFL1	*NTRK3*	1	0.34%	1
PLEKHA6	*NTRK3*	0	0.00%	3
RUNX1	*NTRK3*	1	0.34%	1
AMMECR1	*NTRK3*	1	0.34%	1
PRRX1	*NTRK3*	0	0.00%	1
GON4L	*NTRK3*	0	0.00%	1
TRIM33	*NTRK3*	0	0.00%	1
SPECC1L	*NTRK3*	1	0.34%	1

ZSCAN2	*NTRK3*	1	0.34%	1
LRRK1	*NTRK3*	1	0.34%	1
CDC42BPA	*NTRK3*	0	0.00%	1
THADA	*NTRK3*	0	0.00%	1
DLG1	*NTRK3*	1	0.34%	1
NFASC	*NTRK3*	0	0.00%	1
ACO1	*NTRK3*	0	0.00%	2
LINC00924	*NTRK3*	1	0.34%	1
BCAN	*NTRK3*	0	0.00%	4
TP53	*NTRK3*	0	0.00%	1
GNAQ	*NTRK3*	0	0.00%	1
ETV6	*NTRK3*	78	26.44%	78
SFPQ	*NTRK3*	0	0.00%	1
MEF2A	*NTRK3*	1	0.34%	1
DCST1	*NTRK3*	0	0.00%	1
NAB2	*NTRK3*	0	0.00%	1
ARGLU1	*NTRK3*	0	0.00%	1
AKAP13	*NTRK3*	1	0.34%	1
LMNA	*NTRK3*	0	0.00%	30
STRN3	*NTRK3*	3	1.02%	3
CGN	*NTRK3*	0	0.00%	1
TPM4	*NTRK3*	1	0.34%	1
TPM3	*NTRK3*	0	0.00%	65
TPM1	*NTRK3*	0	0.00%	1
CTDSP2	*NTRK3*	0	0.00%	1
SEL1L	*NTRK3*	0	0.00%	1
NLGN1	*NTRK3*	0	0.00%	1
EML4	*NTRK3*	2	0.68%	3
EFNA3	*NTRK3*	0	0.00%	1
GRIPAP1	*NTRK3*	0	0.00%	1
PRKAR1A	*NTRK3*	0	0.00%	1
PIP5K1A	*NTRK3*	0	0.00%	1
PAIP1	*NTRK3*	0	0.00%	1
RAD52	*NTRK3*	1	0.34%	1
GATAD2B	*NTRK3*	0	0.00%	2
FAM117B	*NTRK3*	0	0.00%	1
ERC1	*NTRK3*	0	0.00%	2
RCSD1	*NTRK3*	0	0.00%	1
IQGAP1	*NTRK3*	1	0.34%	1
EPS15	*NTRK3*	0	0.00%	1
CDK12	*NTRK3*	1	0.34%	1
PEAR1	*NTRK3*	0	0.00%	5
NOD1	*NTRK3*	0	0.00%	1
CABLES1	*NTRK3*	0	0.00%	1
ZBTB1	*NTRK3*	0	0.00%	1
DAB2IP	*NTRK3*	0	0.00%	1
PPP6R3	*NTRK3*	0	0.00%	1
DENND1A	*NTRK3*	0	0.00%	1
TRIP13	*NTRK3*	0	0.00%	1
DUSP10	*NTRK3*	0	0.00%	1
MEX3A	*NTRK3*	0	0.00%	1
CHST11	*NTRK3*	1	0.34%	1
SQSTM1	*NTRK3*	1	0.34%	3
RAB25	*NTRK3*	0	0.00%	1
TFG	*NTRK3*	0	0.00%	2
CUL4A	*NTRK3*	0	0.00%	1
PTPRC	*NTRK3*	0	0.00%	1

Supplementary Table 7. ***NTRK*** **gene fusions detected among paediatric patients**

**Partner**	***NTRK***	**Count *NTRK* of interest partnered**	**Percent *NTRK* of interest partnered**	**Count any *NTRK* partnered**
IRF2BP2	*NTRK1*	1	1.92%	1
TPR	*NTRK1*	6	11.54%	6
SPECC1L	*NTRK1*	0	0.00%	1
PKM	*NTRK1*	0	0.00%	1
ETV6	*NTRK1*	0	0.00%	17
STRN	*NTRK1*	0	0.00%	1
LMNA	*NTRK1*	3	5.77%	3
BCR	*NTRK1*	0	0.00%	1
TPM3	*NTRK1*	10	19.23%	10
KIF21B	*NTRK1*	1	1.92%	1
EML4	*NTRK1*	0	0.00%	1
PDE4DIP	*NTRK1*	1	1.92%	1
AFAP1	*NTRK1*	0	0.00%	1
MTA1	*NTRK1*	1	1.92%	1
SQSTM1	*NTRK1*	3	5.77%	4
HMBOX1	*NTRK1*	0	0.00%	1
TFG	*NTRK1*	0	0.00%	1
IRF2BP2	*NTRK2*	0	0.00%	1
TPR	*NTRK2*	0	0.00%	6
SPECC1L	*NTRK2*	1	1.92%	1
PKM	*NTRK2*	0	0.00%	1
ETV6	*NTRK2*	0	0.00%	17
STRN	*NTRK2*	1	1.92%	1
LMNA	*NTRK2*	0	0.00%	3
BCR	*NTRK2*	1	1.92%	1
TPM3	*NTRK2*	0	0.00%	10
KIF21B	*NTRK2*	0	0.00%	1
EML4	*NTRK2*	0	0.00%	1
PDE4DIP	*NTRK2*	0	0.00%	1
AFAP1	*NTRK2*	1	1.92%	1
MTA1	*NTRK2*	0	0.00%	1
SQSTM1	*NTRK2*	0	0.00%	4
HMBOX1	*NTRK2*	0	0.00%	1
TFG	*NTRK2*	0	0.00%	1
IRF2BP2	*NTRK3*	0	0.00%	1
TPR	*NTRK3*	0	0.00%	6
SPECC1L	*NTRK3*	0	0.00%	1
PKM	*NTRK3*	1	1.92%	1
ETV6	*NTRK3*	17	32.69%	17
STRN	*NTRK3*	0	0.00%	1
LMNA	*NTRK3*	0	0.00%	3
BCR	*NTRK3*	0	0.00%	1
TPM3	*NTRK3*	0	0.00%	10
KIF21B	*NTRK3*	0	0.00%	1
EML4	*NTRK3*	1	1.92%	1
PDE4DIP	*NTRK3*	0	0.00%	1
AFAP1	*NTRK3*	0	0.00%	1
MTA1	*NTRK3*	0	0.00%	1
SQSTM1	*NTRK3*	1	1.92%	4
HMBOX1	*NTRK3*	1	1.92%	1
TFG	*NTRK3*	1	1.92%	1

Supplementary Table 8. Disease group breakdown of all specimens and *NTRK* fusion-positive specimens by predicted ancestry

Disease group	% of all AFR samples (*n* = 26 666)	% of *NTRK*+ AFR samples (*n* = 90)	% of all AMR samples (*n* = 27 626)	% of *NTRK*+ AMR samples (*n* = 102)	% of all EAS samples (*n* = 13 934)	% of *NTRK*+ EAS samples (*n* = 56)	% of all EUR samples (*n* = 223 448)	% of *NTRK*+ EUR samples (*n* = 625)	% of all SAS samples (*n* = 4002)	% of *NTRK*+ SAS samples (*n* = 16)
Adenoid cystic carcinoma	0.6%	0.0%	0.6%	0.0%	0.7%	0.0%	0.4%	0.0%	0.8%	0.0%
Adrenal gland	0.1%	2.2%	0.3%	0.0%	0.2%	0.0%	0.2%	0.2%	0.2%	0.0%
Angiosarcoma	0.1%	0.0%	0.2%	0.0%	0.2%	0.0%	0.2%	0.5%	0.3%	0.0%
Anus	0.3%	0.0%	0.3%	0.0%	0.0%	0.0%	0.3%	0.0%	0.0%	0.0%
Appendix	0.5%	0.0%	0.4%	0.0%	0.4%	0.0%	0.5%	0.0%	0.5%	0.0%
Benign	0.0%	0.0%	0.0%	0.0%	0.0%	0.0%	0.0%	0.0%	0.0%	0.0%
Biliary	1.3%	0.0%	1.5%	2.9%	1.8%	0.0%	0.9%	0.6%	2.4%	0.0%
Bladder	1.3%	1.1%	1.2%	2.9%	1.1%	0.0%	2.0%	2.2%	1.4%	0.0%
Bone sarcoma	0.4%	2.2%	0.8%	2.0%	0.4%	1.8%	0.4%	0.3%	0.7%	0.0%
Breast	14.2%	17.8%	11.4%	7.8%	8.6%	8.9%	9.7%	13.9%	11.5%	12.5%
Carcinoid	0.3%	0.0%	0.2%	0.0%	0.1%	0.0%	0.3%	0.0%	0.2%	0.0%
Cervix	1.0%	0.0%	1.4%	0.0%	1.1%	0.0%	0.7%	0.6%	0.7%	0.0%
Cholangiocarcinoma	1.3%	0.0%	2.3%	0.0%	2.6%	1.8%	1.8%	1.6%	2.2%	0.0%
Chondrosarcoma	0.1%	0.0%	0.3%	1.0%	0.1%	0.0%	0.2%	0.2%	0.3%	0.0%
CNS non-glioma	0.7%	0.0%	1.3%	0.0%	0.7%	0.0%	0.6%	0.2%	0.8%	0.0%
CNS sarcoma	0.0%	0.0%	0.0%	0.0%	0.0%	0.0%	0.0%	0.0%	0.0%	0.0%
Colorectal (CRC)	14.2%	7.8%	12.9%	5.9%	12.4%	7.1%	11.3%	9.4%	9.2%	6.3%
Endocrine-neuro	0.5%	1.1%	0.6%	0.0%	0.4%	0.0%	0.5%	0.3%	0.6%	0.0%
Endometrial	4.0%	3.3%	2.7%	2.0%	2.7%	1.8%	2.6%	1.4%	3.1%	6.3%
Oesophagus	1.4%	1.1%	1.6%	0.0%	1.4%	0.0%	2.9%	2.7%	2.1%	0.0%
Ewing sarcoma	0.1%	0.0%	0.3%	1.0%	0.1%	0.0%	0.2%	0.0%	0.6%	0.0%
Eye	0.0%	0.0%	0.0%	0.0%	0.0%	0.0%	0.0%	0.0%	0.0%	0.0%
Fallopian tube	0.3%	0.0%	0.4%	0.0%	0.4%	0.0%	0.6%	0.8%	0.5%	0.0%
Female genital	0.2%	0.0%	0.2%	0.0%	0.1%	0.0%	0.2%	0.0%	0.1%	0.0%
Female-neuro	0.1%	0.0%	0.2%	0.0%	0.2%	0.0%	0.1%	0.2%	0.1%	0.0%
Germ cell	0.1%	0.0%	0.5%	0.0%	0.2%	0.0%	0.1%	0.0%	0.4%	0.0%
GI-neuro	0.3%	0.0%	0.2%	0.0%	0.1%	0.0%	0.3%	0.2%	0.4%	0.0%
GIST	0.8%	0.0%	0.6%	0.0%	0.8%	1.8%	0.4%	1.1%	0.5%	0.0%
Glioma	1.9%	3.3%	4.6%	6.9%	2.5%	0.0%	3.9%	5.4%	5.6%	12.5%
Glomus	0.0%	0.0%	0.0%	0.0%	0.0%	0.0%	0.0%	0.0%	0.0%	0.0%
Head and neck	1.3%	1.1%	1.3%	0.0%	1.6%	1.8%	1.7%	1.1%	3.0%	0.0%
Head and neck-neuro	0.1%	0.0%	0.1%	0.0%	0.1%	0.0%	0.1%	0.0%	0.0%	0.0%
Heart sarcoma	0.0%	0.0%	0.0%	0.0%	0.0%	0.0%	0.0%	0.0%	0.0%	0.0%

Disease group	% of all AFR samples (*n* = 26 666)	% of *NTRK*+ AFR samples (*n* = 90)	% of all AMR samples (*n* = 27 626)	% of *NTRK*+ AMR samples (*n* = 102)	% of all EAS samples (*n* = 13 934)	% of *NTRK*+ EAS samples (*n* = 56)	% of all EUR samples (*n* = 223 448)	% of *NTRK*+ EUR samples (*n* = 625)	% of all SAS samples (*n* = 4002)	% of *NTRK*+ SAS samples (*n* = 16)
Kaposi sarcoma	0.0%	0.0%	0.0%	0.0%	0.0%	0.0%	0.0%	0.0%	0.0%	0.0%
Kidney	1.6%	1.1%	2.0%	0.0%	1.4%	1.8%	1.8%	0.2%	1.6%	0.0%
Kidney sarcoma	0.0%	0.0%	0.0%	0.0%	0.0%	0.0%	0.0%	0.0%	0.0%	0.0%
Leiomyosarcoma	1.3%	2.2%	1.2%	1.0%	1.0%	7.1%	0.8%	0.8%	1.3%	6.3%
Liver	0.7%	1.1%	0.9%	0.0%	1.3%	0.0%	0.5%	0.0%	1.0%	0.0%
Liver sarcoma	0.0%	0.0%	0.0%	0.0%	0.0%	0.0%	0.0%	0.0%	0.0%	0.0%
Non-small cell lung carcinoma (NSCLC)	18.9%	16.7%	13.0%	10.8%	24.1%	12.5%	19.7%	16.3%	13.8%	6.3%
Lung salivary gland-type	0.0%	0.0%	0.0%	0.0%	0.0%	0.0%	0.0%	0.0%	0.0%	0.0%
Lung sarcoma	0.0%	0.0%	0.0%	0.0%	0.0%	0.0%	0.0%	0.0%	0.0%	0.0%
Male genital	0.1%	0.0%	0.1%	0.0%	0.1%	0.0%	0.1%	0.0%	0.0%	0.0%
Male-neuro	0.1%	0.0%	0.1%	0.0%	0.1%	0.0%	0.1%	0.2%	0.2%	0.0%
Malignant mixed mesodermal tumour (MMMT)	0.0%	0.0%	0.0%	0.0%	0.0%	0.0%	0.0%	0.0%	0.0%	0.0%
Melanoma	0.4%	0.0%	1.6%	2.9%	0.8%	1.8%	3.3%	2.6%	0.8%	0.0%
Mesothelioma	0.2%	0.0%	0.5%	0.0%	0.2%	0.0%	0.5%	0.0%	0.3%	0.0%
Ovary	3.7%	1.1%	5.7%	6.9%	5.6%	5.4%	5.8%	6.2%	6.6%	6.3%
Pancreas	4.7%	1.1%	5.1%	2.0%	5.4%	3.6%	5.9%	3.7%	4.5%	0.0%
Peripheral nervous system (PNS)	0.3%	0.0%	0.4%	0.0%	0.2%	0.0%	0.2%	0.2%	0.3%	0.0%
Peritoneum	0.2%	0.0%	0.3%	0.0%	0.2%	0.0%	0.3%	0.6%	0.4%	0.0%
Pineal gland	0.0%	0.0%	0.0%	0.0%	0.0%	0.0%	0.0%	0.0%	0.0%	0.0%
Pituitary gland	0.0%	0.0%	0.0%	0.0%	0.0%	0.0%	0.0%	0.0%	0.0%	0.0%
Placenta	0.0%	0.0%	0.0%	0.0%	0.0%	0.0%	0.0%	0.0%	0.0%	0.0%
Prostate	4.0%	5.6%	2.4%	2.9%	1.7%	3.6%	3.3%	1.6%	2.6%	6.3%
Rhabdomyosarcoma	0.3%	1.1%	0.4%	0.0%	0.3%	0.0%	0.2%	0.2%	0.3%	6.3%
Salivary gland	0.5%	6.7%	0.4%	2.9%	0.5%	7.1%	0.5%	3.8%	0.5%	6.3%
Skin	0.2%	0.0%	0.2%	0.0%	0.2%	0.0%	0.5%	0.2%	0.3%	0.0%
Skin sarcoma	0.0%	0.0%	0.1%	0.0%	0.0%	0.0%	0.0%	0.0%	0.0%	0.0%
Skin-neuro	0.0%	0.0%	0.1%	0.0%	0.0%	0.0%	0.2%	0.0%	0.0%	0.0%
Small cell	0.9%	0.0%	0.7%	0.0%	0.7%	0.0%	1.3%	0.3%	0.7%	0.0%
Small intestine	0.9%	0.0%	0.4%	0.0%	0.5%	0.0%	0.5%	0.3%	0.5%	0.0%
Soft tissue sarcoma	2.5%	11.1%	3.8%	23.5%	2.4%	12.5%	2.1%	9.8%	3.5%	12.5%
Solitary fibrous tumour	0.1%	0.0%	0.1%	0.0%	0.1%	0.0%	0.1%	0.2%	0.1%	0.0%
Stomach	2.3%	3.3%	3.5%	1.0%	4.6%	3.6%	1.2%	0.3%	3.2%	0.0%

Disease group	% of all AFR samples (*n* = 26 666)	% of *NTRK* + AFR samples (*n* = 90)	% of all AMR samples (*n* = 27 626)	% of *NTRK* + AMR samples (*n* = 102)	% of all EAS samples (*n* = 13 934)	% of *NTRK* + EAS samples (*n* = 56)	% of all EUR samples (*n* = 223 448)	% of *NTRK* + EUR samples (*n* = 625)	% of all SAS samples (*n* = 4002)	% of *NTRK* + SAS samples (*n* = 16)
Testis	0.0%	0.0%	0.0%	0.0%	0.0%	0.0%	0.0%	0.0%	0.0%	0.0%
Thymus	0.2%	0.0%	0.2%	0.0%	0.5%	0.0%	0.1%	0.0%	0.1%	0.0%
Thymus-neuro	0.0%	0.0%	0.0%	0.0%	0.0%	0.0%	0.0%	0.0%	0.0%	0.0%
Thyroid	0.6%	4.4%	1.3%	6.9%	1.0%	8.9%	0.7%	3.4%	1.0%	6.3%
Unknown primary carcinoma (CUP)	5.1%	2.2%	5.0%	5.9%	4.5%	3.6%	5.4%	4.8%	5.5%	0.0%
Unknown primary-neuro	0.8%	0.0%	0.6%	0.0%	0.5%	0.0%	0.7%	0.6%	0.6%	0.0%
Urinary	0.2%	0.0%	0.1%	0.0%	0.3%	0.0%	0.2%	0.0%	0.2%	0.0%
Urinary-neuro	0.0%	0.0%	0.0%	0.0%	0.0%	0.0%	0.0%	0.0%	0.0%	0.0%
Uterus	1.2%	1.1%	0.5%	1.0%	0.5%	1.8%	0.4%	0.3%	0.6%	0.0%
Uterus sarcoma	0.2%	0.0%	0.2%	0.0%	0.2%	1.8%	0.1%	0.5%	0.3%	6.3%

CNS, central nervous system; GI, gastrointestinal; GIST, gastrointestinal stromal tumour; *NTRK*, neurotrophic tyrosine receptor kinase.

Supplementary Table 9. Co-occurrence and mutual exclusivity of altered genes with *NTRK* fusions in solid tumours

Gene	Percent altered in *NTRK+*	Percent altered in *NTRK–*	Odds ratio	Corrected *P*-value (FDR)
ETV6	11.02%	0.24%	51.53421537	1.3312E-120
KRAS	10.69%	22.72%	0.406911397	2.93919E-18
RNF43	5.62%	1.79%	3.271417614	3.20034E-10
APC	5.74%	12.26%	0.435447595	6.43496E-09
IGF1R	3.04%	0.75%	4.127897097	1.21936E-07
CDK4	5.85%	2.36%	2.572999493	2.22685E-07
PIK3CA	8.21%	13.89%	0.554446732	7.79997E-06
MDM2	6.86%	3.37%	2.111617269	9.9645E-06
MDM4	2.81%	1.02%	2.817149711	0.000221471
JUN	1.57%	0.38%	4.237013526	0.000267271
CASP8	2.59%	0.92%	2.871004371	0.000300534
TP53	50.06%	57.28%	0.747531193	0.000310411
PTCH1	2.59%	1.05%	2.499809594	0.001948781
CDKN2B	16.87%	12.67%	1.39958828	0.004602757
ATRX	4.16%	2.19%	1.940730064	0.004895141
CDK12	2.59%	1.13%	2.315414167	0.005121979
SMAD4	3.04%	5.44%	0.544868915	0.014530335
MCL1	4.50%	2.62%	1.754035926	0.01892261
CD79A	0.56%	0.10%	5.824229504	0.022639011
GID4	1.24%	0.43%	2.907021888	0.022639011
KMT2D	7.31%	4.94%	1.518220137	0.022639011
BCR	0.34%	0.03%	11.4696038	0.028105052
BRAF	3.04%	5.15%	0.577303657	0.030197147
CYP17A1	0.34%	0.04%	8.908668575	0.051227073
NBN	1.24%	0.50%	2.515350518	0.05399935
MSH6	2.02%	1.01%	2.028455873	0.055966453
ASXL1	3.15%	1.94%	1.640236455	0.11911041
FGF6	2.47%	1.40%	1.787122751	0.11911041
PARP1	1.35%	0.62%	2.205004309	0.11911041
NRAS	1.12%	2.27%	0.489319652	0.132640991
VHL	0.34%	1.15%	0.290187558	0.132640991
CCND2	2.47%	1.49%	1.682428255	0.17579797
CCNE1	5.17%	3.71%	1.415113094	0.17579797
CDKN2A	23.62%	20.54%	1.196464719	0.17579797
EZH2	0.00%	0.47%	0	0.17579797
BCORL1	2.02%	1.18%	1.730926691	0.178864155
IKBKE	1.12%	0.51%	2.199344104	0.178864155
PDGFRB	0.56%	0.19%	2.982421463	0.178864155
AKT3	1.46%	0.77%	1.914887617	0.180634601
CDK8	0.34%	1.05%	0.320583523	0.180634601
CRKL	1.69%	0.92%	1.853870611	0.180634601
CEBPA	0.45%	0.14%	3.245164669	0.210287666
PRKAR1A	0.56%	0.21%	2.714323149	0.221378742
FGF23	2.25%	1.41%	1.60474678	0.234790212
PAX5	1.01%	0.48%	2.109932394	0.234790212
TNFAIP3	0.56%	0.22%	2.591458043	0.243175616
CREBBP	3.15%	2.16%	1.471727142	0.246518844
CTNNA1	1.24%	0.68%	1.823986299	0.295141226
RAD52	0.11%	0.01%	16.59724099	0.295141226
HGF	0.79%	1.56%	0.500446666	0.356021084
MYC	10.12%	8.46%	1.218062674	0.363133907
NKX2-1	2.70%	1.85%	1.470525314	0.363133907
FGF4	5.74%	4.51%	1.289166969	0.393637024
RPTOR	0.79%	0.38%	2.073539796	0.394313299
PDGFRA	1.91%	1.25%	1.545590172	0.39654836
FGF19	5.96%	4.74%	1.273511771	0.406109344
CDH1	2.92%	2.13%	1.382499266	0.42167807
NFKBIA	2.59%	1.84%	1.414224313	0.42167807

Gene	Percent altered in *NTRK+*	Percent altered in *NTRK–*	Odds ratio	Corrected *P* -value (FDR)
SMAD2	0.22%	0.72%	0.309072235	0.425958531
ESR1	2.36%	1.64%	1.449653021	0.426822941
FANCG	0.45%	0.20%	2.219805137	0.426822941
ARAF	0.56%	0.29%	1.972217302	0.433140169
FBXW7	4.39%	3.42%	1.294472535	0.433140169
JAK3	0.34%	0.13%	2.542915304	0.433140169
BRCA1	2.92%	2.16%	1.367383827	0.462975952
DAXX	0.67%	0.34%	2.002314986	0.462975952
IRS2	1.57%	1.02%	1.552015957	0.462975952
TBX3	0.34%	0.81%	0.414599805	0.462975952
ERG	0.56%	1.12%	0.497770004	0.492607367
FGF3	5.62%	4.59%	1.237397436	0.492607367
SMO	0.45%	0.23%	1.990047025	0.492607367
MSH2	1.12%	0.71%	1.587890605	0.510123474
KDM5A	2.36%	1.75%	1.359575087	0.511426358
EPHB1	0.90%	0.54%	1.669188974	0.512420429
JAK1	0.67%	0.39%	1.720000391	0.541600557
CCND1	6.19%	5.25%	1.190866159	0.623365637
CHEK1	0.11%	0.03%	4.254865442	0.630689495
DDR2	0.22%	0.10%	2.321812348	0.630689495
GSK3B	0.11%	0.03%	4.148465653	0.630689495
MYCN	1.01%	0.67%	1.519385637	0.630689495
TMPRSS2	0.67%	1.15%	0.582000595	0.630689495
EP300	2.14%	1.63%	1.316875208	0.664662481
DIS3	0.22%	0.10%	2.162837071	0.677044162
BCL2L2	0.79%	0.53%	1.490833105	0.694544248
FGF10	0.90%	1.42%	0.631158576	0.701154163
CCND3	1.91%	1.46%	1.319194144	0.701164249
ERBB3	1.91%	1.44%	1.330197933	0.701164249
PBRM1	1.69%	2.29%	0.731804731	0.701164249
EWSR1	0.11%	0.40%	0.278778884	0.704170231
FGFR3	0.79%	1.22%	0.643212773	0.704170231
FLT3	0.79%	1.25%	0.627818754	0.704170231
MLH1	0.90%	0.63%	1.431629827	0.704170231
NF2	1.12%	1.65%	0.676272247	0.704170231
NOTCH1	1.91%	2.52%	0.754823405	0.704170231
PTEN	8.55%	9.70%	0.870352591	0.704170231
SPEN	1.57%	1.15%	1.372458051	0.704170231
ZNF703	2.47%	3.15%	0.77946457	0.704170231
GATA3	1.69%	1.28%	1.320561869	0.704631955
ATR	1.35%	0.99%	1.362963251	0.7060492
BCL2	0.45%	0.26%	1.748599465	0.7060492
CHEK2	1.24%	1.77%	0.695801255	0.7060492
HRAS	0.34%	0.69%	0.484468401	0.7060492
MRE11	0.45%	0.27%	1.671418115	0.713251143
AURKA	1.91%	1.53%	1.254500213	0.763644014
PPP2R1A	0.45%	0.80%	0.561723584	0.766534475
EGFR	5.17%	5.97%	0.859598065	0.782688734
NOTCH3	2.02%	1.66%	1.222861152	0.782688734
SUFU	0.45%	0.31%	1.472609518	0.782688734
AMER1	0.90%	1.30%	0.686925638	0.798453425
MUTYH	2.14%	1.77%	1.213837889	0.798453425
TSC2	1.24%	0.94%	1.317886226	0.807106235
RAD51	0.22%	0.15%	1.508388849	0.808422065
BARD1	0.56%	0.39%	1.430474197	0.813846561
BRCA2	3.37%	2.92%	1.160250809	0.813846561
CDK6	1.12%	1.53%	0.733385429	0.813846561
CIC	1.35%	1.09%	1.240927735	0.813846561
CTNNB1	2.70%	3.26%	0.822556994	0.813846561
DNMT3A	3.26%	2.85%	1.15122267	0.813846561

Gene	Percent altered in *NTRK+*	Percent altered in *NTRK–*	Odds ratio	Corrected *P* -value (FDR)
ERBB2	5.17%	5.85%	0.878150644	0.813846561
FGFR1	2.92%	3.48%	0.835569413	0.813846561
KDM6A	1.80%	2.27%	0.788533203	0.813846561
MAP2K4	1.91%	1.59%	1.203009217	0.813846561
MITF	0.56%	0.37%	1.508737429	0.813846561
RICTOR	2.70%	2.29%	1.181280392	0.813846561
TGFBR2	0.56%	0.40%	1.415782327	0.813846561
ERBB4	1.01%	0.80%	1.271603954	0.822112419
FLT1	0.34%	0.21%	1.596213535	0.822112419
SRC	1.01%	0.80%	1.272694874	0.822112419
FANCA	1.12%	0.93%	1.213034992	0.879069562
TET2	2.47%	2.16%	1.14798634	0.879069562
ZNF217	2.47%	2.14%	1.160637135	0.879069562
ARFRP1	1.80%	1.54%	1.169871201	0.88426687
KDR	1.35%	1.13%	1.196510073	0.905946816
MEN1	0.79%	0.64%	1.224069477	0.905946816
RAD51C	0.34%	0.27%	1.264911471	0.905946816
SMARCB1	0.79%	0.64%	1.233219654	0.905946816
STK11	3.94%	4.45%	0.879576773	0.905946816
CDKN1B	0.45%	0.70%	0.637896148	0.908753111
MAP2K1	0.45%	0.67%	0.665349819	0.908753111
MAP3K1	1.46%	1.23%	1.188982856	0.908753111
PALB2	0.45%	0.71%	0.633894515	0.908753111
ALK	0.79%	1.06%	0.742891273	0.913825898
ATM	3.26%	3.65%	0.8898591	0.913825898
AURKB	0.00%	0.14%	0	0.913825898
BAP1	1.57%	1.88%	0.834142754	0.913825898
BCL6	0.11%	0.10%	1.094475664	0.913825898
CBL	0.34%	0.54%	0.618514377	0.913825898
EMSY	1.80%	1.60%	1.12826407	0.913825898
EPHA3	0.79%	1.03%	0.760398259	0.913825898
FANCC	0.22%	0.42%	0.538577757	0.913825898
FOXL2	0.00%	0.14%	0	0.913825898
JAK2	1.01%	0.88%	1.146667219	0.913825898
KDM5C	1.01%	0.85%	1.190436624	0.913825898
KLHL6	0.11%	0.09%	1.232953213	0.913825898
MAP3K13	0.45%	0.38%	1.174569272	0.913825898
MED12	0.45%	0.37%	1.225739341	0.913825898
MET	1.69%	1.99%	0.84516469	0.913825898
MPL	0.00%	0.13%	0	0.913825898
NFE2L2	0.90%	1.17%	0.76569219	0.913825898
NOTCH2	1.57%	1.39%	1.136919091	0.913825898
PMS2	0.56%	0.47%	1.186157533	0.913825898
PTPN11	0.34%	0.51%	0.656329867	0.913825898
SETD2	1.69%	2.03%	0.829861378	0.913825898
SPOP	0.56%	0.49%	1.143446086	0.913825898
TNFRSF14	0.00%	0.13%	0	0.913825898
TSC1	1.12%	0.96%	1.169906723	0.913825898
AXL	0.67%	0.56%	1.201336872	0.915679853
GNAQ	0.22%	0.16%	1.411964787	0.916378409
PIK3C2G	0.00%	0.16%	0	0.916378409
MTOR	0.45%	0.65%	0.690871991	0.933565571
FGFR2	1.35%	1.59%	0.848191679	0.947418791
BRIP1	0.56%	0.74%	0.760940344	0.948004157
RAF1	0.56%	0.74%	0.7595331	0.948004157
AKT2	1.57%	1.81%	0.869246246	0.956574267
SOX2	1.69%	1.91%	0.881784482	0.962126448
RET	1.01%	0.93%	1.091295214	0.972012379
IKZF1	0.11%	0.27%	0.422301606	0.976669121
STAG2	1.12%	1.02%	1.099843864	0.976669121

Gene	Percent altered in *NTRK+*	Percent altered in *NTRK–*	Odds ratio	Corrected *P* -value (FDR)
CDKN2C	0.34%	0.28%	1.200655145	0.976941501
FGFR4	0.34%	0.29%	1.182065436	0.976941501
PRDM1	0.34%	0.30%	1.125743082	0.977030987
REL	0.34%	0.30%	1.119392525	0.977030987
CUL4A	1.01%	1.22%	0.828400556	0.977547283
CDC73	0.22%	0.39%	0.578254555	0.991540236
CSF1R	0.22%	0.37%	0.615480526	0.991540236
IDH1	1.80%	1.70%	1.060063607	0.991540236
IDH2	0.22%	0.38%	0.597098862	0.991540236
KIT	1.80%	1.68%	1.070495445	0.991540236
WT1	0.45%	0.42%	1.071708515	0.991540236
ABL1	0.22%	0.26%	0.879287917	1
AKT1	1.12%	1.28%	0.880792431	1
ALOX12B	0.00%	0.03%	0	1
AR	0.79%	0.89%	0.879275993	1
ARID1A	8.32%	8.47%	0.981555667	1
BCOR	1.46%	1.44%	1.018877427	1
BTG1	0.00%	0.04%	0	1
BTK	0.00%	0.05%	0	1
CARD11	0.11%	0.20%	0.570246236	1
CBFB	0.22%	0.23%	0.978103913	1
CD79B	0.00%	0.01%	0	1
CTCF	0.90%	0.92%	0.979047854	1
DOT1L	0.00%	0.05%	0	1
ETV4	0.00%	0.03%	0	1
ETV5	0.00%	0.02%	0	1
FAM46C	0.00%	0.03%	0	1
FANCL	0.11%	0.16%	0.68617686	1
FGF12	1.35%	1.31%	1.02750789	1
FGF14	0.45%	0.54%	0.834504547	1
GNA11	0.11%	0.16%	0.720542009	1
GNA13	0.00%	0.06%	0	1
GNAS	3.37%	3.37%	1.002065864	1
IRF4	0.00%	0.04%	0	1
KEAP1	1.80%	1.87%	0.961139395	1
KMT2A	0.11%	0.15%	0.770890949	1
MAP2K2	0.00%	0.10%	0	1
MEF2B	0.00%	0.03%	0	1
MYCL	0.79%	0.82%	0.964053257	1
MYD88	0.11%	0.15%	0.74823582	1
NF1	5.62%	5.59%	1.006863038	1
NPM1	0.11%	0.12%	0.950069697	1
PARP2	0.00%	0.01%	0	1
PARP3	0.00%	0.01%	0	1
PDK1	0.00%	0.01%	0	1
PIK3R1	2.47%	2.65%	0.932026606	1
RAD51B	0.22%	0.25%	0.883989478	1
RAD51D	0.11%	0.16%	0.68617686	1
RAD54L	0.00%	0.04%	0	1
RARA	0.00%	0.06%	0	1
RB1	7.09%	7.29%	0.969922766	1
ROS1	0.34%	0.36%	0.932964946	1
SF3B1	1.01%	1.12%	0.899234883	1
SMARCA4	3.04%	2.95%	1.030728048	1
SOCS1	0.00%	0.03%	0	1
SYK	0.00%	0.04%	0	1
TIPARP	0.00%	0.04%	0	1
XPO1	0.11%	0.11%	0.989821164	1

FDR, false discovery rate; *NTRK* , neurotrophic tyrosine receptor kinase.

Supplementary Table 10. Mutations found within driver genes in *NTRK* + and *NTRK* – colorectal cancer (CRC), breast cancer and non-small cell lung cancer (NSCLC)

Indication	Geneset	Total assessable samples	Percent altered in *NTRK* +	Percent altered in *NTRK* –	*NTRK* + Geneset(+)	*NTRK* + Geneset(–)	*NTRK* – Geneset(+)	*NTRK* – Geneset(–)	OR	Uncorrected p-value	Corrected p-value (fdr)
CRC	CRC_set	34697	29.87%	67.32%	23	54	23305	11315	0.206794759	2.32425E-11	1.39455E-10
Breast	Breast_set	30182	51.69%	64.78%	61	57	19476	10588	0.581793877	0.0036755	0.007351
NSCLC	NSCLC_set	56615	48.53%	63.13%	66	70	35655	20824	0.550667708	0.000663511	0.001990532

Supplementary Table 11. Summary of co-occurrence and mutual exclusivity of driver gene mutations and microsatellite instability high (MSI-H) status with specific *NTRK* fusion-positive cancers

Indication	Marker	Total assessable samples	Percent altered in *NTRK* +	Percent altered in *NTRK* –	*NTRK* + Marker(+)	*NTRK* + Marker(–)	*NTRK* – Marker(+)	*NTRK* – Marker(–)	OR	Uncorrected p-value	Corrected p-value (fdr)
Melanoma	Melanoma_set	8083	50.00%	60.51%	10	10	4879	3184	0.652592744	0.364984529	0.437981435
Glioma	Glioma_set	10955	28.26%	23.46%	13	33	2559	8350	1.285421625	0.484952141	0.484952141
CRC	CRC_set	34697	29.87%	67.32%	23	54	23305	11315	0.206794759	2.32425E-11	1.39455E-10
Breast	Breast_set	30182	51.69%	64.78%	61	57	19476	10588	0.581793877	0.0036755	0.007351
Ovary	Ovary_set	16522	35.29%	23.64%	18	33	3894	12577	1.761731335	0.06757846	0.10136769
NSCLC	NSCLC_set	56615	48.53%	63.13%	66	70	35655	20824	0.550667708	0.000663511	0.001990532
Melanoma	MSI-H	7206	0.00%	0.04%	0	18	3	7185	0	1	1
Glioma	MSI-H	9479	0.00%	0.17%	0	39	16	9424	0	1	1
CRC	MSI-H	31581	61.84%	4.50%	47	29	1417	30088	34.41306305	2.22627E-43	1.33576E-42
Breast	MSI-H	26332	0.00%	0.36%	0	109	95	26128	0	1	1
Ovary	MSI-H	15163	0.00%	0.65%	0	48	98	15017	0	1	1
NSCLC	MSI-H	50997	0.00%	0.33%	0	126	170	50701	0	1	1
NSCLC	Tobacco mutational signature	48617	13.01%	14.21%	16	107	6889	41605	0.903078591	0.79645226	0.79645226

Supplementary Table 12. Evaluation of and microsatellite instability (MSI) status in *NTRK* fusion-positive versus *NTRK* fusion-negative solid tumours

Indication	*NTRK* Status	MSI assessable count	MSI Status	Count MSI Status positive in *NTRK* status	Percent MSI Status positive in *NTRK* status
CRC	*NTRK*+	76	MSI-H	47	61.84%
CRC	*NTRK*+	76	MSI ambiguous	0	0.00%
CRC	*NTRK*+	76	MSS	29	38.16%
CRC	*NTRK*–	31505	MSI-H	1417	4.50%
CRC	*NTRK*–	31505	MSI ambiguous	158	0.50%
CRC	*NTRK*–	31505	MSS	29930	95.00%
all	*NTRK*+	827	MSI-H	54	6.53%
all	*NTRK*+	827	MSI ambiguous	6	0.73%
all	*NTRK*+	827	MSS	767	92.74%
all	*NTRK*–	264773	MSI-H	4452	1.68%
all	*NTRK*–	264773	MSI ambiguous	1736	0.66%
all	*NTRK*–	264773	MSS	258585	97.66%
non-CRC	*NTRK*+	751	MSI-H	7	0.93%
non-CRC	*NTRK*+	751	MSI ambiguous	6	0.80%
non-CRC	*NTRK*+	751	MSS	738	98.27%
non-CRC	*NTRK*–	233268	MSI-H	3035	1.30%
non-CRC	*NTRK*–	233268	MSI ambiguous	1578	0.68%
non-CRC	*NTRK*–	233268	MSS	228655	98.02%

Supplementary Table 13. Genes with significantly co-occurring or mutually exclusive alterations with *NTRK* fusions in all MSI-H CRC (a) and in spontaneous MSI-H CRC (b)

a Significantly co-occurring or mutually exclusive alterations with *NTRK* gene fusions in all MSI-H CRC

Gene	*NTRK*+ Gene+	*NTRK*+ Gene-	*NTRK*- Gene+	*NTRK*- Gene-	Odds ratio	Co-occurrence/ mutual exclusivity	*P*-value	FDR
NTRK1	40	7	0	1389	NA	NA	$4.59x10^{-71}$	$4.96x10^{-69}$
BRAF	1	46	600	789	0.028587	Mutual exclusivity	$2.54x10^{-10}$	$1.37x10^{-08}$
KRAS	2	45	419	970	0.10289	Mutual exclusivity	$2.46x10^{-5}$	0.000884
PIK3CA	5	42	500	889	0.211667	Mutual exclusivity	0.000149	0.004032
RNF43	39	8	805	584	3.536646	Co-occurrence	0.00044	0.009498
CTNNB1	0	47	199	1190	0	Mutual exclusivity	0.001796	0.032324
APC	9	38	573	816	0.337283	Mutual exclusivity	0.002236	0.034503

b Significantly co-occurring or mutually exclusive alterations with *NTRK* gene fusions in spontaneous MSI-H CRC

Gene	*NTRK*+ Gene+	*NTRK*+ Gene-	*NTRK*- Gene+	*NTRK*- Gene-	Odds ratio	Co-occurrence/ mutual exclusivity	*P*-value	FDR
NTRK1	30	3	0	618	NA	NA	$1.12x10^{-48}$	$7.59x10^{-47}$
BRAF	1	32	380	238	0.019572	Mutual exclusivity	$3.9x10^{-12}$	$1.32x10^{-10}$

Co-occurrence refers to genes that occurred in *NTRK* fusion-positive disease with an odds ratio greater than 1 compared with *NTRK* fusion-negative disease and the false discovery rate (FDR)-adjusted *P*-value was <0.05. Mutual exclusivity refers to genes that did not occur in *NTRK* fusion-positive disease with an odds ratio less than 1 compared with *NTRK* fusion-negative disease and the FDR-adjusted *P*-value was <0.05.

CRC, colorectal cancer; MSI-H, microsatellite instability high; NA, not applicable; *NTRK*, neurotrophic tyrosine receptor kinase.

Supplementary Table 14. Comparisons of *NTRK* fusion-positive tumour types in entrectinib adult clinical studies[9] versus FoundationCORE database

Indication	Total count (FoundationCORE)	Count *NTRK*+ (FoundationCORE)	Percent *NTRK*+ (FoundationCORE)	Total count (ALKA, STARTRK-1, STARTRK-2)*	Count *NTRK*+ (ALKA, STARTRK-1, STARTRK-2)*	Percent *NTRK* + (ALKA, STARTRK-1, STARTRK-2)*
Sarcoma	598	114	19.06%	54	13	24.07%
NSCLC	598	136	22.74%	54	10	18.52%
MASC	598	8	1.34%	54	7	12.96%
Breast	598	117	19.57%	54	6	11.11%
Thyroid	598	29	4.85%	54	5	9.26%
CRC	598	77	12.88%	54	4	7.41%
Neuroendocrine	598	11	1.84%	54	3	5.56%
Pancreatic	598	28	4.68%	54	3	5.56%
Ovarian	598	51	8.53%	54	1	1.85%
Endometrial	598	16	2.68%	54	1	1.85%
Cholangiocarcinoma	598	11	1.84%	54	1	1.85%
*Doebele et al. Lancet Oncol 2020 [Table 1]						

Supplementary Table 15. Comparisons of patient demographics in entrectinib adult clinical studies[9] and the FoundationCORE database

	**ALKA, STARTRK-1, STARTRK-2 (Doebele et al.[9])** ***n* = 54 *NTRK* fusion-positive patients**	**FoundationCORE (only tumour types in Doebele et al.[9])** ***n* = 598 *NTRK* fusion-positive patients**	**FoundationCORE (excluding tumour types in Doebele et al.[9])** ***n* = 244 *NTRK* fusion-positive patients**
Median age, years (range)	58 (48–67)	61 (18–89)	62.5 (0–89)
Female, *n* (%)	32 (59)	393 (66)	96 (39)
Male, *n* (%)	22 (41)	205 (34)	148 (61)

NTRK, neurotrophic tyrosine receptor kinase.

Supplementary Figure 1. The prevalence of *NTRK* fusions by predicted genetic ancestry in solid tumours.

Error bars show 95% confidence intervals.

NTRK, neurotrophic tyrosine receptor kinase.

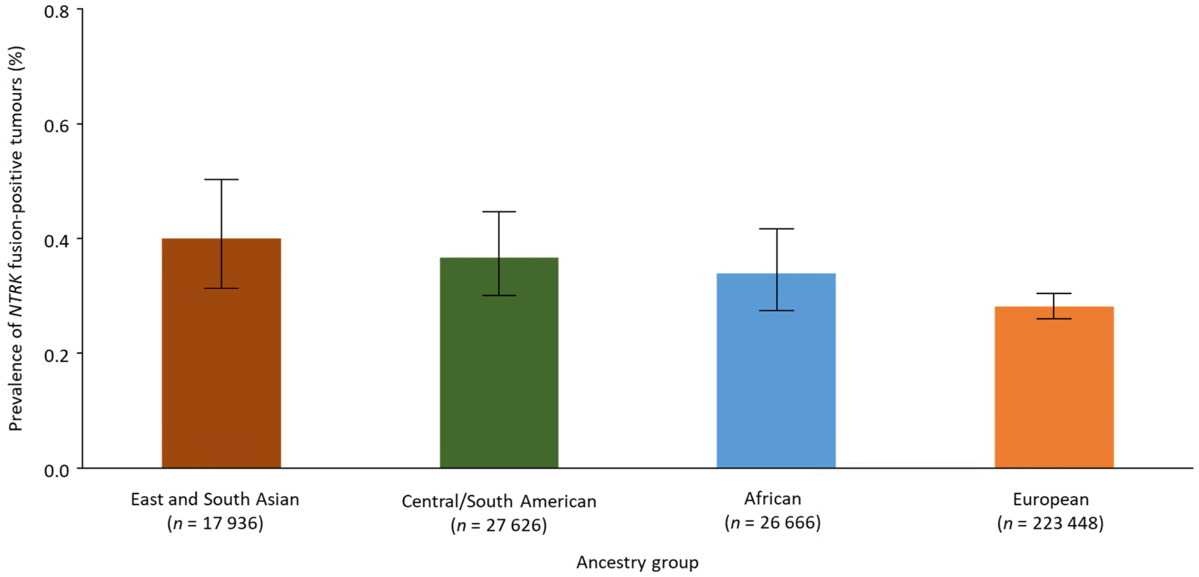

Tumour mutational burden in *NTRK* fusion-positive tumours

Median TMB was similar in *NTRK* fusion-positive and fusion-negative tumours (median 3.5 mutations/megabase for both), but was increased in *NTRK* fusion-positive CRC (median 29.6 mutations per megabase; supplementary Figure 2).

Supplementary Figure 2. Evaluation of tumour mutational burden (TMB) in *NTRK* fusion-positive versus *NTRK* fusion-negative solid tumours.

CRC, colorectal cancer; NSCLC, non-small cell lung cancer; *NTRK*, neurotrophic tyrosine receptor kinase.

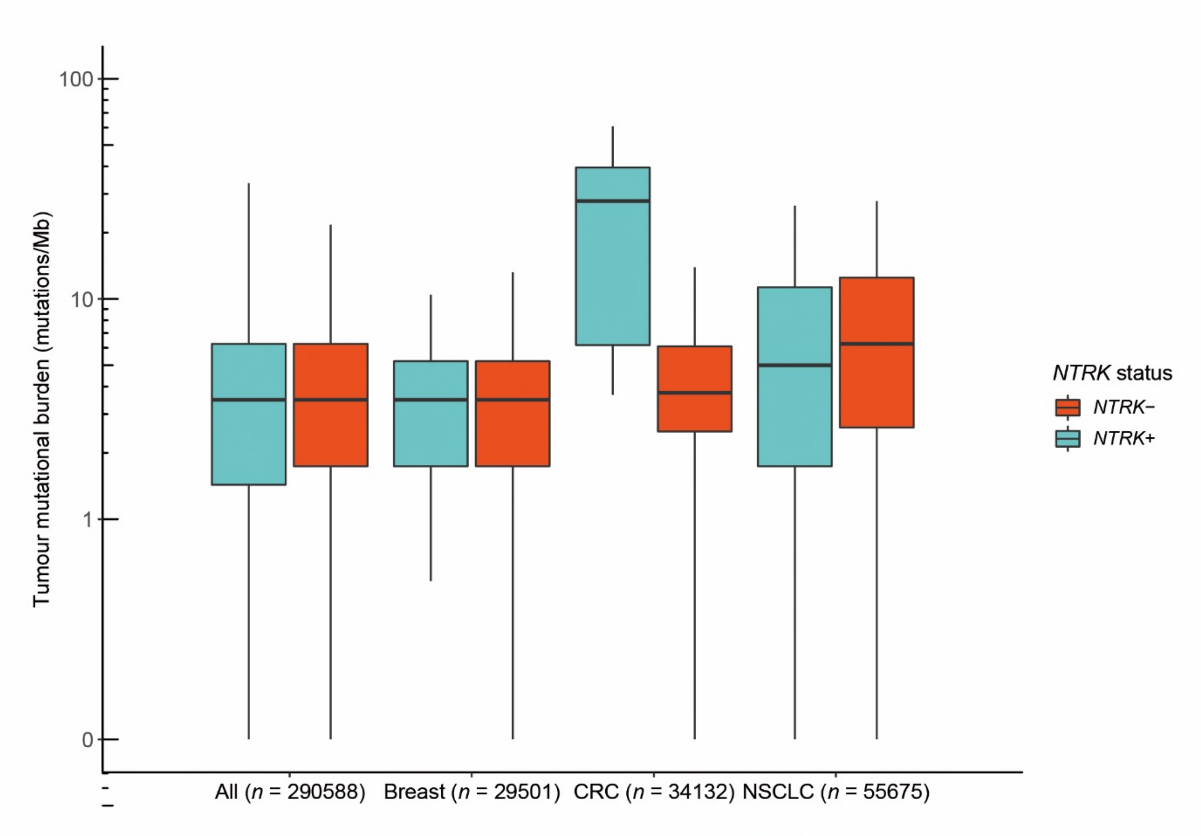

Supplement: Supplementary file 1 — Supplementary Information [file 41698_2021_206_MOESM1_ESM.pdf]
