# Supplementary material for: Genomic context of NTRK1/2/3 fusion-positive tumours from a large real-world population
Source: NPJ Precis Oncol. 2021 Jul 20;5:69. doi: 10.1038/s41698-021-00206-y (PMC8292342; doi:10.1038/s41698-021-00206-y)
Supplement: Supplementary file 2 — Reporting Summary [file 41698_2021_206_MOESM2_ESM.pdf]

nature research

Corresponding author(s): Dr C. Benedikt Westphalen

Last updated by author(s): Apr 22, 2021

Reporting Summary

Nature Research wishes to improve the reproducibility of the work that we publish. This form provides structure for consistency and transparency in reporting. For further information on Nature Research policies, see our Editorial Policies and the Editorial Policy Checklist.

Statistics

For all statistical analyses, confirm that the following items are present in the figure legend, table legend, main text, or Methods section.

n/a	Confirmed	
☐	☒	The exact sample size (n) for each experimental group/condition, given as a discrete number and unit of measurement
☒	☐	A statement on whether measurements were taken from distinct samples or whether the same sample was measured repeatedly
☐	☒	The statistical test(s) used AND whether they are one- or two-sided<br>*Only common tests should be described solely by name; describe more complex techniques in the Methods section.*
☐	☒	A description of all covariates tested
☐	☒	A description of any assumptions or corrections, such as tests of normality and adjustment for multiple comparisons
☒	☐	A full description of the statistical parameters including central tendency (e.g. means) or other basic estimates (e.g. regression coefficient) AND variation (e.g. standard deviation) or associated estimates of uncertainty (e.g. confidence intervals)
☒	☐	For null hypothesis testing, the test statistic (e.g. F, t, r) with confidence intervals, effect sizes, degrees of freedom and P value noted<br>*Give P values as exact values whenever suitable.*
☒	☐	For Bayesian analysis, information on the choice of priors and Markov chain Monte Carlo settings
☒	☐	For hierarchical and complex designs, identification of the appropriate level for tests and full reporting of outcomes
☒	☐	Estimates of effect sizes (e.g. Cohen's d, Pearson's r), indicating how they were calculated

Our web collection on statistics for biologists contains articles on many of the points above.

Software and code

Policy information about availability of computer code

Data collection	na
Data analysis	na

For manuscripts utilizing custom algorithms or software that are central to the research but not yet described in published literature, software must be made available to editors and reviewers. We strongly encourage code deposition in a community repository (e.g. GitHub). See the Nature Research guidelines for submitting code & software for further information.

Data

Policy information about availability of data

All manuscripts must include a data availability statement. This statement should provide the following information, where applicable:

- Accession codes, unique identifiers, or web links for publicly available datasets
- A list of figures that have associated raw data
- A description of any restrictions on data availability

Qualified researchers may request access to individual patient-level data from the clinical trials through the clinical study data request platform (https://vivli.org/). Further details on Roche's criteria for eligible studies are available here (https://vivli.org/members/ourmembers/). For further details on Roche's Global Policy on the Sharing of Clinical Information and how to request access to related clinical study documents, see here (https://www.roche.com/research_and_development/who_we_are_how_we_work/clinical_trials/our_commitment_to_data_sharing.htm). As the Foundation Research dataset contains patient genomic information that, depending on the prevalence of the identified alterations, could be used to identify individuals, the patient-level data cannot be released.

Field-specific reporting

Please select the one below that is the best fit for your research. If you are not sure, read the appropriate sections before making your selection.

☒ Life sciences ☐ Behavioural & social sciences ☐ Ecological, evolutionary & environmental sciences

For a reference copy of the document with all sections, see nature.com/documents/nr-reporting-summary-flat.pdf

Life sciences study design

All studies must disclose on these points even when the disclosure is negative.

Sample size	Sample size for the FoundationCORE database was determined from the number of samples available: data from 295,676 de-identified, consented-for-research cases between January 2013 and December 2019 from 75 different solid tumour types were profiled. Sample size for the clinical trials population was the efficacy-evaluable population, i.e., all patients who had received at least one dose of entrectinib and had at least 6 months of follow up.
Data exclusions	As stated in the manuscript: Variants removed from the dataset included germline variants (1000 Genomes Project [dbSNP142] or dbSNP database http://www.ncbi.nlm.nih.gov/SNP/), those with ≥2 counts in the ExAC database (http://exac.broadinstitute.org/) except for known cancer drivers (e.g., BRCA1/2 and TP53 mutations), and recurrent variants of unknown significance predicted to be germline by an internally developed algorithm.
Replication	Not applicable to this study as this is a retrospective comparison of data previously entered in the FoundationCORE database and of NTRK fusion status of NTRK+ patients previously enrolled in one of three entrectinib clinical trials
Randomization	Not applicable to this study as this is not a randomised analysis
Blinding	Not applicable to this study as it is a retrospective analysis of data from a database and samples from clinical trials where all patients received the same treatment

Reporting for specific materials, systems and methods

We require information from authors about some types of materials, experimental systems and methods used in many studies. Here, indicate whether each material, system or method listed is relevant to your study. If you are not sure if a list item applies to your research, read the appropriate section before selecting a response.

Materials & experimental systems

n/a	Involved in the study	
☒	☐	Antibodies
☒	☐	Eukaryotic cell lines
☒	☐	Palaeontology and archaeology
☒	☐	Animals and other organisms
☒	☐	Human research participants
☐	☒	Clinical data
☒	☐	Dual use research of concern

Methods

n/a	Involved in the study	
☒	☐	ChIP-seq
☒	☐	Flow cytometry
☒	☐	MRI-based neuroimaging

Clinical data

Policy information about clinical studies

All manuscripts should comply with the ICMJE guidelines for publication of clinical research and a completed CONSORT checklist must be included with all submissions.

Clinical trial registration	ALKA-372-001 [EudraCT 2012-000148-88], STARTRK-1 [NCT02097810], STARTRK-2 [NCT02568267]
Study protocol	The redacted protocols of these three clinical trials have previously been published by Lancet Oncol (Doebele, et al. Lancet Oncol 2020).
Data collection	This is a secondary analysis of data from these clinical trials. Full methods have been published previously Doebele, et al. Lancet Oncol 2020).
Outcomes	This study did not analyse any endpoint from the clinical trials protocol but was a secondary analysis to compare NTRK fusion prevalence in a population enrolled in a clinical trial versus patients included in the FoundationCORE database
